# An 18-gene signature of recurrence-associated endothelial cells predicts tumor progression and castration resistance in prostate cancer

**DOI:** 10.1038/s41416-024-02761-0

**Published:** 2024-07-12

**Authors:** Bing-Biao Lin, Qingqing Huang, Binyuan Yan, Mingcheng Liu, Zhiqian Zhang, Hanqi Lei, Ronghua Huang, Jin-Tang Dong, Jun Pang

**Affiliations:** 1 Department of Urology, Kidney and Urology Center, Pelvic Floor Disorders Center, The Seventh Affiliated Hospital, Sun Yat-sen University, Shenzhen, Guangdong 518000 China; 2 Department of Human Cell Biology and Genetics, School of Medicine, Southern University of Science and Technology, 1088 Xueyuan Blvd, Shenzhen, 518055 China; 3 Department of Radiotherapy, Cancer Hospital of Shantou University Medical College, Shantou, Guangdong 515041 China; 4 The First Affiliated Hospital of Shantou University Medical College, Shantou, Guangdong 515000 China

**Keywords:** Tumour biomarkers, Prostate cancer, Cancer microenvironment, Machine learning, Predictive medicine

## Abstract

**Background:**

The prognostic and therapeutic implications of endothelial cells (ECs) heterogeneity in prostate cancer (PCa) are poorly understood.

**Methods:**

We investigated associations of EC heterogeneity with PCa recurrence and castration resistance in 8 bulk transcriptomic and 4 single-cell RNA-seq cohorts. A recurrence-associated EC (RAEC) signature was constructed by comparing 11 machine learning algorithms through nested cross-validation. Functional relevances of RAEC-specific genes were also tested.

**Results:**

A subset of ECs was significantly associated with recurrence in primary PCa and named RAECs. RAECs were characteristic of tip and immature cells and were enriched in migration, angiogenesis, and collagen-related pathways. We then developed an 18-gene RAEC signature (RAECsig) representative of RAECs. Higher RAECsig scores independently predicted tumor recurrence and performed better or comparably compared to clinicopathological factors and commercial gene signatures in multiple PCa cohorts. Of the 18 RAECsig genes, FSCN1 was upregulated in ECs from PCa with higher Gleason scores; and the silencing of FSCN1, TMEME255B, or GABRD in ECs either attenuated tube formation or inhibited PCa cell proliferation. Finally, higher RAECsig scores predicted castration resistance in both primary and castration-resistant PCa.

**Conclusion:**

This study establishes an endothelial signature that links a subset of ECs to prostate cancer recurrence and castration resistance.

## Introduction

Prostate cancer (PCa) is the most commonly diagnosed cancer and the second leading cause of cancer deaths in males in the United States [1]. Whereas patients with localized PCas can be effectively treated with radical prostatectomy or radiotherapy, around 27–53% of them undergo biochemical relapse [2, 3]. Relapsed PCa patients are usually treated with androgen deprivation therapy (ADT) [3], but many of them develop metastatic castration-resistant PCa (mCRPC) with a dismal prognosis [4, 5]. Hence, it is critical to identify PCa patients at risk of developing recurrent or castration-resistant cancers. Prostate-specific antigen (PSA), Gleason scores, and the TNM staging system are currently used to assess the risk of PCa and guide treatment options [6]. However, these clinicopathological factors cannot accurately reflect the heterogeneity within and between tumors and thus have difficulty segregating high-risk PCa from low-risk ones, rendering a subset of patients at risk of overtreatment or undertreatment [7, 8].

Genetic classifiers have been developed to better predict the risk of tumor progression, and some are commercially available for improving the risk stratification of PCa and guiding treatment decisions [9–12]. Nevertheless, the usefulness of these classifiers in predicting patient outcomes after ADT is somewhat limited. In addition, most classifiers were derived from bulk sequencing data, so they mainly reflect the impact of tumor cell heterogeneity on prognosis and do not take into account the tumor microenvironment (TME)’s effects, which also plays a critical role in tumor progression and therapeutic response [13, 14].

Single-cell RNA sequencing (scRNA-seq) is a powerful tool for addressing the impacts of TME heterogeneity at a genome-wide scale. It should be thus very useful in identifying gene signatures representative of rare cell populations that may be diluted in bulk RNA sequencing [15]. Tumor endothelial cells (TECs) constitute a key component of the TME, as they line the malformed tumor vasculature and demonstrate notable morphological and genetic changes compared to normal ECs [16]. Based on scRNA-seq studies, it appears that TECs are highly heterogeneous and likely to mediate tumor cell behaviors [17], tumor angiogenesis [18, 19], immune cell recruitment [18, 20], and therapeutic resistance [21].

Anti-angiogenic agents have been developed to target tumor angiogenesis, and such treatments are successful in renal and liver tumors [22, 23]. In PCa, however, while increased microvessel densities have been linked to tumor progression [24–26], therapies targeting VEGF/VEGFR have limited efficacy in treating advanced PCa [27, 28]. It is thus necessary to characterize the role of TECs in PCa progression at a finer resolution.

This study identified recurrence-associated ECs (RAECs) by integrating survival and RNA-seq data from the TCGA database with scRNA-seq data from 12 primary PCa specimens using the Scissor algorithm. Based on gene markers of RAECs, a RAEC signature (RAECsig) was constructed and validated to predict recurrence in 1036 PCa patients from 6 independent cohorts. Further analyzes suggest that the RAECsig is associated with castration resistance.

## Materials and methods

### Collection and processing of bulk sequencing data

mRNA expression data and corresponding clinical information of the TCGA-PRAD cohort (*n* = 488) [29] were downloaded from XENA (https://xena.ucsc.edu/) in November 2020. A total of 5 bulk RNA-seq datasets, including DKFZ-PRAD (*n* = 82) [30], GSE70769 (*n* = 92) [31], GSE70768 (*n* = 111) [31], GSE94767 (*n* = 132) [32], and GSE210i34 (*n* = 131) [33] were collected from the Gene Expression Omnibus (GEO, https://www.ncbi.nlm.nih.gov/geo/) for external validation. We used disease-free survival data for TCGA-PRAD and biochemical recurrence-free survival data for the 5 external cohorts. We only included primary PCa samples with available survival information. In cases where multiple samples come from the same patients, we randomly keep one sample for subsequent analyzes. We also applied the combat function in the “sva” R package to remove the batch effect of 4 microarray datasets and merged them into a Meta-Cohort. The polyA mRNA and clinical data of the SU2C-PRAD cohort [34], which were obtained from cBioportal (https://www.cbioportal.org/), and the RNA-seq and clinical data of GSE197780 [35] were used to investigate an association with castration resistance. For RNA sequencing data, we used log2 (transcripts per kilobase million+1) to represent the expression levels of RNA; while for raw expression data from microarrays, we performed background adjustment and normalization using the robust multiarray average algorithm (“affy” R package). Detailed information on published cohorts is listed in Tables S1 and S2.

### Acquisition and analysis of 10× human single-cell RNA-seq data

A total of 4 PCa scRNA-seq datasets were curated. The filtered count matrix and metadata of 14 untreated primary PCa samples were from the study by Ge et al. [36]; the filtered count matrix of 5 metastatic castration-resistant PCa (mCRPC) was from the study by Wang et al. [37]; the raw counts of 12 untreated primary PCa samples were from the study by Chen et al. [17]; and the FASTQ data of 14 mCRPC samples were from the study by Chan et al. [38]. CellRanger (v.7.1.0) was used to align reads of the Chan dataset to the Human reference (GRCh38) transcriptome. The “Seurat” package was used for subsequent single-cell analysis. We first analyzed each gene expression matrix independently by filtering out cells with library sizes smaller than the medians of the cell library sizes—3 × median absolute deviation (MAD), the number of expressed genes < the medians of total numbers of expressed genes—3 × MAD, and mitochondrial percent > the medians of mitochondrial percent + 3 × MAD. Doublets were marked for each sample using Scrublet.

We then used the LogNormalize function to normalize raw counts and performed principle component analysis on the 2000 most variable genes for dimensionality reduction. The first 50 principle components (PCs) were selected for graph-based Louvain clustering and Uniform Manifold Approximation and Projection (UMAP) visualization. Major cell types were annotated using canonical cell markers. Clusters that expressed markers of different major cell types were considered contaminated and removed. Clusters that could not be unambiguously identified with a cell type were classified as undetermined. We chose the first 20–30 PCs for transcriptomic analysis of ECs.

To create an EC atlas of primary PCa and mCRPC, we combined samples with more than 50 vascular ECs and corrected the batch effect using the Harmony algorithm. EC annotation was performed based on differentially expressed genes (DEGs) and markers from previous studies [18–20].

### Identification of recurrence-associated endothelial cells (RAECs) using the scRNA-seq dataset

Most scRNA datasets lack prognostic data, so we employed the Scissor algorithm (“Scissor” R package) [39], which exploits phenotypic information from bulk RNA-seq cohorts, to identify the most phenotype-relevant cell subpopulations from scRNA data. Specifically, we utilized the recurrence and transcriptomic data from the TCGA-PRAD dataset to identify the subset of ECs significantly associated with PCa recurrence in the Chen dataset using Scissor with a Cox regression model. A reliability significance test was performed using 10-fold cross-validation (CV) and 100 bootstraps to determine whether such an association was reliable.

### Identification of differentially expressed genes

Differential expression analysis of scRNA-seq was performed using MAST implemented in the Seurat FindMarker function (min.pct = 0.1). We considered genes significantly differentially expressed between ECs and non-ECs if the adjusted *P* value was < 0.05 and the absolute log fold-change (FC) was ≥ 0.5. Between RAECs and non-RAECs, genes were considered differentially expressed if the adjusted *P* value was < 0.05 and the absolute log FC was ≥ 0.25. The R package “Limma” was used to analyze genes’ differential expression between RAECsig-high and RAECsig-low tumors in bulk RNA-seq datasets. A meta-analysis with a random effect model was further conducted to obtain a consensus list of differentially expressed genes across datasets.

### Identification of genes defining recurrence-associated endothelial cells

Genes upregulated in ECs were first intersected with genes differentially expressed between RAECs and non-RAECs. To ensure that the selected DEGs were more specific to ECs, we adopted a similar threshold-based approach described by Patil et al. [13]. DEGs that had lower mRNA levels in non-ECs (average log(counts per million/100 + 1) < 1) and were upregulated in ECs by > 2 fold compared to non-ECs were retained. In addition, genes that had an average log(counts per million/100 + 1) ≥ 0.05 in epithelial cells were filtered out because the majority of cells in most tumor samples are epithelial cells. Lastly, univariate Cox regression analysis was performed to restrict genes to those associated with recurrence in TCGA-PRAD (*P* < 0.1).

### Inference of RAEC regulons

Single-cell regulatory network inference (SCENIC) [40], as implemented in pySCENIC, was used to analyze coexpression modules between transcription factors (TFs) and their putative target genes in ECs from the Chen dataset [17]. Default parameters and the hg38 RcisTarget database were used. Each regulon is defined as a TF and its target genes. A regulon’s specificity score was computed to infer RAEC-specific regulons based on the Jensen–Shannon divergence.

### Machine learning benchmark

To construct an accurate and robust recurrence-associated endothelial cell signature, we used nested CV to benchmark 11 machine learning algorithms, including elastic network (Enet), Ridge, least absolute shrinkage and selection operator (Lasso), stepwise Cox regression, partial least squares regression for Cox (plsRcox), CoxBoost, generalized boosted regression modeling (GBM), eXtreme gradient boosting survival (XGBoost), random survival forest (RSF), supervised principal components (SuperPC), and survival support vector machine (survival-SVM). Specifically, the TCGA-PRAD dataset was randomly split into 10 folds of approximately equal size. Each fold was used as a testing set, and the remaining 9 folds served as the training set. To optimize hyper parameters for each algorithm, we carried out a nested 5-fold CV on the training set. The range of hyper parameters tested is shown in Table S3. To ensure the model’s reliability, we kept the ratio between non-relapsed and relapsed patients constant in each fold. Eventually, each model was fitted on the training set with the hyper parameters that achieved the best performance during inner CV and was then evaluated on the testing set. The performance of the survival models was estimated by Harrell’s concordance index (C-index) and integrated Brier score (IBS) from the 10 testing sets. The model with the highest average C-index and lowest average IBS was considered optimal.

### Comparison of RAECsig to other prognostic signatures

We collected prognostic markers from 3 commercially available PCa genetic tests, including the Prolaris, Decipher, and OncotypeDX tests [9, 11, 12]. For each sample, we followed the previously described approach [41] to calculate a signature score. Specifically, PCA was performed on the scaled data of 31 genes for the Prolaris test, and the projection of each sample onto the first component is defined as a Prolaris-like score. A Decipher-like score was calculated using the same approach for the 20 genes included in the Decipher test (2 intergenic genes were not recovered and therefore removed from analysis). For the OncotypeDX test, we used the scaled data for 12 genes from Knezevic et al. [12] instead of the crossing point normalized by subtracting the aggregated expression of 5 reference genes. An OncotypeDX-like score was then calculated using the corresponding weighted coefficients for each gene and component and was min-max normalized and scaled to a range of 0 to 100 as follows:

$${{{\rm{OncotypeDX}}}}{\mbox{-}}{{{\rm{like}}}}\;{{{\rm{score}}}}=100\;\times({{{\rm{X}}}}-\min\{{{{\rm{X}}}}\})∣(\max\{{{{\rm{X}}}}\} - \min\{{{{\rm{X}}}}\}).$$

### Gene set enrichment analysis and pathway score calculation

Gene set enrichment analysis (GSEA) was conducted using the Hallmark and Gene Ontology gene sets (h.all.v7.4.symbols; c5.go.v2022.1.Hs.symbols; https://www.gsea-msigdb.org/gsea/msigdb/) using the “ClusterProfiler” R package. Default parameters were used for analysis. Pathways with adjusted *P* values < 0.05 and normalized enrichment score > 1.5 are considered significantly enriched. The cell cycle progression (CCP) score was calculated based on 31 genes related to cell cycle and cell proliferation [9]. The CRPCsig51 score was calculated using 51 genes to estimate the existence of castration-resistant tumor cells in primary PCa [42]. Androgen receptor (AR) activity was defined by 20 AR target genes [43]. Briefly, the gene expression values were converted to *z*-scores, and the *z*-scores of genes in the corresponding signatures were summed up for each sample, min-max normalized, and scaled to a range of 0–100.

The 54-gene score and 18-gene score were defined as:

$${{{{{\rm{Score}}}}}}={\sum }_{{{{\rm{i}}}}=1}{{{\rm{Z}}}}_{{{\rm{i}}}}-{\sum }_{{{{\rm{j}}}}=1}{{{\rm{Z}}}}_{{{\rm{j}}}}.$$

Where Z_i_ was the z-score for the *i*th upregulated gene in RAECs and Z_j_ was the z-score for the *j*th downregulated gene in RAECs.

The endothelial signature was defined by summing the z-scores of PECAM1, ENG, and VWF.

### Cell lines

PC-3 and 22Rv1 cell lines were purchased from American Type Culture Collection, whereas the human umbilical vein endothelial cell (HUVEC) line was purchased from the BeNa Culture Collection (BNCC, Beijing, China). All cells were authenticated by STR profiling and tested negative for mycoplasma contamination. All cells were cultured in RPMI-1640 medium supplemented with 10% fetal bovine serum (FBS, Biological Industries, HAMEK, Israel) and 1% penicillin/streptomycin (100 U/mL, Biological Industries).

### RNA interference

Small interfering RNAs (siRNAs) for *FSCN1*, *TMEM255B*, *GABRD*, and negative control were synthesized by Ribobio (Guangzhou, China). They were transfected into cells at 50 nM using the Lipofectamine RNAiMax reagent (Cat#: 13778150, Invitrogen) based on the manufacturer’s instruction. Forty-eight hours after transfection, total RNA was extracted from cells using the Eastep Super Total RNA Extraction Kit (Cat#: LS1040, Promega) and reverse-transcribed into cDNA using the HiScript III All-in-one RT SuperMix kit (Cat#: R333-01, Vazyme, Nanjing, China). The knockdown efficiency was evaluated using real-time qPCR with *GAPDH* as an internal reference. The siRNA sequences were as follows: siFSCN1, 5ʹ-GGUCAACAUCUACAGCGUCAC-3ʹ; siTMEM255B, 5ʹ-GGAUCUUUCUUAGGAAUUA-3ʹ; siGABRD-1, 5ʹ-CCACGGAGCUGAUGAACUU-3ʹ; and siGABRD-2, 5ʹ-ACAUGGACCUGGCCAAAUA-3ʹ. Real-time qPCR primers were provided in Table S4.

### Tube formation assay

HUVEC cells were seeded onto 6-well plates at 1 × 10^5^ cells per well. After attachment, cells were transfected with siRNAs targeting *FSCN1*, *TMEM255B*, *GABRD*, and the negative control for 48 h. Transfected HUVEC cells were collected, seeded onto 96-well plates (2 × 10^4^ per well) coated with growth factor reduced Matrigel (Cat #: 354230, Corning), and incubated for an additional 4 h. Images were then taken using a phase-contrast microscope with a 10× objective lens (Eclipse Ti2, Nikon, Tokyo, Japan), and tube formation was analyzed using the Image J software. At least 12 fields were measured for each group.

### Transwell assays

For cell migration analysis, HUVEC cells were harvested in serum-free medium 48 h after transfection with siRNAs and seeded onto the top chamber of 24-well transwell chambers (3.5 × 10^4^ cells per well). A total of 750 μL complete medium with 15% FBS was added to the lower chamber. After 24 h, cells on the lower side of the membrane were fixed with 4% paraformaldehyde, stained with purple crystal, and imaged by a stereoscope (Mshot, Guangzhou, China). Crystal violet was dissolved using 33% acetic acid, and optical densities at 570 nm were measured using a microplate reader (BioTek). The experimental procedure for the cell invasion assay was the same as for the cell migration assay, except that Matrigel (Cat #: 354234, Corning) was applied to the upper chamber.

### Annexin V-FITC/PI apoptosis detection assay

HUVEC cells were seeded onto 6-well plates at 3 × 10^5^ cells per well. After attachment, cells were transfected with siRNAs against TMEM255B and GABRD for 48 h. Transfected cells were harvested and resuspended by 1 × binding buffer. Then, Annexin V-FITC and PI reagents (Cat #: K2003, APExBIO) were added to each sample for 15 min at room temperature. Cells were then analyzed by BD Accuri^TM^ C6 Plus flow cytometer.

### Cell viability assay

After silencing *FSCN1*, *TMEM255B*, or *GABRD* in HUVEC cells for 36 h, cells were cultured in a medium with 1% serum for an additional 12 h. The conditional media were collected by centrifugation at 1200 rpm for 3 min. PC-3 and 22Rv1 cells were seeded onto 96-well plates at 3 × 10^3^ and 6 × 10^3^, respectively, cells per well in the mixture of conditional medium and complete medium (1:1). After 48 h of culture, 10 μL CCK-8 solution (Cat #: 40203ES76, Yeasen) was added to each well, cells were incubated for 2 h, and optical densities at 450 nm were then measured using a microplate reader (BioTek).

### Immunofluorescence staining

Clinical PCa samples were formalin-fixed, paraffin-embedded, and sectioned at 4 µm. Tissue slides were then deparaffinized in xylene, rehydrated in graded ethanol, and boiled in a citrate buffer (10 mM trisodium citrate, pH 6.0) in an airtight pressure cooker for 3 min to retrieve the antigen. The slides were then permeabilized using 0.3% Triton X-100, incubated with 10% goat serum at 4^o^C overnight, and then incubated with primary antibodies at room temperature for 2 h. The antibodies included FSCN1 (1:100, Cat #:14384-1-AP, Proteintech), TMEM255B (1:100, Cat #:PH9893, Abmart), GABRD (1:100, Cat #:PA5210S, Abmart), and CD31 (1:500, Cat #:3528 S, CST). Tissue sections were then incubated with the secondary antibody (Cat #: A23220, A24411, Abbkine) at room temperature for 30 min, and DAPI staining was then performed. Fluorescent images were taken using a fluorescence microscope (Eclipse Ni-U, Nikon, Tokyo, Japan).

### Statistical analysis

All statistical analysis and data visualization were conducted using GraphPad Prism (v.7.0) and R (v.4.2.0). *P* values of Kaplan–Meier curves were calculated using the log-rank test. Hazard ratios (HR) with a 95% confidence interval (CI) were computed using the Cox regression analysis (“Survival” R package). The time-dependent area under the receiver operating characteristic (ROC) was conducted using the “survivalROC” R package. The ROC for predicting binary categorical variables was performed using the “pROC” R package. The best cut off for RAECsig stratification was determined using the surv_cutpoint function of the “survminer” R package. C-index was computed using the “Hmisc” R package, and comparing C-indices between groups was performed using the “compareC” R package. IBS was calculated using the “survcomp” R package. Spearman’s rank-order correlation analysis was used to determine the correlation between continuous variables. Wilcoxon rank sum test was used for pair-wise comparison of continuous variables unless otherwise specified. For all boxplots, the lower and upper ends of boxes indicate the 25th and 75th percentiles, respectively, and the center lines represent the medians. All statistical tests were two-sided. *P* < 0.05 was considered statistically significant. All methods were carried out in accordance with the relevant guidelines and regulations.

## Results

### Identification and validation of recurrence-associated endothelial cells using a 54-gene panel

**Fig. 1:**
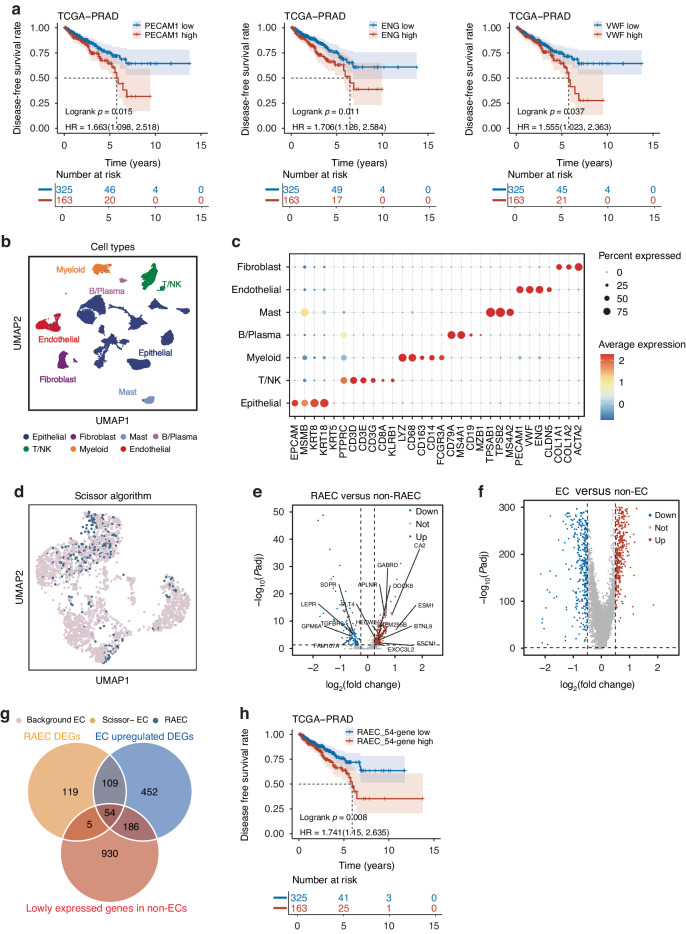
Recurrence-associated endothelial cells were identified and validated using an established 54-gene panel in primary prostate cancer. **a** Higher mRNA levels of *PECAM1*, *ENG*, and *VWF* are associated with worse prognosis in prostate cancer, as determined by the Kaplan–Meier analysis in the TCGA-PRAD cohort (top tertile versus the low and median tertiles). *P* values were calculated using the log-rank test. **b** UMAP plot of 7 major cell types using scRNA-seq data of 12 primary prostate cancer samples from the Chen study [17]. **c** Dotplot of marker genes’ expression levels of the major cell types displayed in panel **b**. The size of a dot indicates the percentage of cells that express corresponding genes, whereas the color of a dot reflects a gene’s expression level. **d** Scissor algorithm-inferred endothelial cells (ECs) associated with recurrence in the TCGA-PRAD cohort. Blue dots represent recurrence-associated ECs (RAECs), associated with a worse prognosis, whereas yellow dots represent ECs associated with a better prognosis. Gray dots mark ECs that do not show an association with prognosis. **e**, **f** Vocalnol plots of differentially expressed genes between RAECs and non-RAECs (i.e., Scissor-ECs and background ECs) and those between ECs and non-ECs (i.e., other major cell types as shown in panel **b**) **f** Red dots indicate upregulated genes, whereas blue dots indicate downregulated genes. **g** Venn diagrams of 54 RAEC-related genes, as identified by their differential expression between RAECs and non-RAECs, higher expression levels in ECs than non-ECs, and lower expression levels in non-ECs than ECs. **h** A higher score of the RAEC 54-gene panel (top tertile versus the rest) is correlated with a shorter recurrence time in the TCGA-PRAD cohort as determined by Kaplan–Meier analysis. The *P* value was derived from the Log-rank test.

As the first step in exploring the prognostic value of TECs in PCa, we estimated the abundance of TECs in primary tumors by calculating the expression levels of three classic endothelial markers, including *PECAM1*, *ENG*, and *VWF*, and investigated whether the TEC abundance is associated with tumor recurrence. In the TCGA-PRAD cohort, Kaplan–Meier analyzes demonstrated that tumors with higher expression levels of these markers had significantly higher recurrence rates (all log-rank *P* < 0.05; Fig. 1a). This observation is further supported by Cox regression analyzes, as tumors with a higher expression level for each of the three genes were at a significantly higher risk of developing recurrence (*PECAM1* HR: 1.663, 95% CI: 1.098-2.518; *ENG* HR: 1.706, 95% CI: 1.126–2.584; *VWF* HR: 1.555, 95% CI: 1.023–2.363). A significant association of higher *ENG* or *VWF* level with PCa recurrence was also detected in the DKFZ-PRAD cohort (Fig. S1). These results suggested that increased TECs are associated with tumor recurrence in primary PCa.

TECs are highly heterogeneous, so it is necessary to identify which subpopulation(s) of TECs is responsible for TEC’s association with tumor recurrence in PCa. To address this question, we first analyzed the Chen scRNA-seq dataset, which contains 12 untreated primary PCa samples from radical prostatectomy. After quality control, 29,457 cells were analyzed, and all major cell types were annotated based on specific markers (Fig. 1b, c). A total of 2457 ECs were annotated according to the expression of *PECAM1*, *VWF*, and *ENG*. We then leveraged the Scissor algorithm to integrate these ECs’ transcriptomics to the TCGA-PRAD cohort and reliably identified (*P* < 0.05) a subset of ECs (*n* = 347) whose expression profiles are associated with PCa recurrence. This subpopulation was hereafter referred to as recurrence-associated ECs (RAECs) (Fig. 1d).

We then applied differential gene analyzes to identify the genes whose expression profiles define RAECs and thus can be used to estimate RAEC abundance in bulk sequencing datasets. In total, 165 genes were upregulated and 122 downregulated in RAECs compared to the remaining ECs (non-RAECs) (Fig. 1e; Table S5); and 801 genes were upregulated in ECs compared to non-ECs (Fig. 1f; Table S6). Of those 801 genes, 163 were upregulated in both RAECs and ECs. To enhance the specificity of these genes to ECs, we used expression cutoffs to remove 109 genes that were also highly expressed in non-ECs, which left 54 RAEC-related genes (Fig. 1g).

In the TCGA-PRAD cohort, expression levels of the 54 RAEC-related genes were summed, and Kaplan–Meier and Cox regression analyzes were performed. PCa with higher scores of the 54 genes showed a significantly worse disease-free survival (log-rank *P* = 0.008, HR: 1.741, 95% CI:1.150–2.635; Fig. 1h). These findings confirm the robustness of the Scissor selection.

### RAECs are characteristic of tip ECs and increased angiogenic activities

To further characterize RAECs, we performed sub-clustering and fine annotation of ECs (Fig. S2A, b) in the Chen dataset, in which ECs were classified based on functional states instead of biological cell types [17]. We identified 7 biological subsets of ECs, including arterial defined by *FBLN5* and *ENPP2*; postcapillary vein (PCV) by *ACKR1*, and *SELP*; activated PCV by *POSTN* and *CCL14*; intermediate; immature by *APLNR*; and tip by *ESM1* and *APLN* (Fig. S2C). Arteries, PCVs, and capillaries are different EC subtypes belonging to traditional vascular beds. Activated PCV is previously identified in lung cancer and choroid neovascularization, and is considered to be the EC subtype from which neovessels originate [19]. Immature cells resemble stalk-like cells, which elongate vessel sprouts whereas Tip cells guide and navigate vessel sprouts during neovascularization [18, 19]. Intermediate cells are considered a plastic phenotype possibly transitioning from activated PCV to angiogenic cells [18, 19]. RAECs consisted of all annotated EC subtypes (Fig. S2D), tip cells were most abundant (*n* = 78), followed by immature cells (*n* = 60) and intermediate cells (*n* = 58). Compared to non-RAECs, RAECs contained more tip cells (22.5% vs. 14%), immature cells (17.3% vs. 8.7%), and intermediate cells (16.7% vs. 5.8%, Fig. S2E).

We then evaluated whether different EC subtypes have distinct gene expression profiles between RAECs and non-RAECs. Among the 5 subtypes of ECs, tip cells had the most DEGs (Fig. S2F). Specifically, tip cells in RAECs expressed higher levels of gene signatures associated with tip cell markers, migration, and extracellular matrix (ECM) modeling than their counterparts in non-RAECs (Fig. S2G). This finding suggests that a more differentiated state of tip cells plays the most important role in RAEC-associated PCa recurrence. GSEA demonstrated that RAECs expressed higher levels of tip cell markers, collagen, and VEGFRs (Fig. S2H) and were enriched in angiogenesis, migratory, and ECM modeling pathways compared to non-RAECs (Fig. S2I). For potential regulators of RAECs, the SCENIC analysis demonstrated that SOX4 and ZEB1 were the most specific regulons of RAECs (Fig. S2J). Consistently, compared to other subtypes of RAECs, tip cells had the highest mRNA levels of SOX4 and ZEB1 (Fig. S2F). Taken together, RAECs primarily contain tip cells and immature cells, and such tip cells are highly differentiated with pronounced angiogenic and ECM modeling activities.

### Development of a robust 18-gene recurrence-associated endothelial cell signature (RACEsig)

**Fig. 2:**
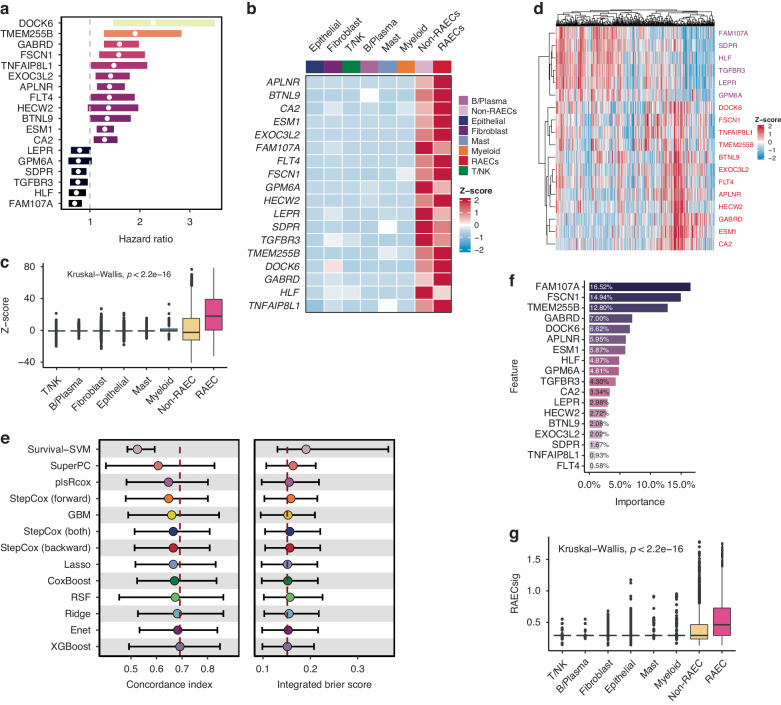
Development of an 18-gene signature of recurrence-associated endothelial cells. **a** Forest plot of 18 prognostic genes identified by univariate Cox regression analysis of 54 RAEC-related genes presented in Fig. 1g. *P* < 0.1 for all 18 genes. **b** Heat map of relative mRNA levels of the 18 prognostic RAEC-related genes across major cell types in the Chen dataset. **c** RAECs had the highest score when the 18 genes’ z-scores were summed for different types of cells. **d** Heat map of the 18 genes’ mRNA expression pattern in TCGA-PRAD. **e** C-index and IBS in outer 10-fold validation using the 11 hyperparameter-tuned models. Dots indicate each model’s mean of 10 C-indices or integrated Brier scores (IBS). The model with the highest average C-index and lowest average IBS was selected and termed recurrence-associated endothelial cell signature (RAECsig). **f** Bar plot showing feature importance of the 18 prognostic RAEC-related genes inferred by the eXtreme Gradient Boosting (XGBoost). Greater importance suggests more contributions to the XGBoost model when predicting PCa progression. **g** RAECsig scores among major cell types in the Chen dataset. SVM, support vector machine; Enet, elastic network; Lasso, Least Absolute Shrinkage and Selection Operator; plsRcox, partial least squares regression for Cox; StepCox, stepwise Cox regression; GBM, generalized boosted regression modeling; RSF, random survival forest; SuperPC, supervised principal components.

Since RAECs were prognostic for PCa recurrence, we sought to construct a robust gene signature that represents RAECs and can predict tumor recurrence. Univariate Cox regression analysis demonstrated that 18 of the 54 RAEC-specific genes were associated with disease-free survival (*P* < 0.1) in the TCGA-PRAD cohort (Fig. 2a and Table S7). Consistent with the RAEC characteristics described above, marker genes of tip cells (*ESM1*, *FSCN1*) and immature cells (*APLNR*) were among the 18 genes.

Each of the 18 RAEC genes was highly expressed in ECs, with 6 genes downregulated and 12 upregulated in RAECs (Fig. 2b). The z-scores for these 18 genes were summed across other major cell types, and the sum scores were the highest in the RAECs (*P* < 0.001, Fig. 2c), further indicating the power of these genes in distinguishing RAECs from other types of cells. The expression patterns of the 18 RAEC genes were confirmed in the Ge dataset (Fig. S3A), in which almost all RAEC genes showed higher mRNA levels in ECs than non-ECs, and the 18 genes’ scores were the highest in ECs (Fig. S3B, C).

Importantly, these prognostic genes derived from scRNA-seq data can be applied in the bulk RNA-seq data. By measuring gene coexpression and hierarchically clustering these genes’ coexpression patterns, we found that the mRNA expression levels of 12 upregulated RAEC genes were highly correlated with but separated from those of the 6 downregulated RAEC genes (Fig. 2d and S4). Therefore, the scRNA-seq-derived gene markers could be leveraged to infer RAEC’s abundance in bulk RNA-seq data.

Based on the 18 genes, we benchmarked 11 survival-related machine-learning algorithms through nested CV in the TCGA-PRAD. As shown in Fig. 2e and Table S8, the XGBoost survival model achieved the best performances with the highest mean C-index (0.692) and lowest mean IBS (0.151). This model with tuned hyper parameters was then fitted on the entire TCGA-PRAD dataset and termed RAECsig hereafter. The feature importance of the 18 genes is shown in Fig. 2f, where the top 5 features included *FAM107A*, *FSCN1*, *TMEM255B*, *GABRD*, and *DOCK6*. Using the RAECsig, we then calculated risk scores of different cell types in the Chen dataset and found that RAECs ranked first among all major cell types (*P* < 0.001, Fig. 2g), demonstrating that the RAECsig is indicative of RAECs. Furthermore, the RAECsig score was the highest in tip cells of RAECs (Fig. S5A) and could significantly discriminate them from other ECs (AUC = 0.844; Fig. S5B).

### RAECsig is an independent risk factor for primary PCa recurrence

**Fig. 3:**
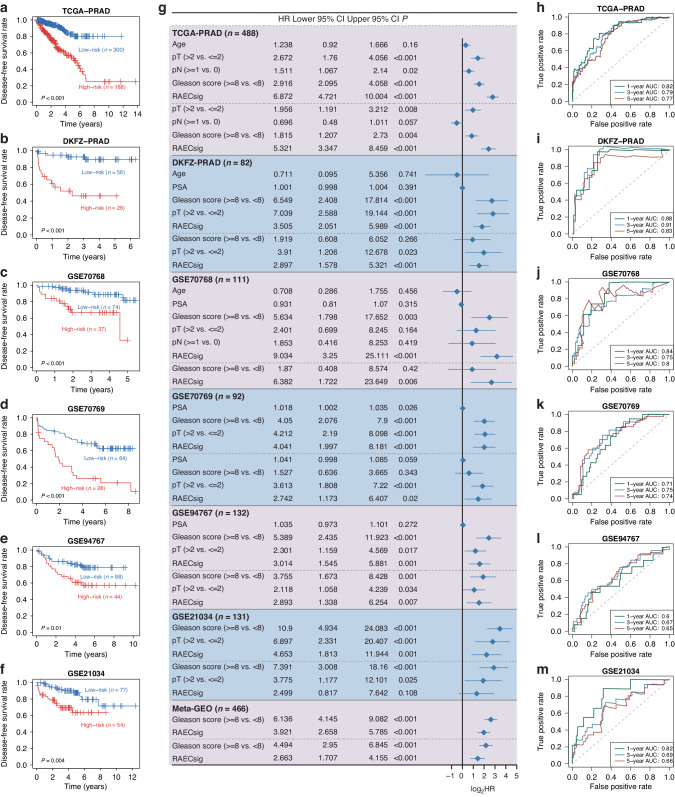
Independent validation of the 18-gene RAECsig in multiple PCa cohorts. **a**–**f** Recurrence-free survival of high- and low-risk groups stratified by a universe RAECsig value (0.58), including TCGA-PRAD (**a**), DKFZ-PRAD (**b**), GSE70768 (**c**), GSE70769 (**d**), GSE94767 (**e**), and GSE21034 (**f**). *P* values were derived from log-rank tests and were < 0.05. **g** Forest plot showing hazard ratio (HR) at 95% confidence interval (CI) and the corresponding *P* values of RAECsig and clinical and pathological characteristics using both the univariate (above the dashed lines) and the multivariate Cox regression analyzes (below the dashed lines) in 6 PCa cohorts. Only variables with a *P* value < 0.05 in univariate analyzes were included in multivariate analyzes. The Meta-Cohort consists of 4 PCa cohorts (panels **c**–**f**) in which gene expression was detected using the microarray platform. **h**–**m** AUROC curve analysis of RAECsig for predicting recurrence at 1, 3, and 5 years in the cohorts of TCGA-PRAD (**h**), DKFZ-PRAD (**i**), GSE70768(**J**), GSE70769(K), GSE94767(**l**), and GSE21034 (**m**). pT, pathological tumor stage; PSA, prostate-specific antigen; pN, pathological lymph node stage; AUROC, area under the receiver operating characteristic curve.

The prognostic value of the RAECsig was validated using 5 bulk sequencing datasets of primary PCa. The RAECsig risk score was calculated for each case, and the “survminer” package was used to define the optimal risk score threshold across various datasets. Using the cut off 0.58 derived from the GSE21034 cohort, we assigned patients to the high- and low-risk groups in each dataset. A higher RAECsig score was significantly associated with shorter disease-free survival in each cohort (log-rank *P* ≤ 0.01, Fig. 3a–f and S6A). Univariate Cox regression analyzes showed that the RAECsig was a risk factor for PCa recurrence in all cohorts (Fig. 3g). After adjusting for age, Gleason score, serum PSA, and TNM stage, RAECsig remained a statistically significant prognostic factor in the TCGA-PRAD (HR: 5.321, 95% CI: 3.347–8.459, *P* < 0.001), DKFZ-PRAD (HR: 2.897, 95% CI: 1.578–5.321, *P* < 0.001), GSE70768 (HR: 6.382, 95% CI: 1.722-23.649, *P* = 0.006), GSE70769 (HR: 2.742, 95% CI: 1.173–6.407, *P* = 0.02), GSE94767 (HR: 2.893, 95% CI: 1.338–6.254, *P* = 0.007), and Meta-Cohort (HR: 2.663, 95% CI: 1.707–4.155, *P* < 0.001) except in GSE21034 (HR: 2.499, 95% CI: 0.817–7.642, *P* = 0.108). Furthermore, time-dependent ROC analysis revealed a robust discrimination power of RAECsig in PCa recurrence. Specifically, the AUC values at 1, 3, and 5 years were 0.82, 0.79, and 0.77 in TCGA-PRAD; 0.88, 0.91, and 0.83 in DKFZ-PRAD; 0.84, 0.75, and 0.80 in GSE70768; 0.71, 0.75, and 0.74 in GSE70769; 0.60, 0.67, and 0.65 in GSE94767; 0.82, 0.69, and 0.66 in GSE21034; and 0.70, 0.70, and 0.68 in Meta-cohort (Fig. 3h–m and S6B). Taken together, we found that a universal RAECsig cut off can classify patients into either high- or low-risk groups and that RAECsig can be an independent risk factor for PCa recurrence.

### Comparison of RAECsig with clinicopathological features and commercial genetic assays

**Fig. 4:**
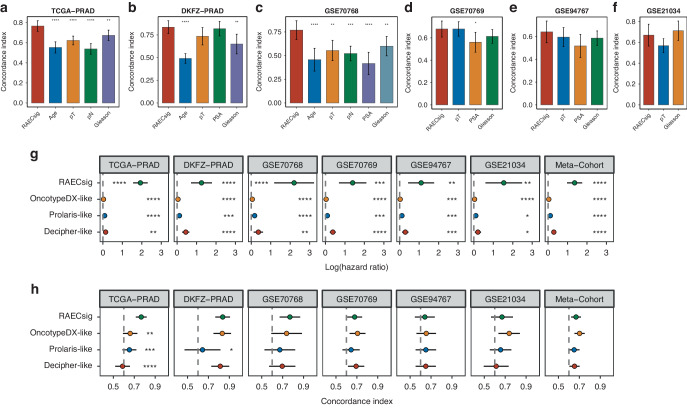
Comparison of RAECsig with clinical features and published models in predicting recurrence across PCa cohorts. **a**–**f** C-indices of the RAECsig, age, pathological tumor stage (pT), pathological lymph node stage (pN), prostate-specific antigen (PSA), and Gleason scores in different PCa cohorts, including TCGA-PRAD (**a**), DKFZ-PRAD (**b**), GSE70768 (**c**), GSE70769 (**d**), GSE94767 (**e**), and GSE21034 (**f**). **g** Univariate Cox regression analysis of RAECsig and published models across PCa cohorts. **h** C-indices of the RAECsig and published models in various PCa cohorts. Data in (**a**–**h**) are presented as mean ± 95% confidence interval. *P < 0.05; **P < 0.01; ***P < 0.001; ****P < 0.0001.

Clinicopathological variables such as age, Gleason score, and TNM stage are commonly used to assess PCa prognosis in clinical practice. We, therefore, used the C-index to compare the predictive power of RAECsig to those of common clinicopathological features across datasets (Table S9). The C-index [95% CI] of RAECsig was calculated with 0.767 [0.714–0.819] in TCGA-PRAD, 0.836 [0.765–0.907] in DKFZ-PRAD, 0.769 [0.671–0.868] in GSE70768, 0.679 [0.608-0.750] in GSE70769, 0.641 [0.546–0.736] in GSE94767, and 0.669 [0.564–0.774] in GSE21034 (Fig. 4a–f). Overall, RAECsig performed better than age, Gleason score, and TNM stage in TCGA-PRAD and GSE70768 but was statistically non-inferior in other datasets.

We also compared RAECsig with OncotypeDX, Prolaris, and Decipher. There were no overlapping genes between RAECsig and each of these 3 assays. We calculated OncotypeDX-like, Prolaris-like, and Decipher-like scores to samples in all datasets. Similar to the RAECsig scores, the OncotypexDX-like, Prolaris-like, and Decipher-like scores showed consistent positive associations with recurrence in all datasets (Fig. 4g). Notably, the univariate HRs of RAECsig were greater than those from the three assays in all datasets. Furthermore, we calculated C-indices for these signatures and found that RAECsig significantly outperformed the other three in TCGA-PRAD (OncotypeDX-like: 0.661 [0.592–0.729]; Prolaris-like: 0.654 [0.586–0.721]; Decipher-like: 0.588 [0.518–0.659]). In other datasets, RAECsig was comparable to the other three assays (Fig. 4h; Table S10). Additionally, we confirmed that RAECsig performed better than the endothelial signature in predicting PCa recurrence (Fig. S7). Altogether, RAECsig displayed robust predictive powers compared to clinical features and gene panels in different cohorts and, therefore, had better extrapolation potential.

### Key RAEC genes enhance angiogenic activity and PCa cell proliferation

**Fig. 5:**
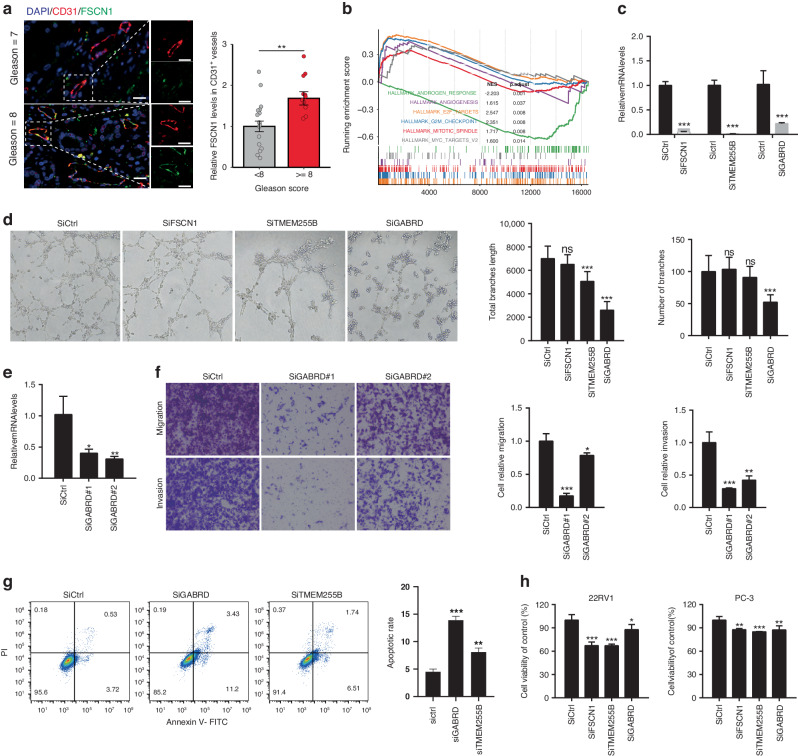
RAECs are associated with enhanced angiogenic activity and PCa tumor growth. **a** Representative immunostaining micrographs of FSCN1 and CD31 in human PCa samples with higher and lower Gleason scores. Nuclei were stained with DAPI. Images to the right are magnified boxed areas. Scale bars, 20 μm. Data are presented as means± s.e (*n* = 28, shown in the right panel). *P* value was calculated by the Wilcoxon rank sum test. **b** Gene set enrichment analysis revealed top dysregulated HALLMARK pathways in PCa patients with higher RAECsig scores. **c** Validation of RNAi-mediated knockdown of FSCN1, TMEM255B, and GABRD in HUVECs, as measured by real-time qPCR. Data are presented as means ± s.d (*n* = 3). Statistical significance was determined using two-tailed Student’s *t*-tests. **d** Tube formation of HUVECs was affected by silencing GABRD and TMEM255B. Data are presented as means ± s.d (*n* = 3, shown in right panels). **e** Knockdown of GABRD by 2 siRNAs in HUVEC, as measured by real-time qPCR. Data are presented as means ± s.d (*n* = 3). **f** GABRD knockdown inhibited HUVEC migration and invasion, as determined by the transwell assay. Data are denoted as means ± s.d (*n* = 3). **g** GABRD and TMEM255B knockdown induced HUVEC apoptosis. Data are denoted as means ± s.d (*n* = 3). **h** The number of PCa cells was decreased by conditioned media from HUVECs with the knockdown of FSCN1, TMEM255B, and GABRD, as determined by the CCK-8 assay. Data are shown as means ± s.d (*n* = 3). Statistical significance was determined by one-way ANOVA with Dunnett’s test for (**d**–**h**). *P < 0.05; **P < 0.01; ***P < 0.001.

Of the 12 upregulated RAEC-associated genes, *FSCN1* was ranked the top one by XGBoost (Fig. 2f), followed by *TMEM255B* and *GABRD*. Additionally, these 3 genes’ expression levels were significantly associated with PCa recurrence and higher Gleason scores (Fig. S6). We thus chose *FSCN1*, *TMEM255B*, and *GABRD* to evaluate the RAECsig in PCa progression. *FSCN1* is a marker of tip cells and was significantly upregulated in ECs from samples with higher Gleason scores (Fig. 5a). The expressions of TMEM255B and GABRD in ECs were also confirmed at the protein level (Fig. S7).

As a higher RAECsig score suggested more abundant RAECs, particularly more tip cells, we reasoned that PCa with higher RAECsig scores could manifest increased angiogenic activities to enhance tumor growth by supplying more oxygen and nutrients. Consistent with this idea, PCa with higher RAECsig scores were enriched in pathways of angiogenesis and cell cycle progression based on GSEA of consensus DEGs from the bulk RNA-seq datasets (Fig. 5b and S8; Table S11). Therefore, we assessed whether *FSCN1*, *TMEM255B*, and *GABRD* functionally enhance angiogenic activities. In HUVECs, silencing *GABRD* or *TMEM255B* significantly inhibited their tube formation, an indicator of angiogenesis (Fig. 5c, d). *GABRD* silencing was more potent than other genes in the tube formation assay. Consistently, *GABRD* silencing significantly reduced the migration and invasion of HUVECs (Fig. 5e, f). Also, we found that silencing *GABRD* or *TMEM255B* significantly induced apoptosis in HUVEC cells, which may also explain the dramatic reduction in their tube formation (Fig. 5g).

Accumulating evidence indicates that ECs can also regulate PCa progression via interacting with tumor cells [44–46]. We thus used collected media from HUVECs with the silencing of *FSCN1*, *TMEM255B*, and *GABRD* to treat human PCa cells and performed cell proliferation analysis. We found that silencing *FSCN1*, *TMEM255B*, or *GABRD* in HUVECs significantly inhibited the proliferation of PC-3 and 22Rv1 cells (Fig. 5h). These findings suggest that RAECs could also promote PCa progression by directly modulating tumor cell proliferation.

### The RAECsig also predicts castration resistance

**Fig. 6:**
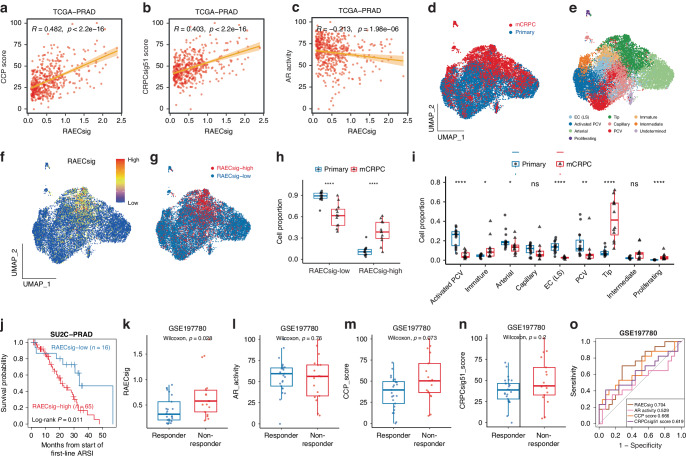
Higher RAECsig scores are associated with castration resistance in PCa. **a**–**c** Associations of higher RAECsig values with higher cell cycle progression (CCP) scores (**a**), higher values of a CRPC signature (CRPCsig51, **b**), and lower AR activities (**c**). The correlation coefficient R and corresponding *P* values were determined using Spearman’s rank correlation analysis. **d** UMAP plot of ECs collected from 4 PCa scRNA-seq datasets of primary PCa (blue) and metastatic CRPC (mCRPC, red). **e** UMAP plot of EC subtypes. EC (LS) represents cells with lower sequencing depth. **f** Featureplot showing RAECsig scores of ECs. RAECsig scores were calculated using scaled data as input for the RAECsig model. **g** UMAP plot of ECs stratified by RAECsig scores. The cut off RAECsig value for discretion was determined using the Youden index that predicts tip cells from RAECs with maximized sensitivity and specificity. **h**, **i** Boxplot showing proportions of RAECsig-high and RAECsig-low cells (**h**) and EC subtypes (**i**) in primary PCa and mCRPC. Dots represent the cell proportion from a sample. Statistical significance was determined using the Wilcoxon rank sum test. ns, not significant; *, *P* < 0.05; **, *P* < 0.01; ***, *P* < 0.001; ****, *P* < 0.0001. (**j**) Kaplan–Meier analysis showing overall survival with a first-line ARSI between RAECsig-high (red) and RAECsig-low (blue) mCRPC. **k**–**n** Boxplots showing baseline RAECsig (**k**), AR activity (**l**), CCP score (**m**), and CRPCsig51 score (**n**) between enzalutamide responders and non-responders in primary PCa. Statistical significance was determined using the Wilcoxon rank sum test. (**o**) ROC curves of RAECsig, AR activity, CCP score, and CRPCsig51 score in predicting the probability of primary resistance to enzalutamide in the GSE197780 dataset. CCP, cell cycle progression; AR, androgen receptor; PCV, postcapillary vein; LS, lower sequencing depth; mCRPC, metastatic castration-resistant prostate cancer; ARSI, AR-signaling inhibitors; ROC, receiver operating characteristic.

Cell cycle progression (CCP) is inversely correlated with AR activities and can predict primary abiraterone resistance in metastatic PCa [47]. We noticed that the downregulation of the androgen response pathway was accompanied by the enrichment of cell cycle-related pathways in RAECsig-high tumors (Fig. 5b). We thus investigated whether RAECs also play a role in castration resistance in PCa. We calculated the CCP scores, AR activities, and CRPCsig51 scores for tumor samples in the TCGA-PRAD, DKFZ-PRAD, and Meta-Cohort and found that higher RAECsig scores were significantly correlated with both CCP and CRPCsig51 scores while weakly correlated with negative androgen activities (Fig. 6a–c and Fig. S9A, B). To further test whether an increase in RAECs is associated with the development of castration resistance in PCa, we curated scRNA-seq data of ECs from both primary PCa and mCRPC based on the expression of *PECAM1* and *PROX1* (Fig. S10A–M). After removing samples with less than 50 ECs (Fig. S10N) and correcting batch effects (Fig. S11A–D), a total of 16645 ECs from 28 samples were used to generate a transcriptional atlas of ECs across PCa stages (Fig. 6d). All EC subtypes described above were identified based on canonical markers (Fig. 6e and S10F–11E).

We then calculated the RAECsig score for each subtype of ECs. Again, ECs with higher RAECsig scores were mostly tip cells (Fig. 6f). RAECsig-high ECs were significantly enriched in mCRPC (Fig. 6g) whereas RAECsig-low ECs were more enriched in primary tumors (Fig. 6h). Consistent with the enrichment of tip and immature cells in RAECs, both tip and immature cells were also enriched in mCRPC (Fig. 6i). In addition, mCRPCs with higher RAECsig scores had significantly worse prognoses after treatments with first-line AR-signaling inhibitors (ARSI) (Fig. 6j).

We also tested whether higher RAECsig scores can predict primary resistance to ARSI in primary PCa using the GSE197780 dataset, in which patients underwent bulk RNA sequencing before 3 months of neoadjuvant enzalutamide treatment [35]. Non-responders had significantly higher RAECsig scores than responders in these primary tumors (Fig. 6k). However, we did not detect significant differences in the primary tumors’ AR activities, CCP scores, and CRPCsig51 scores (Fig. 6l–n). ROC analysis showed that RAECsig could markedly discriminate non-responders from responders of enzalutamide (AUC = 0.704; Fig. 6o). These findings suggest that RAECsig can identify patients who may not respond to ADT.

## Discussion

In this study, we explored whether and how endothelial cells play a role in the progression of PCa using publically available scRNA-seq and bulk transcriptomic data. Our analyzes demonstrated that a subset of ECs (i.e. RAECs) is associated with PCa recurrence and RAECs are characteristic of tip and immature ECs with more active angiogenic activities. A robust gene signature was developed for RAECs, which predicts PCa recurrence and castration resistance. A schematic summarizing the flowchart and key findings of this study was shown in Fig. S12.

It has been demonstrated that, as a key component of the TME, tumor endothelial cells are heterogeneous, impact tumor progression, and are potential therapeutic targets in some types of tumors [16, 48, 49]. As revealed by our analyzes in this study, such heterogeneous TECs not only exist in PCa, but their higher abundances in primary tumors were also associated with shorter disease-free survival (Fig. 1a, S1). Consistent with TECs in other types of tumors, there were also multiple subpopulations of TECs in PCa, as revealed by the annotation of a recently published scRNA-seq study [17] (Figs. 1b, c). Although this study highlights the role of TECs in PCa progression, the prognostic and therapeutic implications of TEC heterogeneity in PCa remain elusive. Our study identified a subpopulation, which was significantly associated with worse disease-free survival in PCa (Fig. 1d). This subpopulation, named RAECs for recurrence-associated ECs, was defined by the expression of 54 RAEC-specific genes (Figs. 1f, g, Table S6). Higher expression scores of these 54 genes predicted worse disease-free survival in PCa patients (Fig. 1h).

Interestingly, RAECs possess the features of tip cells and immature ECs, which play pivotal roles in angiogenesis and could thus be responsible for the association of RAECs with worse patient survival. Upon VEGF induction, quiescent ECs acquire the phenotype of tip cells [50], which are characterized by long filopodia and motile behaviors, and initiate and guide vessel sprouting during angiogenesis [50]. One primary marker of tip cells, *FSCN1* [18, 51], is highly expressed in RAECs (Fig. 2h). FSCN1 expression was also elevated in ECs from PCa with higher Gleason scores, further supporting the association of RAECs with PCa progression. These findings are consistent with a recent scRNA-seq study in which both tip and immature cells were increased in lung cancers, and their signatures were significantly associated with poorer survival in patients with lung cancer [18]. We noticed that tip cells from RAECs and a breach EC phenotype in lung cancer shared similar expression patterns, including the upregulation of tip cell markers and collagen remodeling markers (*COL4A1*, *COL4A2*) (Fig. S2G) [18]. It is thus likely that the EC phenotypes involved in tumor progression are shared by different types of cancers and therefore important across tumor types.

Expression profiling of RAECs also supports the notion that RAECs possess higher angiogenic activities. For example, RAECs expressed higher levels of VEGFRs and were enriched in pathways of angiogenesis, migratory, and ECM modeling (Fig. S2H, I). In addition, transcription factor ZEB1 was upregulated in RAECs (Figs. S2J, S2G), and elevated ZEB1 expression in ECs promotes angiogenesis, growth, and metastasis of lung cancer cells by increasing TGF-β levels [52].

Some of the 18 genes in the RAECsig have been well implicated in tumor angiogenesis. For example, FSCN1 is an actin cross-linker important for tip cell competitivity [18], and germline mutations of DOCK6, an endothelial RhoGEF, is associated with diffuse angiopathy with incomplete microvascularization [53, 54]. ESM1 is a dermatan sulfate proteoglycan that modulates vascular permeability and angiogenesis [55], APLN and its only known receptor APLNR are essential for angiogenic sprouting and high glycolytic rate in ECs [56], and endothelial CAII expression is associated with a high-grade form of glial tumors with worse prognosis [57] likely by regulating TEC survival under lactic acidosis [58].

*GABRD* and *TMEM255B* were among the top 5 of the 18 RAECsig genes but have not been functionally characterized in ECs. Our findings suggest that both *GABRD* and *TMEM255B* are important for angiogenesis, albeit to varying degrees (Fig. 5d). Conditioned medium from endothelial cells with these two genes’ silencing also affected PCa cells’ proliferation (Fig. 5h). GABRD is the delta subunit of the Gamma-aminobutyric acid type A (GABA_A_) receptor. Deletion of the beta-3 subunit of the GABA_A_ receptor in ECs leads to a decrease in calcium influx and inhibition of both EC proliferation and angiogenesis in the forebrain of mice [59]. However, the mechanisms by which GABRD and TMEM255B mediate tumor angiogenesis and proliferation remain to be elucidated.

One surprising finding was that the RAECsig also predicted therapeutic resistance to ARSI therapy. Higher RAECsig scores were significantly correlated with cell cycle progression, CRPCsig51 scores, and reduced androgen responses, which are characteristic of castration resistance (Fig. 6). In addition, ECs with higher RAECsig scores were significantly enriched in CRPCs than primary tumors (Fig. 6h), CRPCs with higher RAECsig scores had significantly worse prognoses after treatments with ARSI (Fig. 6J), and primary tumors with higher RAECsig scores were less responsive to ARSI (Fig. 6k). The finding of RAECsig-high ECs being more prevalent in CRPC than in primary PCa is consistent with that of a previous study, where a population of ECs with more active communication with tumor cells is enriched in CRPCs [17]. One of the genes overexpressed in this group of ECs, THY1, is upregulated in CRPC [17] and was among the upregulated genes in RAECs (Table S5).

ADT and ARSIs are commonly used to treat advanced PCa and are recommended as neoadjuvant therapy for high-risk localized PCa [60–62]. The RAECsig should thus be useful for identifying patients who may benefit from hormonal therapy in the clinical management of PCa.

Under the stress of androgen deprivation, prostate vasculature rapidly involutes but later recovers by upregulating key molecules such as VEGFA, VEGFR2, and basic fibroblast growth factor [63]. Therefore, it remains to be determined whether the expansion of ECs with higher RAECsig scores represents a mechanism for castration resistance or simply a vascular response to ADT or ARSI therapy. Nonetheless, the association of higher RAECsig scores with ARSI resistance in both CRPC and primary hormone-naïve tumors suggests the increase in RAECs is a novel mechanism for castration resistance. This possibility is supported by the observations that perturbing RAEC-specific genes in ECs affected PCa cell proliferation (Fig. 5g). Further investigation is needed to clarify this issue.

In summary, this study demonstrates that a subset of ECs in PCa is associated with recurrence and possesses the characteristics of tip and immature cells. These RAECs can be defined by an 18-gene signature named RAECsig in bulk transcriptomic data. Higher RAECsig predicts tumor recurrence independent of other clinicopathological parameters, such as the Gleason score and the TNM stage. Many of the 18 RAECsig genes functionally modulate ECs and cancer cells’ proliferation. Our findings suggest that the RAECsig is useful for discriminating patients who benefit from ARSI therapy from those who do not, and that expansion of RAECs may represent a novel mechanism for castration resistance in PCa.

### Supplementary information

Supplementary Figures

Supplementary Tables

Supplementary Table11

## Data Availability

The datasets used in this study could be obtained through GEO (https://www.ncbi.nlm.nih.gov/geo/), cBioPortal (https://www.cbioportal.org/), XENA (https://xena.ucsc.edu/) and the corresponding authors, as detailed in Table S1.
