# Supplementary material for: An 18-gene signature of recurrence-associated endothelial cells predicts tumor progression and castration resistance in prostate cancer
Source: Br J Cancer. 2024 Jul 12;131(5):870–82. doi: 10.1038/s41416-024-02761-0 (PMC11369112; doi:10.1038/s41416-024-02761-0)
Supplement: Supplementary file 1 — Supplementary Figures [file 41416_2024_2761_MOESM1_ESM.docx]

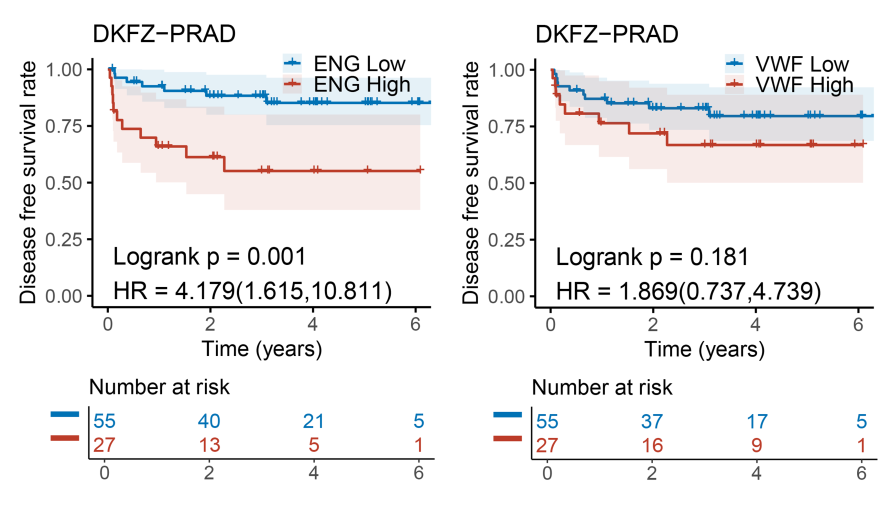


Figure S1. Kaplan-Meier curves of disease-free survival in *ENG*-high and *ENG*-low samples (left) and *VWF*-high and *VWF*-low samples (right) in DKFZ-PRAD. *P* values were derived from the log-rank tests. The Hazard ratio (HR) and 95% confidence interval (CI) were computed using the univariate Cox regression analysis. *PECAM1* is not identified in DKFZ-PRAD.


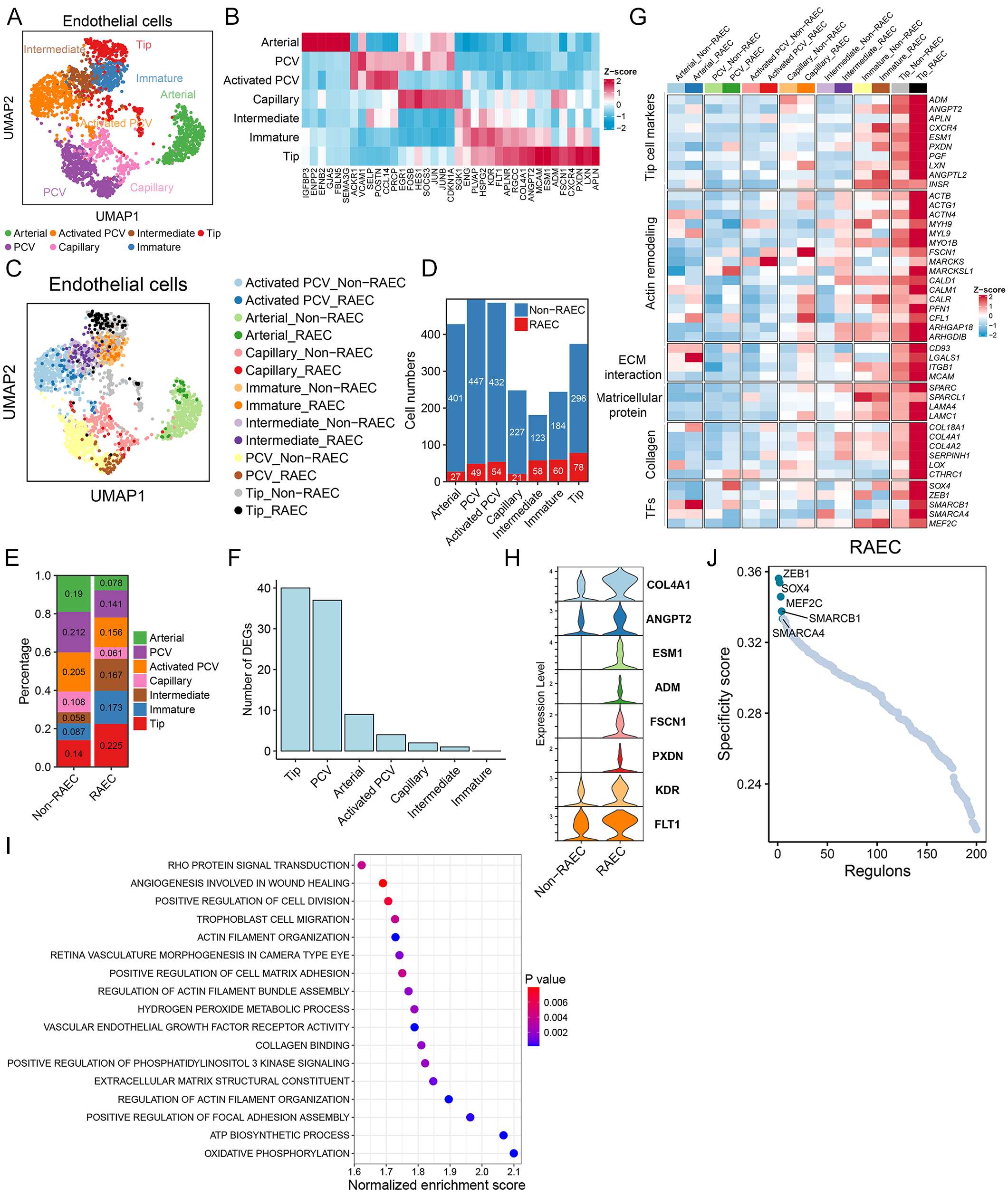


Figure S2. RAECs exhibit signatures of differentiated tip cells and active angiogenesis. (A) UMAP plot of EC subtypes in the Chen dataset. (B) Heat map showing relative mRNA levels of marker genes for various EC subtypes. (C) UMAP plot of EC subtypes stratified by RAECs and non-RAECs. (D) Numbers of RAECs and non-RAECs in each EC subtype. Red and blue indicate RAECs and non-RAECs, respectively. (E) Percentages of EC subtypes in RAECs and non-RAECs. (F) Numbers of differentially expressed genes (DEGs) between RAECs and non-RAECs in each EC subtype. (G) Heat map showing relative mRNA levels of marker genes related to tip cells, actin modeling, extracellular matrix (ECM) interaction, matricellular proteins, collagen, and transcription factors (TFs) across EC subtypes stratified by RAECs and non-RAECs. (H) Violin plots demonstrating expression of marker genes related to tip cell and angiogenesis in RAECs and non-RAECs. (I) Results of gene set enrichment analysis (GSEA) between RAECs and non-RAECs. (J) Regulons that may promote RAEC development, as revealed by the single-cell regulatory network inference and clustering (SCENIC) analysis. PCV, postcapillary vein; RAECs, recurrence-associated endothelial cells.


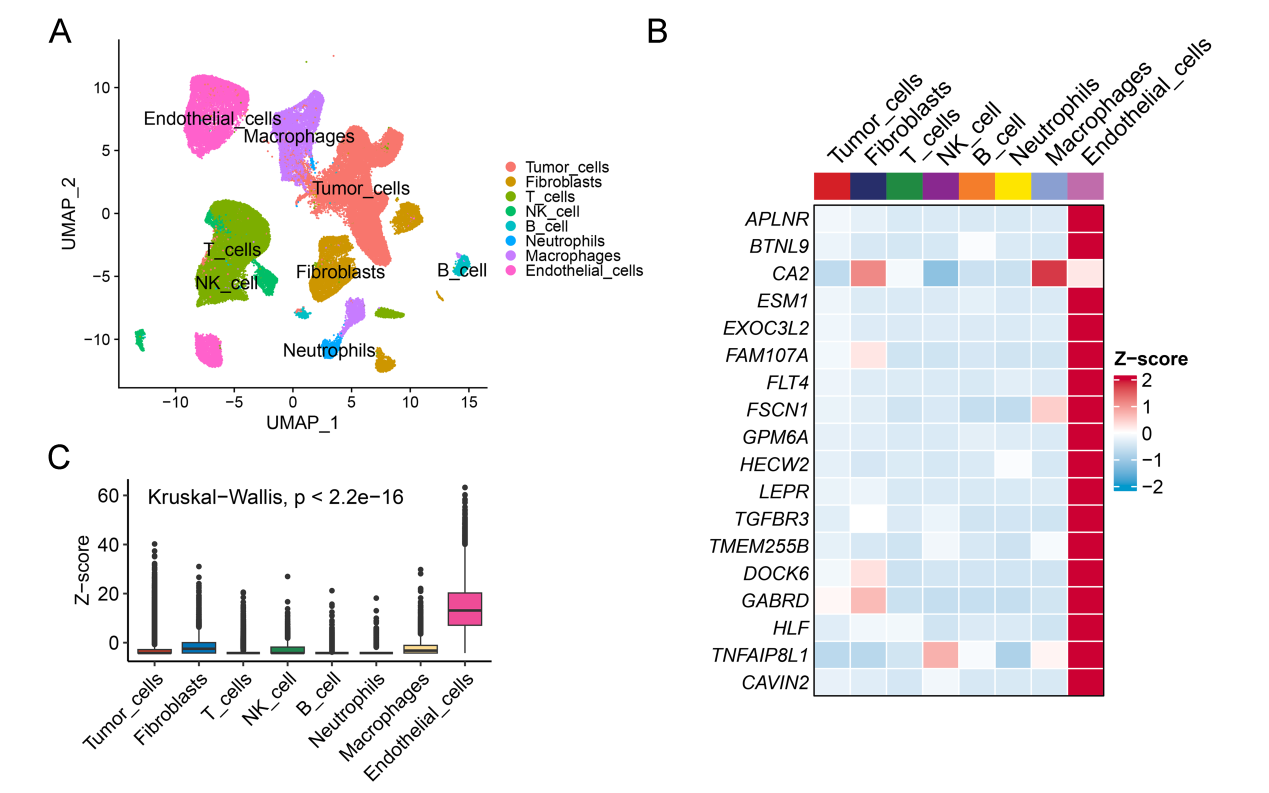


Figure S3. The 18 prognostic RAEC-related genes were independently validated in another primary PCa scRNA-seq dataset. (A) UMAP plot of major cell types in the Ge dataset. Colors represent cell types. Dots represent cells. (B) Heat map showing mRNA relative expression of the 18 genes across major cell types. (C) Boxplot showing scores of major cell types that were derived from summing the z-scores of 18 genes.


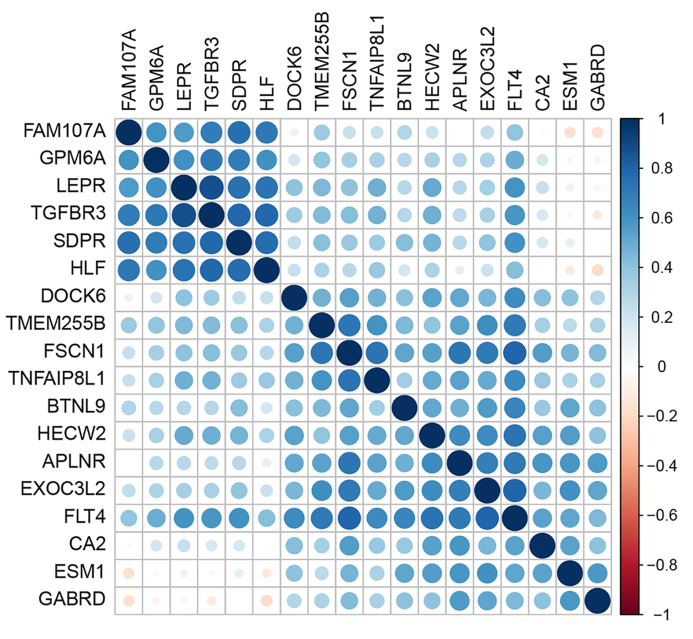


Figure S4. Expression patterns of the 18 prognostic RAEC-related genes in the TCGA-PRAD cohort. Hierarchical clustering was performed using Spearman’s correlation coefficients. RAECs, recurrence-associated endothelial cells.


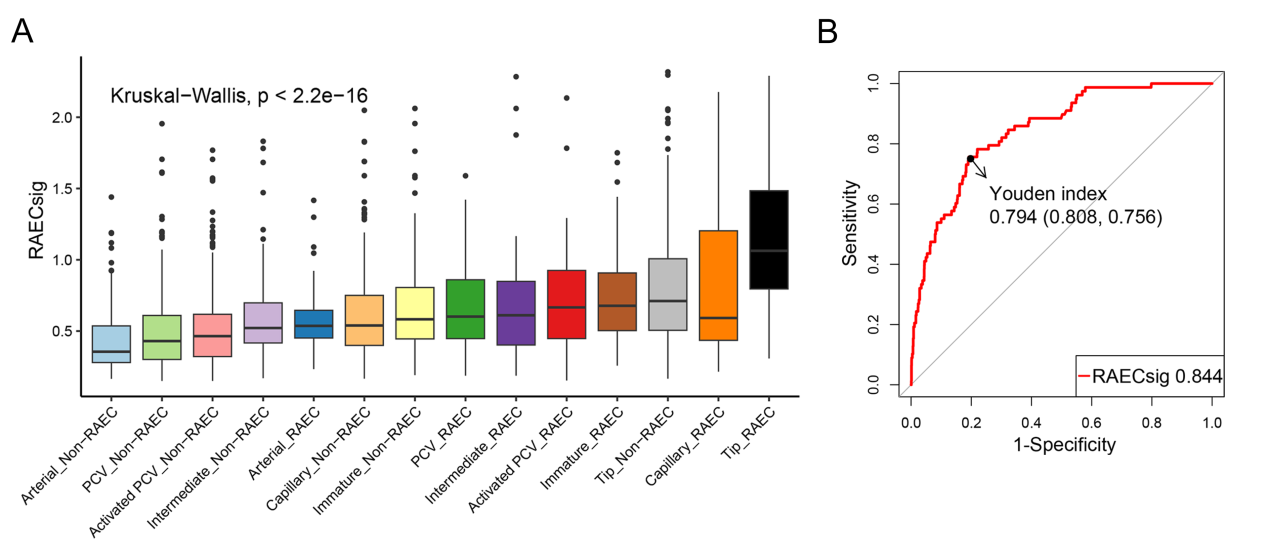


Figure S5. The RAECsig mainly indicates tip cells in RAECs. (A) Boxplot showing RAECsig scores of different EC subtypes stratified by RAECs and non-RAECs in the Chen dataset. (B) The ROC curve of RAECsig in predicting the probability of tip cells of RAECs from remaining ECs. The black dot indicates the Youden index with a RAECsig value of 0.794. RAECsig, recurrence-associated endothelial cell signature; PCV, postcapillary vein.


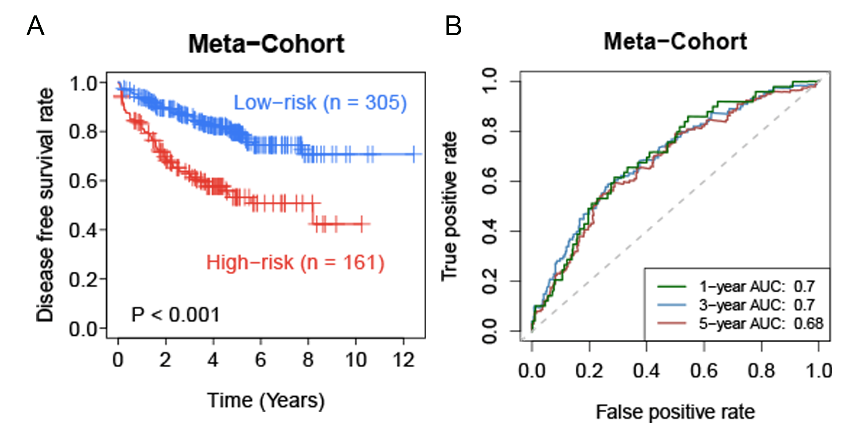


Figure S6. The Kaplan-Meier (A) and ROC (B) curves of RAECsig in the Meta-cohort. RAECsig, recurrence-associated endothelial cell signature; ROC, receiver operating characteristic; AUC, area under the curve.


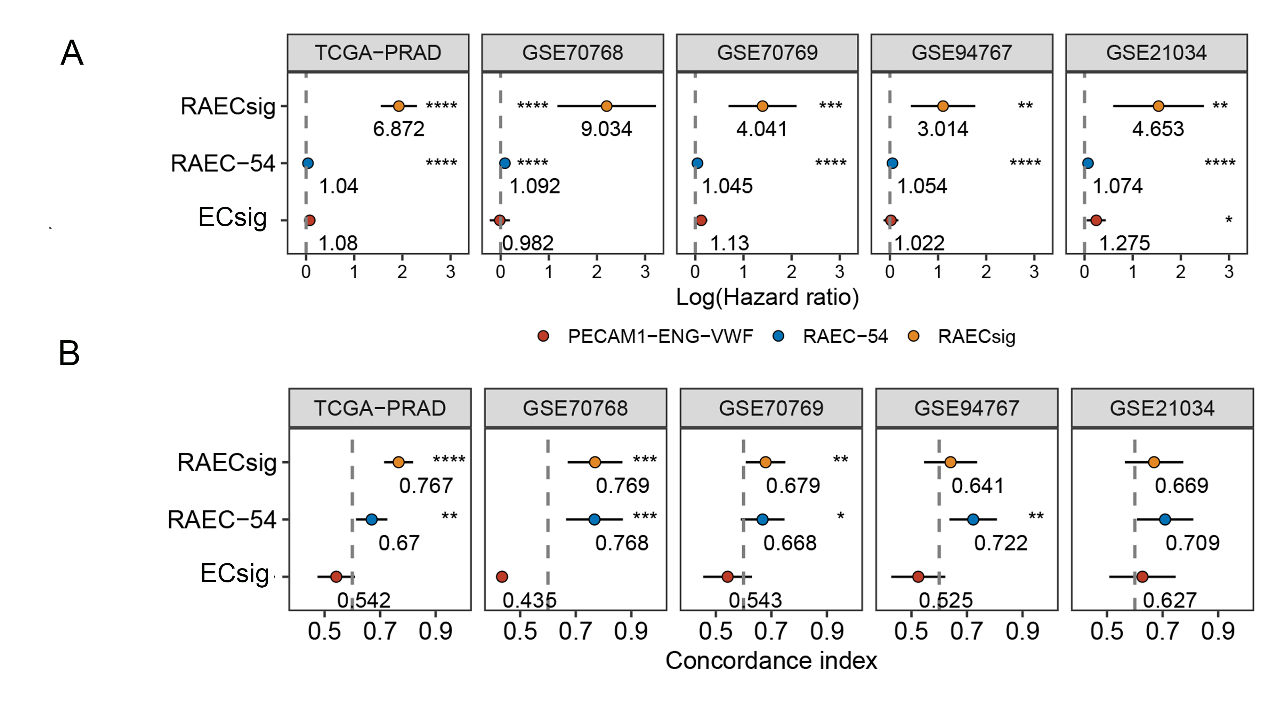


Figure S7. Comparison of the endothelial signature (ECsig) with RAECsig and RAEC-54 genes in predicting recurrence across PCa cohorts. (A) Univariate Cox regression and (B) C-indices of ECsig, RAEC-54 genes, and RAECsig across PCa cohorts. Data in (A-B) are presented as mean ± 95% confidence interval. ECsig consists of *PECAM1*, *ENG1*, and *VWF*. *, *P* < 0.05; **, *P* < 0.01; ***, *P* < 0.001; ****, *P* < 0.0001. RAECsig, recurrence-associated endothelial cell signature; PCa. prostate cancer.


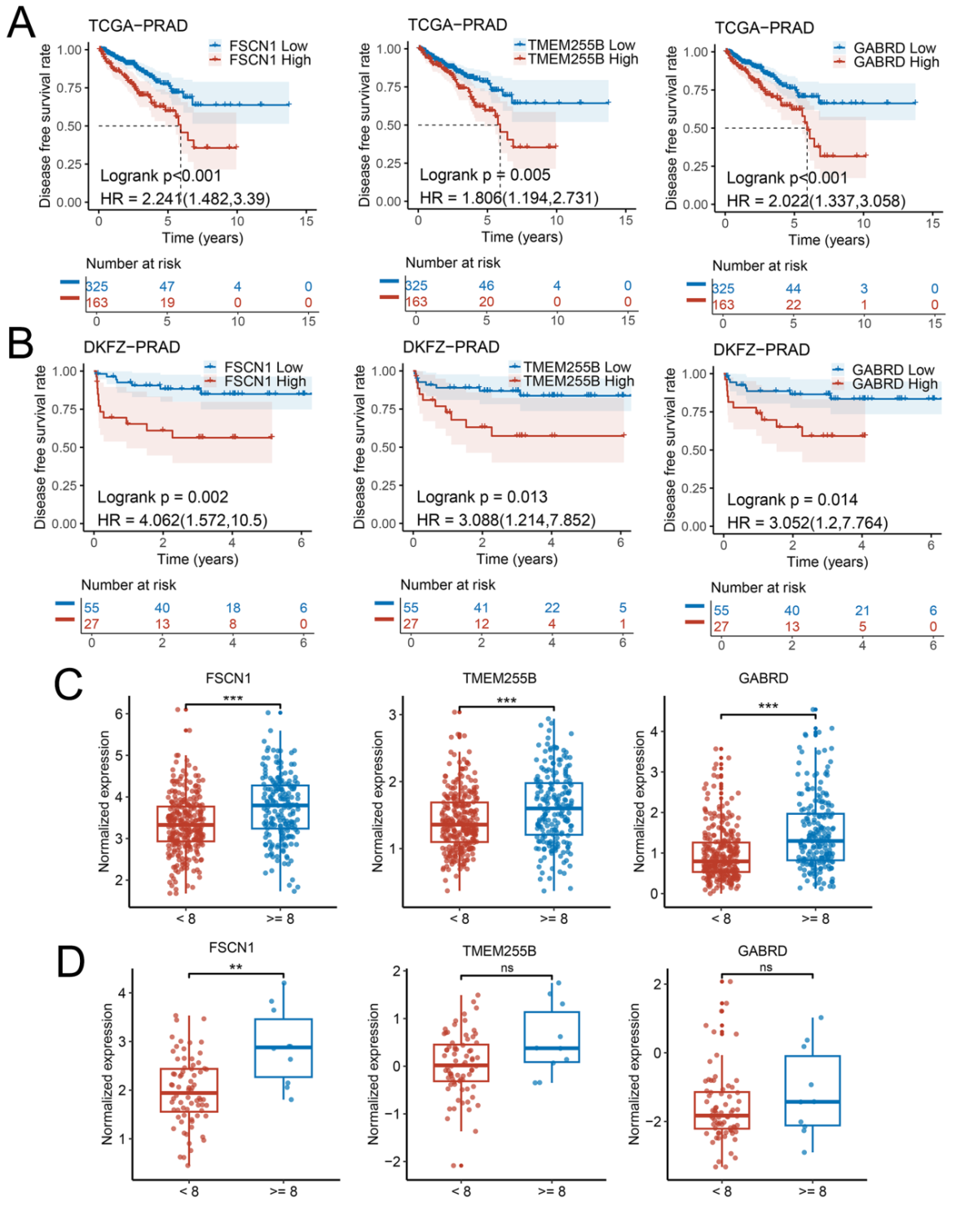


Figure S8. Higher mRNA levels of *FSCN1*, *TMEM255B*, and *GABRD* are associated with PCa progression. (A, B) Kaplan–Meier analysis showing an association of worse disease-free survival with higher mRNA levels of *FSCN1*, *TMEM255B*, and *GABRD* in the TCGA-PRAD (A) and DKFZ-PRAD (B) cohorts. *P* values were determined using the log-rank test. The hazard ratio (HR) and 95% confidence interval were from the univariate Cox regression analysis. (C, D) Boxplots showing increased mRNA levels of *FSCN1*, *TMEM255B*, and *GABRD* in PCa with Gleason scores >= 8 compared to PCa with Gleason scores < 8 from the TCGA-PRAD (C) and DKFZ-PRAD (D) cohorts. Statistical significance was determined using the Wilcoxon rank sum test. ns, not significant; *, *P* < 0.05; **, *P* < 0.01; ***, *P* < 0.001.


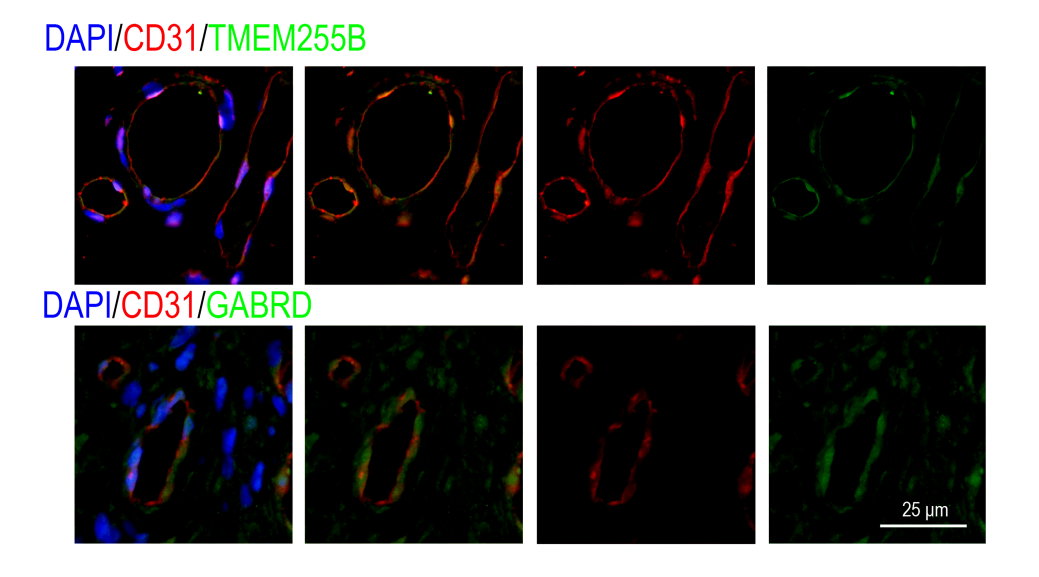


Figure S9. Representative immunostaining micrographs of TMEM255B, GABRD, and CD31 expression in clinical PCa samples. Scale bars, 25 μm.


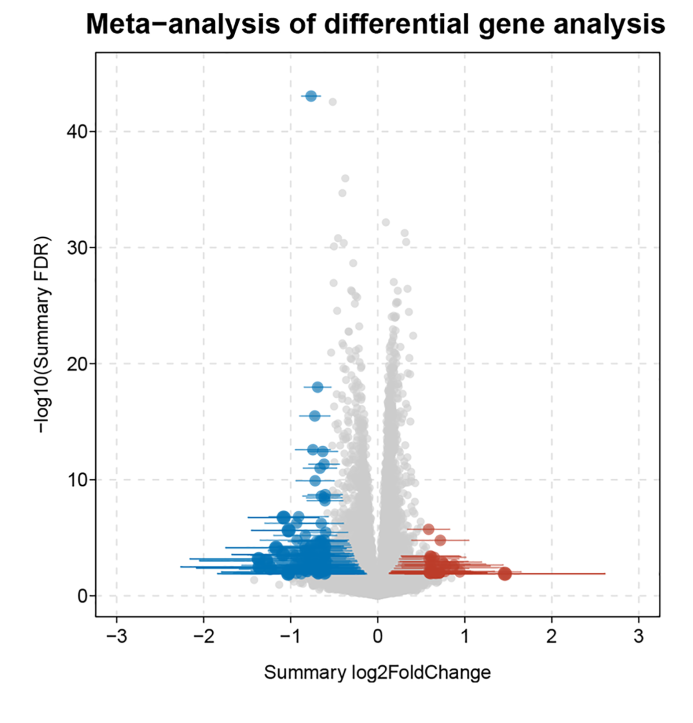


Figure S10. The volcano plot of differential gene expression analysis based on the meta-analysis of the TCGA-PRAD, DKFZ-PRAD, and the Meta-Cohort. Red dots represent genes with log2(Summary fold change) > 0.3 and adjusted *P* < 0.05, while blue dots denote genes with log2(Summary fold change) < -0.3 and adjusted *P* < 0.05. The horizontal lines crossing dots indicate the corresponding 95% confidence intervals, which, together with the Summary fold change, was derived from the meta-analysis of differential gene analysis.


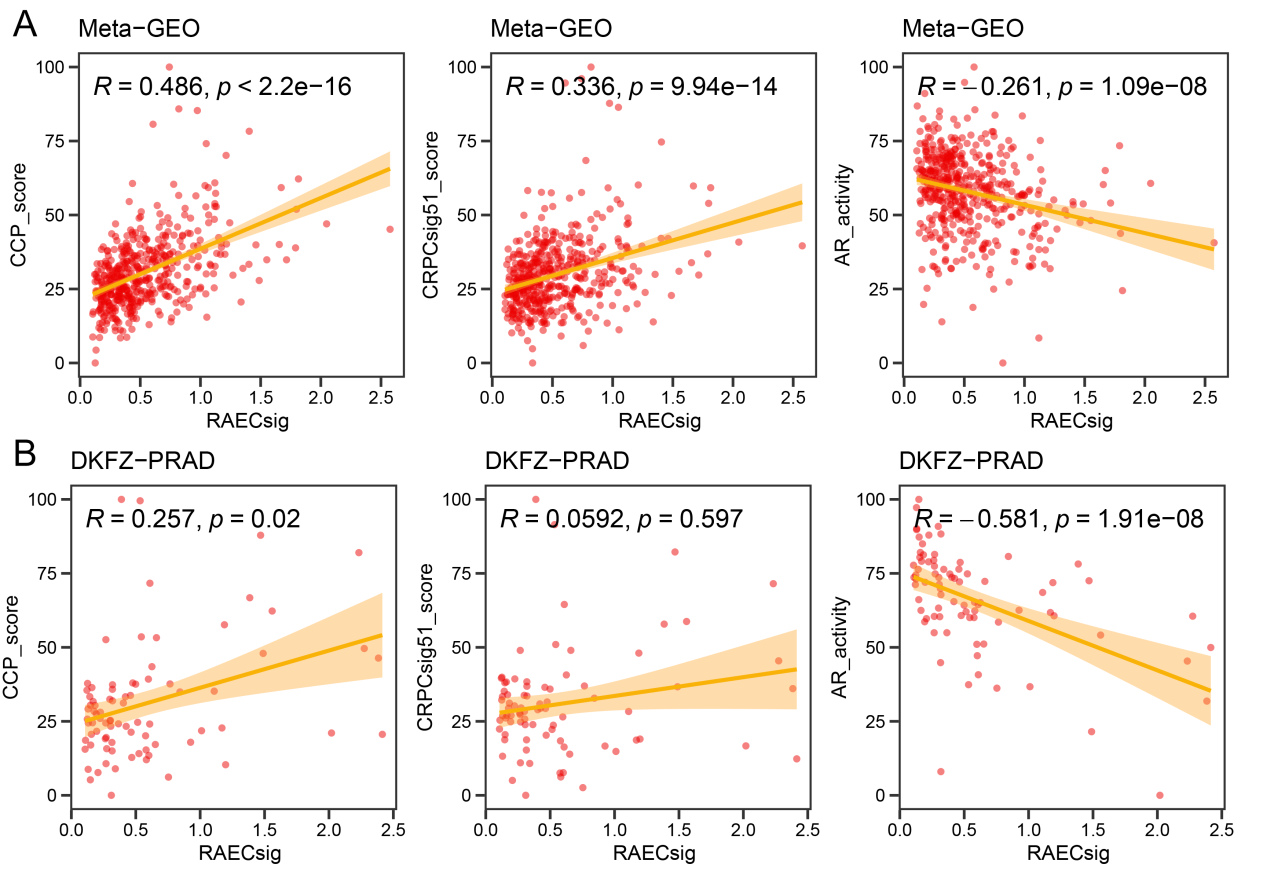


Figure S11. Higher RAECsig scores are associated with higher CCP scores, higher CRPCsig51 scores, and lower AR activities, as determined in the Meta-Cohort and DKFZ-PRAD cohort. The correlation coefficient R and corresponding *P* values were determined using Spearman’s rank correlation analysis. RAECsig, recurrence-associated endothelial cell signature; CCP, Cell cycle progression; AR, androgen receptor.


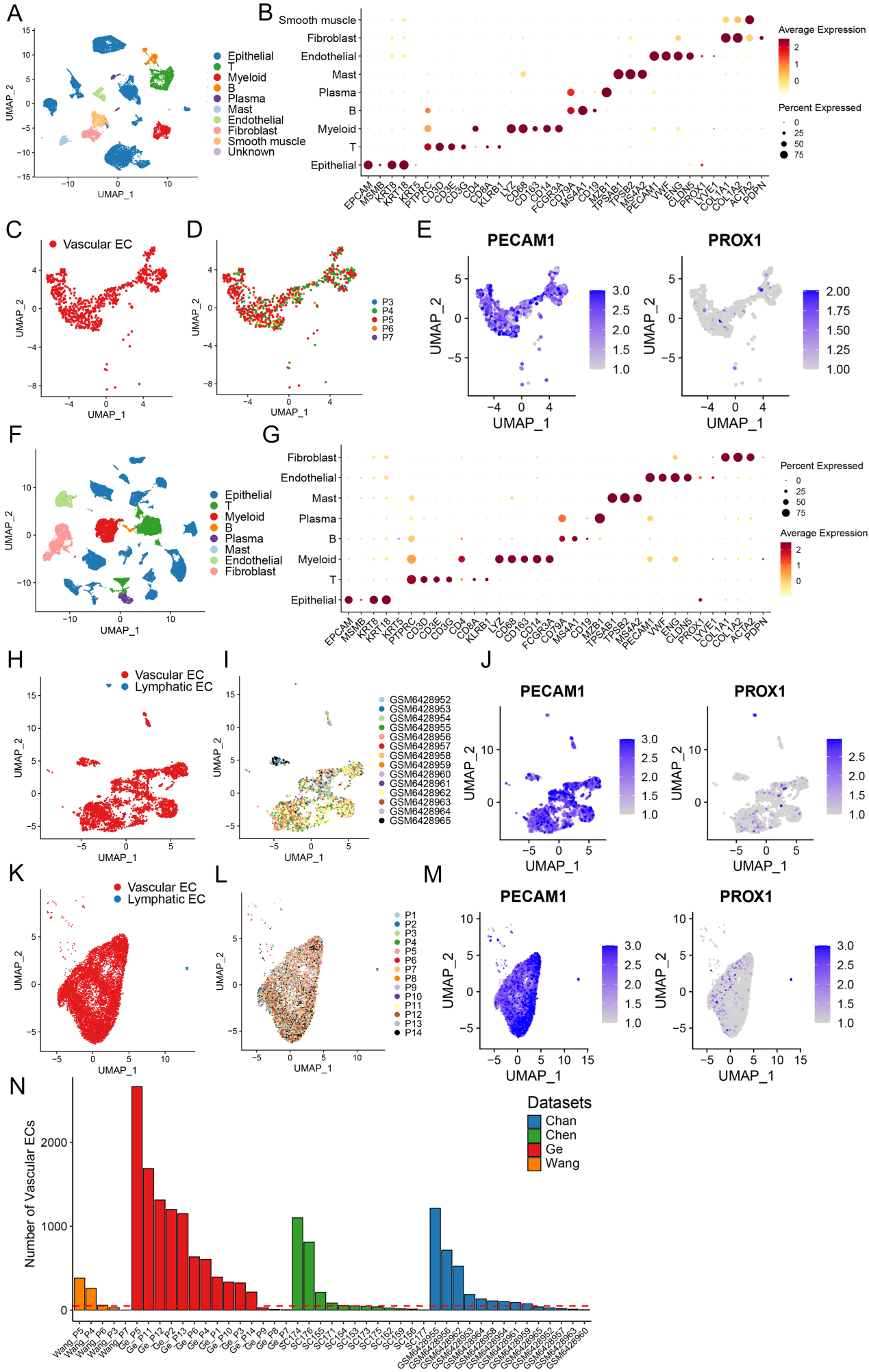


Figure S12. Preprocessing and cell annotation of curated prostate cancer scRNA-seq datasets. (A) UMAP plot of major cell types in mCRPC from the Wang dataset. Colors indicate different cell subtypes. (B) Dotplot showing relative mRNA expression levels of canonical markers across major cell types. The size of a dot represents the percentage of cells expressing a gene. The color gradient reflects expression levels. (C) UMAP plot of ECs. (D) UMAP plot of ECs colored by different samples (n = 5). Samples that were not CRPC (n = 2) were filtered before data processing. (E) Feature plot showing expression of *PECAM1* and *PROX1* in ECs. (F) UMAP plot of major cell types in mCRPC from the Chan dataset. Colors indicate different cell subtypes. (G) Dotplot showing relative expression levels of canonical markers across major cell types. (H) UMAP plot of ECs. (I) UMAP plot of ECs colored by different samples (n = 14). (J) Feature plot showing expression of *PECAM1* and *PROX1* in ECs. (K) UMAP plot of ECs in primary PCa from the Ge dataset (Figure S2). (L) UMAP plot of ECs colored by different samples (n = 14). (M) Feature plot showing expression of *PECAM1* and *PROX1* in ECs. (N) Barplot showing numbers of vascular ECs in samples (n = 41) from the included 4 datasets. The red horizontal line indicates a cell number of 50. Samples with > 50 vascular ECs (n=28) were retained for downstream analyses. mCRPC, metastatic castration-resistant prostate cancer; ECs, endothelial cells


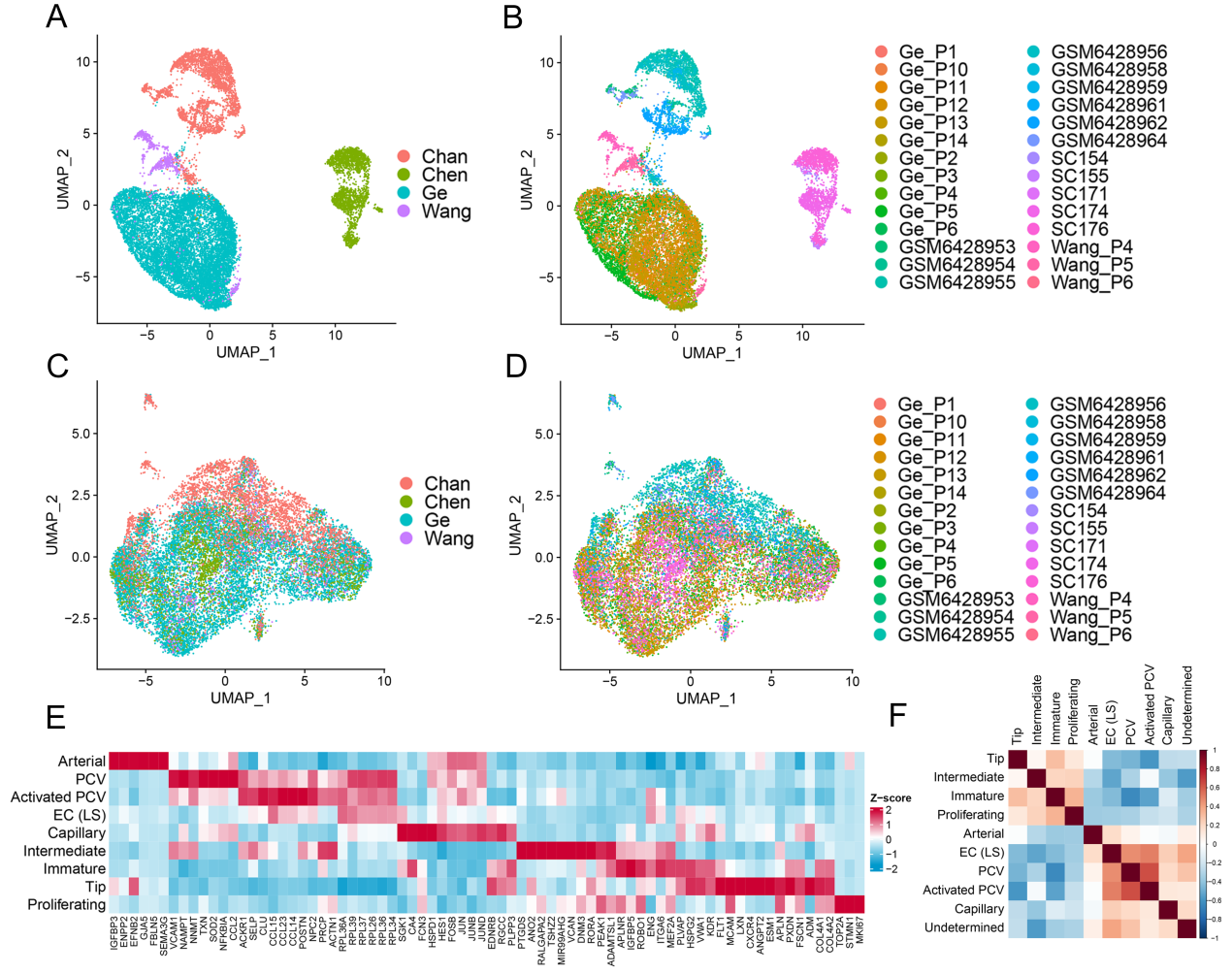


Figure S13. Construction of the endothelial cell transcriptional atlas across PCa stages. (A-D) UMAP plot of ECs colored by different datasets (n = 4) (A, C) and different samples (n = 28) (B, D) before (A, B) and after (C, D) correcting batch effect using the Harmony algorithm. (E) Heat map showing relative mRNA expression levels of genes across EC subtypes. (F) Correlation heatmap of highly variable genes in EC subtypes. EC, endothelial cell; PCV, postcapillary vein; LS, lower sequencing depth.


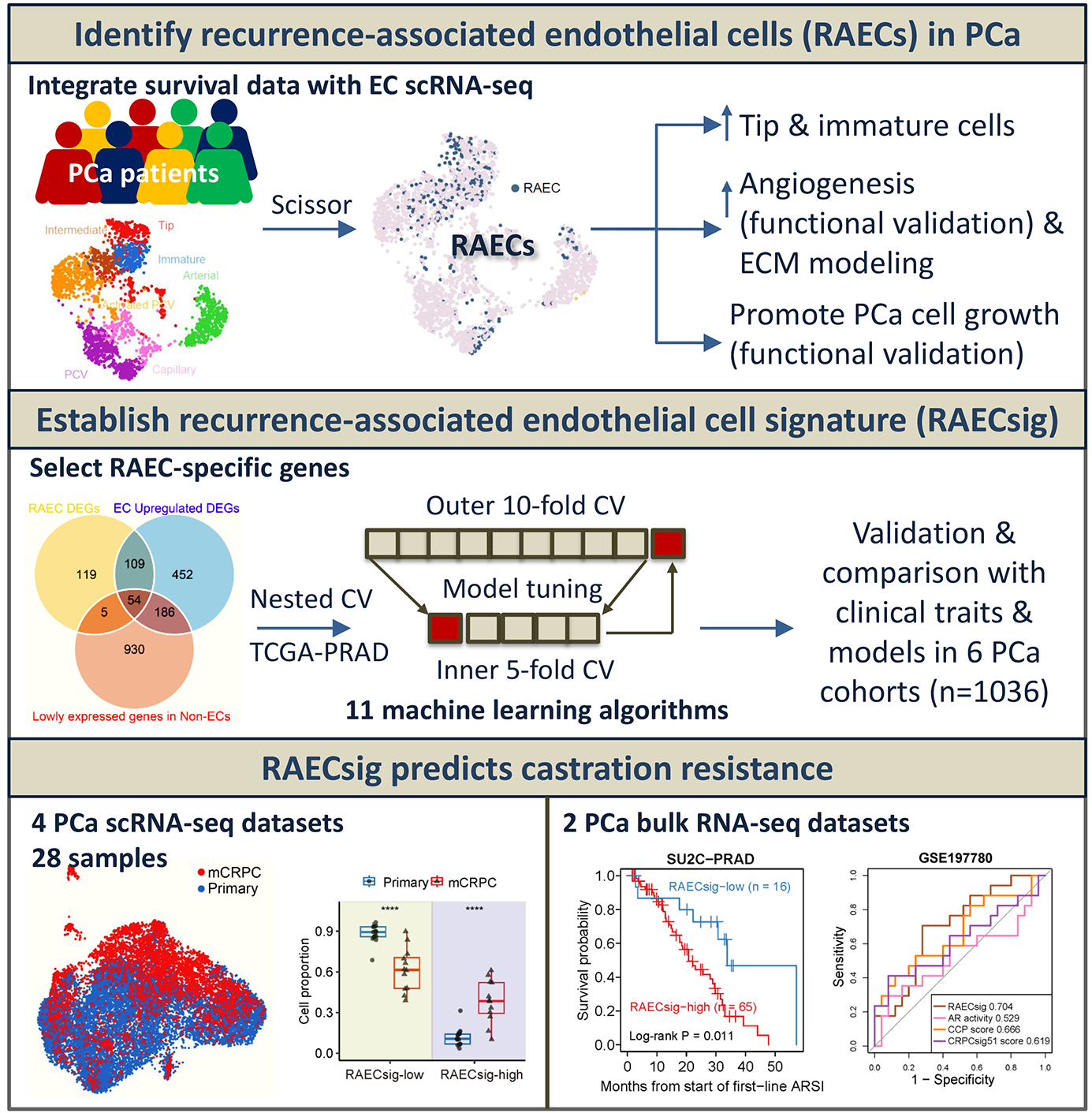


Figure S14. A schematic summarizing the flowchart and key findings of this study. ECM, extracellular matrix; CV, cross-validation.
