# Supplementary material for: An 18-gene signature of recurrence-associated endothelial cells predicts tumor progression and castration resistance in prostate cancer
Source: Br J Cancer. 2024 Jul 12;131(5):870–82. doi: 10.1038/s41416-024-02761-0 (PMC11369112; doi:10.1038/s41416-024-02761-0)
Supplement: Supplementary file 2 — Supplementary Tables [file 41416_2024_2761_MOESM2_ESM.docx]

| **Table S1 Description of human datasets used in this study** | | | | | |
| --- | --- | --- | --- | --- | --- |
| **Name** | **Incusion criteria** | **Cases** | **Platform** | **Source** | **PMID** |
| TCGA-PRAD | Primary prostate tumors with available survival data | 488 | Illumina HiSeq 2000 | <https://xena.ucsc.edu/> | 26544944 |
| DKFZ-PRAD | Primary prostate tumors with available survival data | 82 | Illumina HiSeq 2000 | <https://www.cbioportal.org/> | 20579941 |
| GSE70768 | Primary prostate tumors with available survival data | 111 | Illumina HumanHT-12 V4.0 expression beadchip | <https://www.ncbi.nlm.nih.gov/geo/> | 26501111 |
| GSE70769 | Primary prostate tumors with available survival data | 92 | Illumina HumanHT-12 V4.0 expression beadchip | <https://www.ncbi.nlm.nih.gov/geo/> | 26501111 |
| GSE21034 | Primary prostate tumors with available survival data | 131 | Affymetrix Human Exon 1.0 ST Array | <https://www.ncbi.nlm.nih.gov/geo/> | 20579941 |
| GSE94767 | Primary prostate tumors with available survival data | 132 | Affymetrix Human Exon 1.0 ST Array | <https://www.ncbi.nlm.nih.gov/geo/> | 28753852 |
| SU2C-PRAD | mCRPC with available survival data | 81 | Illumina HiSeq 2500 | <https://www.cbioportal.org/> | 31061129 |
| GSE197780 | Primary prostate tumors with neoadjuvant enzalutamide treatment | 42 | Illumina HiSeq 2500 | <https://www.ncbi.nlm.nih.gov/geo/> | 35754340 |
| Chen | Primary hormone-naïve prostate tumors | 12 | 10x Genomics | <https://www.ncbi.nlm.nih.gov/geo/> | 33420488 |
| Ge | Primary hormone-naïve prostate tumors | 14 | 10x Genomics | Request from the Author | 35322584 |
| Wang | mCRPC | 5 | 10x Genomics | Request from the Author | 35789834 |
| Chan | mCRPC | 14 | 10x Genomics | <https://www.ncbi.nlm.nih.gov/geo/> | 35981096 |

| **Table S2 Clinicopathological characteristics of prostate cancer cohorts** | | | |
| --- | --- | --- | --- |
| Clinicopathological characteristics of the TCGA-PRAD cohort | | | |
|  | RAECsig-low (N=300) | RAECsig-high (N=188) | P |
| Age: |  |  | 0.035 |
| <=60 | 147 (49.0%) | 73 (38.8%) |  |
| >60 | 153 (51.0%) | 115 (61.2%) |  |
| Relapse: |  |  | <0.001 |
| No | 274 (91.3%) | 124 (66.0%) |  |
| Yes | 26 (8.67%) | 64 (34.0%) |  |
| pT: |  |  | <0.001 |
| <=2 | 148 (49.3%) | 36 (19.1%) |  |
| >2 | 148 (49.3%) | 150 (79.8%) |  |
| NA | 4 (1.33%) | 2 (1.06%) |  |
| pN: |  |  | <0.001 |
| 0 | 225 (75.0%) | 114 (60.6%) |  |
| 1 | 20 (6.67%) | 57 (30.3%) |  |
| Unknown | 55 (18.3%) | 17 (9.04%) |  |
| Gleason score: |  |  | <0.001 |
| <8 | 229 (76.3%) | 59 (31.4%) |  |
| >=8 | 71 (23.7%) | 129 (68.6%) |  |

| Clinicopathological characteristics of the DKFZ-PRAD cohort | | | |
| --- | --- | --- | --- |
|  | RAECsig-low (N=56) | RAECsig-high (N=26) | P |
| Age: |  |  | 1.000 |
| <= 40 | 3 (5.36%) | 1 (3.85%) |  |
| >40 | 53 (94.6%) | 25 (96.2%) |  |
| Relapse: |  |  | <0.001 |
| No | 51 (91.1%) | 13 (50.0%) |  |
| Yes | 5 (8.93%) | 13 (50.0%) |  |
| pT: |  |  | <0.001 |
| <= 2 | 46 (82.1%) | 10 (38.5%) |  |
| > 2 | 10 (17.9%) | 16 (61.5%) |  |
| PSA | 7.54 | 24.8 | <0.001 |
| Gleason score: |  |  | 0.010 |
| < 8 | 53 (94.6%) | 19 (73.1%) |  |
| >= 8 | 3 (5.36%) | 7 (26.9%) |  |

| Clinicopathological characteristics of the GSE70768 cohort | | | |
| --- | --- | --- | --- |
|  | RAECsig-low (N=74) | RAECsig-high (N=37) | P |
| Age: |  |  | 0.542 |
| <= 60 | 34 (45.9%) | 14 (37.8%) |  |
| >60 | 40 (54.1%) | 23 (62.2%) |  |
| Relapse: |  |  | 0.006 |
| No | 67 (90.5%) | 25 (67.6%) |  |
| Yes | 7 (9.46%) | 12 (32.4%) |  |
| pT: |  |  | 1.000 |
| <= 2 | 23 (31.1%) | 11 (29.7%) |  |
| > 2 | 51 (68.9%) | 26 (70.3%) |  |
| pN: |  |  | 1.000 |
| 0 | 55 (74.3%) | 27 (73.0%) |  |
| 1 | 5 (6.76%) | 3 (8.11%) |  |
| Unknown | 14 (18.9%) | 7 (18.9%) |  |
| PSA | 7.55 | 8.6 | 0.250 |
| Gleason score: |  |  | 0.058 |
| < 8 | 71 (95.9%) | 31 (83.8%) |  |
| >= 8 | 3 (4.05%) | 6 (16.2%) |  |

|  |  |  |  |
| --- | --- | --- | --- |
| Clinicopathological characteristics of the GSE70769 cohort | | | |
|  | RAECsig-low (N=64) | RAECsig-high (N=28) | P |
| Relapse: |  |  | <0.001 |
| No | 41 (64.1%) | 6 (21.4%) |  |
| Yes | 23 (35.9%) | 22 (78.6%) |  |
| pT: |  |  | 0.050 |
| <= 2 | 38 (59.4%) | 10 (35.7%) |  |
| > 2 | 24 (37.5%) | 18 (64.3%) |  |
| Unknown | 2 (3.12%) | 0 (0.00%) |  |
| PSA | 7.8 | 8.1 | 0.219 |
| Gleason score: |  |  | 0.003 |
| < 8 | 57 (89.1%) | 18 (64.3%) |  |
| >= 8 | 5 (7.81%) | 10 (35.7%) |  |
| Unknown | 2 (3.12%) | 0 (0.00%) |  |
|  |  |  |  |

| Clinicopathological characteristics of the GSE94767 cohort | | | |
| --- | --- | --- | --- |
|  | RAECsig-low (N=88) | RAECsig-high (N=44) | P |
| Relapse: |  |  | 0.015 |
| No | 71 (80.7%) | 26 (59.1%) |  |
| Yes | 17 (19.3%) | 18 (40.9%) |  |
| pT: |  |  | 0.496 |
| <= 2 | 51 (58.0%) | 22 (50.0%) |  |
| > 2 | 37 (42.0%) | 22 (50.0%) |  |
| PSA | 8.2 | 7.9 | 0.748 |
| Gleason score: |  |  | 0.002 |
| < 8 | 86 (97.7%) | 36 (81.8%) |  |
| >= 8 | 2 (2.27%) | 8 (18.2%) |  |
|  |  |  |  |
| Clinicopathological characteristics of the GSE21034 cohort | | | |
|  | RAECsig-low (N=77) | RAECsig-high (N=54) | P |
| Relapse: |  |  | 0.055 |
| No | 66 (85.70%) | 38 (70.40%) |  |
| Yes | 11 (14.30%) | 16 (29.60%) |  |
| pT: |  |  | 1.000 |
| <= 2 | 74 (96.10%) | 52 (96.30%) |  |
| > 2 | 3 (3.90%) | 2 (3.70%) |  |
| Gleason score: |  |  | 0.001 |
| < 8 | 74 (96.10%) | 41 (75.90%) |  |
| >= 8 | 3 (3.90%) | 12 (22.20%) |  |
| Unknown | 0 (0.00%) | 1 (1.85%) |  |

| **Table S3 Hyperparameters used in the machine learning algorithms.** | | | |  |  |  |
| --- | --- | --- | --- | --- | --- | --- |
| Algorithms | Package | Function | Parameter | Value | Range | Step |
| lasso | glmnet（R） | cv.glmnet | alpha | 1 | - | - |
|  |  |  | nfold | 5 | - | - |
|  |  | predict | s | lambda.min from cv.glmnet | - | - |
| Ridge | glmnet（R） | cv.glmnet | alpha | 0 | - | - |
|  |  |  | nfold | 5 | - | - |
|  |  | predict | s | lambda.min from cv.glmnet | - | - |
| Enet | glmnet（R） | cv.glmnet | alpha | - | [0.1,0.9] | 0.1 |
|  |  |  | nfold | 5 | - | - |
|  |  | predict | s | lambda.min from cv.glmnet | - | - |
| plsRCox | plsRcox（R） | cv.plsRcox | nt | 10 | - | - |
|  |  |  | nfold | 5 | - | - |
|  |  | plsRcox | nt | nt from cv.plsRcox | - | - |
| SuperPC | superpc（R） | superpc.cv | n.fold | 5 | - | - |
|  |  | superpc.predict | threshold | threshold from superpc.cv | - | - |
| XGBoost | xgboost（R） | xgboost | max_depth | - | [3,11] | 1 |
|  |  |  | min_child_weight | - | [3,18] | 1 |
|  |  |  | gamma | - | [0,0.15] | 0.01 |
|  |  |  | subsample | - | [0.4,0.95] | 0.05 |
|  |  |  | colsample_bytree | - | [0.4,0.95] | 0.05 |
|  |  |  | alpha | - | [0,1.5] | 0.1 |
|  |  |  | lambda | - | [0,1.5] | 0.1 |
|  |  |  | eta | - | [0.01,0.13] | 0.01 |
|  |  |  | n.trees | - | - | - |
| coxph (Forward) | survival (R) | coxph | - | - | - | - |
|  |  | step | direction | forward | - | - |
| coxph (Backward) | survival (R) | coxph | - | - | - | - |
|  |  | step | direction | backward | - | - |
| coxph (Both) | survival (R) | coxph | - | - | - | - |
|  |  | step | direction | both | - | - |
| RSF | randomForestSRC (R) | rfsrc | ntree | 100/500/1000/1500/2000 | - | - |
|  |  |  | mtry | - | [1,18] | 1 |
| GBM | gbm (R) | gbm | n.trees | n.trees from gbm | - | - |
|  |  |  | cv.folds | 5 | - | - |
| survival-SVM | survivalsvm (R) | survivalsvm | gamma.mu | 0.0001/0.001/0.01/0.1/1/2/5/10 | - | - |
|  |  |  | kernel | rbf_kernel | - | - |
| CoxBoost | CoxBoost (R) | optimCoxBoostPenalty | start.penalty | 500 | - | - |
|  |  | cv.CoxBoost | K | 5 | - | - |
|  |  |  | penalty | penalty from optimCoxBoostPenalty | - | - |
|  |  | CoxBoost | stepno | optimal.step from cv.CoxBoost | - | - |
|  |  |  | penalty | penalty from optimCoxBoostPenalty | - | - |

| **Table S4 Primer sequences of genes used in real-time qPCR** | | |
| --- | --- | --- |
| Gene | Forward sequence (5'-3') | Reverse sequence (5'-3') |
| FSCN1 | CTGCTACTTTGACATCGAGTGG | GGGCGGTTGATGAGCTTCA |
| TMEM255B | GCTCTGGTTTGTGGGGTCTC | TCTATTCTCCACCAAGTTGATGC |
| GABRD | AGAGCTACGGTTACTCATCGG | GGCCAGCGGACTTGAAGTT |
| GAPDH | GTCTCCTCTGACTTCAACAGCG | ACCACCCTGTTGCTGTAGCCAA |

| **Table S5 Results of differential gene analysis between RAECs and non-RAECs in the Chen dataset** | | | | |
| --- | --- | --- | --- | --- |
| Gene | Average log2FC | pct.1 | pct.2 | Adjusted P value |
| COL4A1 | 1.209 | 0.723 | 0.482 | 0.000 |
| COL4A2 | 1.066 | 0.709 | 0.435 | 0.000 |
| ESM1 | 1.042 | 0.375 | 0.198 | 0.000 |
| THY1 | 1.008 | 0.533 | 0.267 | 0.000 |
| CA2 | 0.890 | 0.406 | 0.185 | 0.000 |
| LBH | 0.858 | 0.418 | 0.228 | 0.000 |
| RGCC | 0.847 | 0.723 | 0.472 | 0.000 |
| SPARC | 0.779 | 0.870 | 0.762 | 0.000 |
| CCND1 | 0.704 | 0.424 | 0.228 | 0.000 |
| KCNE3 | 0.702 | 0.193 | 0.069 | 0.000 |
| LINC00152 | 0.682 | 0.476 | 0.288 | 0.000 |
| GABRD | 0.669 | 0.282 | 0.100 | 0.000 |
| MIR4435-2HG | 0.665 | 0.427 | 0.242 | 0.000 |
| SOX4 | 0.654 | 0.576 | 0.407 | 0.000 |
| IVNS1ABP | 0.641 | 0.519 | 0.352 | 0.000 |
| APLNR | 0.637 | 0.326 | 0.132 | 0.000 |
| NRP2 | 0.628 | 0.470 | 0.289 | 0.000 |
| ISG15 | 0.620 | 0.729 | 0.541 | 0.000 |
| TNFRSF4 | 0.619 | 0.308 | 0.144 | 0.000 |
| CYBA | 0.600 | 0.565 | 0.360 | 0.000 |
| PDGFB | 0.598 | 0.317 | 0.204 | 0.000 |
| VWA1 | 0.594 | 0.651 | 0.456 | 0.000 |
| CPM | 0.588 | 0.395 | 0.214 | 0.000 |
| PDGFD | 0.577 | 0.522 | 0.315 | 0.000 |
| TMEM233 | 0.574 | 0.386 | 0.217 | 0.000 |
| MCAM | 0.574 | 0.499 | 0.307 | 0.000 |
| PLVAP | 0.574 | 0.853 | 0.726 | 0.000 |
| GMFG | 0.564 | 0.611 | 0.432 | 0.000 |
| PLPP3 | 0.558 | 0.744 | 0.535 | 0.000 |
| TP53I11 | 0.557 | 0.311 | 0.142 | 0.000 |
| PLAUR | 0.551 | 0.239 | 0.118 | 0.001 |
| LAMA4 | 0.550 | 0.380 | 0.208 | 0.000 |
| PFN1 | 0.537 | 0.804 | 0.777 | 0.000 |
| LDHB | 0.536 | 0.591 | 0.443 | 0.000 |
| RAB13 | 0.535 | 0.741 | 0.581 | 0.000 |
| INSR | 0.521 | 0.769 | 0.653 | 0.000 |
| CALCRL | 0.519 | 0.686 | 0.545 | 0.000 |
| GPX1 | 0.519 | 0.793 | 0.687 | 0.000 |
| DYSF | 0.502 | 0.412 | 0.203 | 0.000 |
| CLIC1 | 0.491 | 0.787 | 0.644 | 0.000 |
| FLT1 | 0.489 | 0.735 | 0.627 | 0.000 |
| FAM101B | 0.488 | 0.424 | 0.258 | 0.000 |
| PIEZO2 | 0.479 | 0.294 | 0.130 | 0.000 |
| KDR | 0.478 | 0.530 | 0.382 | 0.004 |
| WDR60 | 0.477 | 0.427 | 0.282 | 0.006 |
| CALM1 | 0.476 | 0.870 | 0.852 | 0.000 |
| C1orf54 | 0.472 | 0.481 | 0.282 | 0.000 |
| ARPC2 | 0.470 | 0.755 | 0.594 | 0.000 |
| SERPINH1 | 0.466 | 0.622 | 0.451 | 0.000 |
| ARHGDIB | 0.461 | 0.657 | 0.535 | 0.000 |
| IGFBP7 | 0.458 | 0.974 | 0.955 | 0.000 |
| SLIRP | 0.455 | 0.571 | 0.413 | 0.000 |
| RBP1 | 0.453 | 0.401 | 0.226 | 0.000 |
| AP2S1 | 0.452 | 0.611 | 0.471 | 0.000 |
| JAG1 | 0.451 | 0.565 | 0.397 | 0.000 |
| TIMM13 | 0.445 | 0.478 | 0.330 | 0.001 |
| RBP5 | 0.441 | 0.389 | 0.227 | 0.000 |
| TMEM204 | 0.440 | 0.634 | 0.513 | 0.038 |
| YWHAH | 0.436 | 0.594 | 0.461 | 0.004 |
| MARCKSL1 | 0.436 | 0.720 | 0.571 | 0.000 |
| NID1 | 0.436 | 0.308 | 0.149 | 0.000 |
| PPIB | 0.431 | 0.778 | 0.671 | 0.000 |
| SMTN | 0.428 | 0.395 | 0.230 | 0.000 |
| ACTB | 0.427 | 0.957 | 0.955 | 0.000 |
| CDH13 | 0.425 | 0.409 | 0.266 | 0.004 |
| IQGAP2 | 0.424 | 0.236 | 0.109 | 0.000 |
| MANF | 0.419 | 0.441 | 0.287 | 0.003 |
| CALR | 0.418 | 0.729 | 0.586 | 0.000 |
| COL15A1 | 0.418 | 0.625 | 0.449 | 0.000 |
| RPS26 | 0.417 | 0.873 | 0.760 | 0.000 |
| MYL12B | 0.411 | 0.908 | 0.856 | 0.000 |
| POMP | 0.409 | 0.775 | 0.688 | 0.000 |
| COPS8 | 0.409 | 0.383 | 0.223 | 0.000 |
| S100A16 | 0.408 | 0.669 | 0.557 | 0.003 |
| GJA1 | 0.407 | 0.568 | 0.418 | 0.016 |
| ARHGAP18 | 0.407 | 0.401 | 0.263 | 0.024 |
| LUZP1 | 0.406 | 0.625 | 0.452 | 0.000 |
| CLEC11A | 0.405 | 0.231 | 0.105 | 0.000 |
| HTRA1 | 0.397 | 0.559 | 0.408 | 0.004 |
| CFL1 | 0.395 | 0.893 | 0.841 | 0.000 |
| AKIP1 | 0.394 | 0.251 | 0.112 | 0.000 |
| EDNRB | 0.393 | 0.499 | 0.300 | 0.000 |
| HECW2 | 0.392 | 0.378 | 0.204 | 0.000 |
| SPRY4 | 0.391 | 0.447 | 0.255 | 0.000 |
| TAX1BP3 | 0.388 | 0.441 | 0.315 | 0.022 |
| FAM198B | 0.386 | 0.585 | 0.446 | 0.029 |
| SNRPB | 0.385 | 0.602 | 0.457 | 0.002 |
| DOCK6 | 0.382 | 0.386 | 0.226 | 0.000 |
| PXDN | 0.380 | 0.282 | 0.167 | 0.042 |
| HSPG2 | 0.379 | 0.821 | 0.734 | 0.000 |
| JAK1 | 0.378 | 0.602 | 0.469 | 0.009 |
| PSME2 | 0.377 | 0.594 | 0.438 | 0.006 |
| APEX1 | 0.377 | 0.360 | 0.207 | 0.000 |
| COL6A2 | 0.376 | 0.297 | 0.147 | 0.000 |
| TGFB1 | 0.376 | 0.360 | 0.222 | 0.011 |
| FSCN1 | 0.372 | 0.375 | 0.234 | 0.009 |
| RHOC | 0.370 | 0.712 | 0.608 | 0.034 |
| NDUFS6 | 0.369 | 0.579 | 0.418 | 0.002 |
| FRMD8 | 0.368 | 0.300 | 0.156 | 0.000 |
| IFI6 | 0.367 | 0.755 | 0.631 | 0.026 |
| TM4SF18 | 0.367 | 0.599 | 0.450 | 0.013 |
| MAP1B | 0.365 | 0.329 | 0.189 | 0.002 |
| DGKD | 0.365 | 0.274 | 0.130 | 0.000 |
| HSP90B1 | 0.365 | 0.767 | 0.697 | 0.048 |
| AFAP1L1 | 0.364 | 0.317 | 0.163 | 0.000 |
| RASGRP3 | 0.364 | 0.346 | 0.179 | 0.000 |
| LAMC1 | 0.364 | 0.418 | 0.265 | 0.003 |
| SPRED1 | 0.363 | 0.262 | 0.114 | 0.000 |
| HMGB1 | 0.363 | 0.908 | 0.888 | 0.000 |
| LAYN | 0.360 | 0.277 | 0.150 | 0.006 |
| TMEM255B | 0.356 | 0.504 | 0.340 | 0.001 |
| H2AFY | 0.354 | 0.450 | 0.302 | 0.014 |
| SNRPG | 0.354 | 0.582 | 0.440 | 0.020 |
| BTNL9 | 0.352 | 0.369 | 0.221 | 0.002 |
| EXOC3L2 | 0.351 | 0.331 | 0.199 | 0.016 |
| PCDH12 | 0.349 | 0.222 | 0.105 | 0.001 |
| FKBP1A | 0.348 | 0.893 | 0.863 | 0.001 |
| UACA | 0.346 | 0.579 | 0.425 | 0.015 |
| KLHL5 | 0.346 | 0.380 | 0.221 | 0.000 |
| BCL6B | 0.345 | 0.256 | 0.122 | 0.000 |
| ALDOA | 0.345 | 0.654 | 0.523 | 0.015 |
| PSMA4 | 0.345 | 0.591 | 0.450 | 0.042 |
| GYPC | 0.342 | 0.386 | 0.235 | 0.001 |
| S100A11 | 0.342 | 0.844 | 0.766 | 0.020 |
| ST5 | 0.338 | 0.190 | 0.079 | 0.000 |
| CRIP2 | 0.337 | 0.818 | 0.744 | 0.003 |
| ITGA1 | 0.333 | 0.401 | 0.256 | 0.011 |
| LOXL2 | 0.332 | 0.216 | 0.102 | 0.002 |
| PSMA7 | 0.331 | 0.752 | 0.665 | 0.008 |
| YWHAB | 0.330 | 0.755 | 0.703 | 0.005 |
| KANK2 | 0.328 | 0.245 | 0.119 | 0.001 |
| CFDP1 | 0.327 | 0.487 | 0.342 | 0.042 |
| EIF4EBP1 | 0.326 | 0.256 | 0.125 | 0.000 |
| NID2 | 0.325 | 0.184 | 0.069 | 0.000 |
| ANGPTL2 | 0.322 | 0.196 | 0.089 | 0.003 |
| ETS1 | 0.321 | 0.568 | 0.405 | 0.003 |
| MYO1B | 0.320 | 0.308 | 0.158 | 0.000 |
| GNG11 | 0.320 | 0.899 | 0.889 | 0.000 |
| FLT4 | 0.318 | 0.300 | 0.163 | 0.001 |
| ACTG1 | 0.316 | 0.893 | 0.873 | 0.002 |
| TWF2 | 0.311 | 0.245 | 0.121 | 0.001 |
| ATP5E | 0.310 | 0.925 | 0.881 | 0.000 |
| PSMA5 | 0.306 | 0.369 | 0.222 | 0.001 |
| CHST15 | 0.306 | 0.207 | 0.097 | 0.004 |
| PTP4A3 | 0.303 | 0.265 | 0.143 | 0.008 |
| JUP | 0.301 | 0.254 | 0.129 | 0.001 |
| NDUFAB1 | 0.297 | 0.496 | 0.342 | 0.010 |
| TNFAIP8L1 | 0.291 | 0.280 | 0.154 | 0.009 |
| TIMP1 | 0.288 | 0.787 | 0.653 | 0.020 |
| DDI2 | 0.286 | 0.210 | 0.103 | 0.014 |
| TSPAN15 | 0.283 | 0.210 | 0.092 | 0.000 |
| SVIL | 0.281 | 0.366 | 0.223 | 0.002 |
| PPIA | 0.279 | 0.873 | 0.814 | 0.027 |
| ADRM1 | 0.279 | 0.418 | 0.272 | 0.006 |
| FYN | 0.274 | 0.458 | 0.314 | 0.031 |
| TDG | 0.272 | 0.354 | 0.224 | 0.032 |
| GSN | 0.270 | 0.916 | 0.890 | 0.011 |
| IDH2 | 0.264 | 0.245 | 0.127 | 0.007 |
| CRISPLD1 | 0.264 | 0.156 | 0.055 | 0.000 |
| ADAMTS5 | 0.263 | 0.115 | 0.039 | 0.011 |
| CD63 | 0.261 | 0.876 | 0.873 | 0.002 |
| CHRM3 | 0.259 | 0.254 | 0.132 | 0.002 |
| SEPT2 | 0.255 | 0.530 | 0.383 | 0.040 |
| MYL6 | 0.255 | 0.957 | 0.943 | 0.001 |
| YES1 | 0.252 | 0.331 | 0.194 | 0.004 |
| VIM | -0.256 | 0.922 | 0.959 | 0.001 |
| FTH1 | -0.291 | 0.951 | 0.944 | 0.001 |
| S100A6 | -0.300 | 0.795 | 0.879 | 0.000 |
| TOMM7 | -0.331 | 0.735 | 0.781 | 0.039 |
| PLSCR4 | -0.348 | 0.049 | 0.140 | 0.014 |
| H3F3B | -0.352 | 0.925 | 0.940 | 0.000 |
| SAT1 | -0.356 | 0.784 | 0.801 | 0.023 |
| TMCC3 | -0.360 | 0.288 | 0.307 | 0.014 |
| POLE4 | -0.363 | 0.311 | 0.395 | 0.023 |
| TACC1 | -0.364 | 0.669 | 0.755 | 0.006 |
| LMCD1 | -0.367 | 0.334 | 0.476 | 0.034 |
| POSTN | -0.368 | 0.089 | 0.198 | 0.002 |
| METTL7A | -0.376 | 0.121 | 0.216 | 0.040 |
| AHNAK | -0.380 | 0.473 | 0.595 | 0.004 |
| HLF | -0.388 | 0.043 | 0.127 | 0.040 |
| NPR3 | -0.392 | 0.049 | 0.135 | 0.031 |
| CST3 | -0.393 | 0.692 | 0.762 | 0.010 |
| STOM | -0.406 | 0.602 | 0.694 | 0.039 |
| FMO2 | -0.415 | 0.029 | 0.116 | 0.003 |
| HLA-DRA | -0.416 | 0.738 | 0.840 | 0.000 |
| TSC22D3 | -0.423 | 0.677 | 0.757 | 0.001 |
| FOXO3 | -0.425 | 0.282 | 0.366 | 0.004 |
| ACTN1 | -0.426 | 0.135 | 0.256 | 0.019 |
| TIPARP | -0.430 | 0.118 | 0.202 | 0.016 |
| EIF4A2 | -0.431 | 0.617 | 0.689 | 0.000 |
| NPC2 | -0.431 | 0.585 | 0.655 | 0.012 |
| CD74 | -0.438 | 0.899 | 0.962 | 0.000 |
| C6orf62 | -0.441 | 0.303 | 0.379 | 0.025 |
| SOCS2 | -0.443 | 0.288 | 0.376 | 0.003 |
| LGALS3 | -0.448 | 0.349 | 0.446 | 0.002 |
| UBC | -0.448 | 0.916 | 0.954 | 0.000 |
| NTN4 | -0.450 | 0.075 | 0.173 | 0.005 |
| PNRC1 | -0.452 | 0.597 | 0.659 | 0.000 |
| MAFB | -0.453 | 0.104 | 0.216 | 0.024 |
| HLA-DPB1 | -0.460 | 0.556 | 0.681 | 0.000 |
| MIDN | -0.463 | 0.530 | 0.564 | 0.000 |
| SELP | -0.464 | 0.130 | 0.267 | 0.001 |
| STAT3 | -0.470 | 0.550 | 0.637 | 0.000 |
| DDX3X | -0.475 | 0.487 | 0.561 | 0.000 |
| TRA2B | -0.477 | 0.522 | 0.618 | 0.000 |
| PLK3 | -0.479 | 0.242 | 0.341 | 0.004 |
| TMTC1 | -0.480 | 0.164 | 0.298 | 0.001 |
| ABLIM1 | -0.481 | 0.069 | 0.205 | 0.000 |
| ZFP36L2 | -0.490 | 0.559 | 0.655 | 0.001 |
| YBX3 | -0.495 | 0.605 | 0.691 | 0.000 |
| IER5 | -0.496 | 0.187 | 0.307 | 0.001 |
| SDPR | -0.521 | 0.571 | 0.708 | 0.000 |
| SERPING1 | -0.528 | 0.196 | 0.327 | 0.002 |
| PDK4 | -0.529 | 0.378 | 0.478 | 0.015 |
| TFPI | -0.530 | 0.156 | 0.307 | 0.000 |
| TGFBR3 | -0.533 | 0.349 | 0.427 | 0.000 |
| NFKBIA | -0.534 | 0.735 | 0.791 | 0.000 |
| SERTAD1 | -0.535 | 0.499 | 0.592 | 0.000 |
| ZFAND5 | -0.536 | 0.510 | 0.587 | 0.000 |
| GLUL | -0.539 | 0.251 | 0.385 | 0.000 |
| MCL1 | -0.540 | 0.530 | 0.619 | 0.000 |
| BRD2 | -0.547 | 0.496 | 0.602 | 0.000 |
| TPD52L1 | -0.547 | 0.199 | 0.348 | 0.000 |
| FBLN2 | -0.547 | 0.014 | 0.121 | 0.000 |
| BTG1 | -0.548 | 0.870 | 0.881 | 0.000 |
| LEPR | -0.562 | 0.121 | 0.274 | 0.000 |
| FAM107A | -0.567 | 0.282 | 0.409 | 0.000 |
| FBLN5 | -0.568 | 0.046 | 0.134 | 0.033 |
| GPM6A | -0.569 | 0.095 | 0.234 | 0.000 |
| HEXIM1 | -0.579 | 0.334 | 0.457 | 0.000 |
| CCNL1 | -0.580 | 0.504 | 0.654 | 0.000 |
| ARL6IP1 | -0.584 | 0.504 | 0.651 | 0.000 |
| HES1 | -0.590 | 0.571 | 0.658 | 0.000 |
| IL1R1 | -0.592 | 0.153 | 0.262 | 0.000 |
| IL33 | -0.593 | 0.135 | 0.293 | 0.000 |
| CSRNP1 | -0.611 | 0.277 | 0.408 | 0.000 |
| NNMT | -0.612 | 0.245 | 0.444 | 0.000 |
| YME1L1 | -0.614 | 0.291 | 0.401 | 0.000 |
| CTSC | -0.616 | 0.340 | 0.472 | 0.000 |
| JUND | -0.623 | 0.464 | 0.609 | 0.000 |
| CTD-3252C9.4 | -0.626 | 0.110 | 0.238 | 0.000 |
| NR4A2 | -0.628 | 0.138 | 0.284 | 0.000 |
| RND1 | -0.633 | 0.190 | 0.318 | 0.001 |
| TXNIP | -0.640 | 0.752 | 0.873 | 0.000 |
| RHOB | -0.644 | 0.631 | 0.721 | 0.000 |
| CCL14 | -0.648 | 0.228 | 0.392 | 0.000 |
| SRGN | -0.654 | 0.683 | 0.769 | 0.000 |
| ZFP36L1 | -0.666 | 0.631 | 0.762 | 0.000 |
| CCL23 | -0.678 | 0.095 | 0.216 | 0.004 |
| CLDN5 | -0.694 | 0.484 | 0.476 | 0.017 |
| MT1X | -0.732 | 0.473 | 0.596 | 0.000 |
| PPP1R15A | -0.732 | 0.493 | 0.664 | 0.000 |
| CDKN1A | -0.743 | 0.418 | 0.600 | 0.000 |
| INTS6 | -0.750 | 0.262 | 0.422 | 0.000 |
| CYR61 | -0.758 | 0.331 | 0.387 | 0.001 |
| PLAT | -0.769 | 0.277 | 0.454 | 0.000 |
| KCTD12 | -0.772 | 0.231 | 0.398 | 0.000 |
| IRF1 | -0.787 | 0.476 | 0.625 | 0.000 |
| KLF2 | -0.795 | 0.643 | 0.798 | 0.000 |
| SLC2A3 | -0.805 | 0.533 | 0.709 | 0.000 |
| NR4A1 | -0.817 | 0.499 | 0.644 | 0.000 |
| IER3 | -0.837 | 0.265 | 0.409 | 0.000 |
| LIFR | -0.844 | 0.513 | 0.692 | 0.000 |
| DNAJB1 | -0.867 | 0.490 | 0.603 | 0.000 |
| SOCS3 | -0.876 | 0.490 | 0.697 | 0.000 |
| NFKBIZ | -0.884 | 0.210 | 0.415 | 0.000 |
| ACKR1 | -0.897 | 0.259 | 0.472 | 0.000 |
| MT1M | -0.922 | 0.124 | 0.237 | 0.000 |
| IER2 | -0.950 | 0.646 | 0.777 | 0.000 |
| CEBPD | -0.998 | 0.441 | 0.642 | 0.000 |
| DUSP1 | -1.011 | 0.729 | 0.887 | 0.000 |
| ATF3 | -1.106 | 0.239 | 0.427 | 0.000 |
| MT1E | -1.138 | 0.259 | 0.384 | 0.000 |
| KLF4 | -1.163 | 0.395 | 0.611 | 0.000 |
| EDN1 | -1.201 | 0.104 | 0.294 | 0.000 |
| C10orf10 | -1.238 | 0.153 | 0.335 | 0.000 |
| GADD45B | -1.239 | 0.481 | 0.685 | 0.000 |
| BTG2 | -1.245 | 0.271 | 0.502 | 0.000 |
| CLU | -1.325 | 0.274 | 0.586 | 0.000 |
| FOSB | -1.375 | 0.519 | 0.736 | 0.000 |
| JUNB | -1.456 | 0.674 | 0.873 | 0.000 |
| CTGF | -1.601 | 0.277 | 0.460 | 0.000 |
| JUN | -1.612 | 0.625 | 0.823 | 0.000 |
| CXCL2 | -1.728 | 0.107 | 0.295 | 0.000 |
| EGR1 | -1.793 | 0.403 | 0.707 | 0.000 |
| ZFP36 | -1.878 | 0.591 | 0.860 | 0.000 |
| FOS | -2.212 | 0.646 | 0.876 | 0.000 |
| RAEC, recurrence-associated endothelial cells. | | |  |  |
| A total of 54 RAEC-related genes are highlighted in color with blue and grey indicating the signature genes and remaining genes, respectively. | | | | |

| **Table S6 Upregulated genes in endothelial cells versus non-endothelial cells in scRNA-seq data from the Chen dataset** | | | | |
| --- | --- | --- | --- | --- |
| Gene | Average log2FC | pct.1 | pct.2 | Adjusted P value |
| IFI27 | 4.868 | 0.980 | 0.045 | < 0.001 |
| SPRY1 | 4.272 | 0.829 | 0.121 | < 0.001 |
| SPARCL1 | 3.870 | 0.963 | 0.084 | < 0.001 |
| ACKR1 | 3.601 | 0.442 | 0.008 | < 0.001 |
| PLVAP | 3.566 | 0.744 | 0.018 | < 0.001 |
| RAMP2 | 3.523 | 0.862 | 0.043 | < 0.001 |
| GNG11 | 3.473 | 0.891 | 0.042 | < 0.001 |
| VWF | 3.395 | 0.774 | 0.030 | < 0.001 |
| MGP | 3.377 | 0.875 | 0.133 | < 0.001 |
| GSN | 3.260 | 0.893 | 0.118 | < 0.001 |
| RGCC | 3.198 | 0.507 | 0.174 | < 0.001 |
| IGFBP7 | 3.184 | 0.957 | 0.145 | < 0.001 |
| IFITM3 | 3.169 | 0.983 | 0.321 | < 0.001 |
| A2M | 3.074 | 0.866 | 0.097 | < 0.001 |
| ENG | 3.022 | 0.794 | 0.030 | < 0.001 |
| CLDN5 | 3.012 | 0.477 | 0.010 | < 0.001 |
| FLT1 | 3.008 | 0.643 | 0.017 | < 0.001 |
| AQP1 | 2.982 | 0.655 | 0.018 | < 0.001 |
| SPARC | 2.963 | 0.777 | 0.079 | < 0.001 |
| IGFBP4 | 2.960 | 0.827 | 0.048 | < 0.001 |
| HSPG2 | 2.949 | 0.746 | 0.069 | < 0.001 |
| EMCN | 2.939 | 0.765 | 0.007 | < 0.001 |
| RNASE1 | 2.930 | 0.810 | 0.020 | < 0.001 |
| IGFBP3 | 2.903 | 0.315 | 0.043 | < 0.001 |
| CTGF | 2.706 | 0.434 | 0.027 | < 0.001 |
| PLPP3 | 2.701 | 0.565 | 0.123 | < 0.001 |
| HLA-E | 2.688 | 0.974 | 0.383 | < 0.001 |
| ADGRL4 | 2.683 | 0.748 | 0.005 | < 0.001 |
| ID3 | 2.668 | 0.810 | 0.163 | < 0.001 |
| PECAM1 | 2.651 | 0.743 | 0.027 | < 0.001 |
| TM4SF1 | 2.649 | 0.818 | 0.264 | < 0.001 |
| RAMP3 | 2.617 | 0.641 | 0.006 | < 0.001 |
| SDPR | 2.575 | 0.688 | 0.020 | < 0.001 |
| SOCS3 | 2.575 | 0.667 | 0.184 | < 0.001 |
| TCF4 | 2.537 | 0.779 | 0.124 | < 0.001 |
| ID1 | 2.524 | 0.716 | 0.150 | < 0.001 |
| IFITM2 | 2.500 | 0.908 | 0.214 | < 0.001 |
| COL4A1 | 2.493 | 0.516 | 0.040 | < 0.001 |
| EPAS1 | 2.493 | 0.781 | 0.159 | < 0.001 |
| INSR | 2.474 | 0.670 | 0.333 | < 0.001 |
| CRIP2 | 2.450 | 0.755 | 0.101 | < 0.001 |
| CLEC14A | 2.450 | 0.673 | 0.003 | < 0.001 |
| EGFL7 | 2.438 | 0.681 | 0.086 | < 0.001 |
| SLC9A3R2 | 2.430 | 0.632 | 0.365 | < 0.001 |
| VIM | 2.426 | 0.954 | 0.270 | < 0.001 |
| CAV1 | 2.416 | 0.727 | 0.059 | < 0.001 |
| CLU | 2.403 | 0.542 | 0.193 | < 0.001 |
| KLF2 | 2.379 | 0.776 | 0.262 | < 0.001 |
| VAMP5 | 2.372 | 0.731 | 0.099 | < 0.001 |
| TGFBR2 | 2.363 | 0.720 | 0.069 | < 0.001 |
| EMP1 | 2.356 | 0.672 | 0.115 | < 0.001 |
| GIMAP7 | 2.344 | 0.716 | 0.040 | < 0.001 |
| TIMP3 | 2.342 | 0.711 | 0.096 | < 0.001 |
| ENPP2 | 2.339 | 0.352 | 0.011 | < 0.001 |
| ESM1 | 2.306 | 0.223 | 0.007 | < 0.001 |
| SLC2A3 | 2.256 | 0.684 | 0.128 | < 0.001 |
| IFITM1 | 2.248 | 0.753 | 0.086 | < 0.001 |
| ECSCR.1 | 2.203 | 0.661 | 0.005 | < 0.001 |
| ITM2A | 2.192 | 0.694 | 0.074 | < 0.001 |
| CD93 | 2.187 | 0.632 | 0.019 | < 0.001 |
| CCL14 | 2.186 | 0.369 | 0.001 | < 0.001 |
| SLCO2A1 | 2.184 | 0.584 | 0.014 | < 0.001 |
| PTPRB | 2.157 | 0.634 | 0.011 | < 0.001 |
| ESAM | 2.153 | 0.640 | 0.026 | < 0.001 |
| HLA-DRB5 | 2.130 | 0.649 | 0.043 | < 0.001 |
| TSPAN7 | 2.129 | 0.608 | 0.007 | < 0.001 |
| HYAL2 | 2.094 | 0.584 | 0.123 | < 0.001 |
| LIFR | 2.090 | 0.667 | 0.245 | < 0.001 |
| CD34 | 2.079 | 0.575 | 0.006 | < 0.001 |
| LDB2 | 2.073 | 0.625 | 0.008 | < 0.001 |
| CALCRL | 2.070 | 0.565 | 0.005 | < 0.001 |
| PODXL | 2.056 | 0.525 | 0.031 | < 0.001 |
| CNN3 | 2.042 | 0.748 | 0.226 | < 0.001 |
| STC1 | 2.040 | 0.268 | 0.006 | < 0.001 |
| SEC14L1 | 2.030 | 0.642 | 0.131 | < 0.001 |
| STOM | 2.021 | 0.681 | 0.091 | < 0.001 |
| FKBP1A | 2.017 | 0.867 | 0.559 | < 0.001 |
| HLA-B | 1.997 | 0.985 | 0.524 | < 0.001 |
| ITM2B | 1.993 | 0.978 | 0.732 | < 0.001 |
| CD74 | 1.989 | 0.953 | 0.253 | < 0.001 |
| COL15A1 | 1.975 | 0.474 | 0.009 | < 0.001 |
| PTRF | 1.974 | 0.707 | 0.095 | < 0.001 |
| SPTBN1 | 1.972 | 0.724 | 0.270 | < 0.001 |
| TMEM204 | 1.964 | 0.530 | 0.024 | < 0.001 |
| TMEM88 | 1.962 | 0.522 | 0.043 | < 0.001 |
| PCAT19 | 1.951 | 0.611 | 0.028 | < 0.001 |
| COL4A2 | 1.950 | 0.473 | 0.044 | < 0.001 |
| ANGPT2 | 1.947 | 0.395 | 0.047 | < 0.001 |
| RBP7 | 1.933 | 0.425 | 0.055 | < 0.001 |
| CD59 | 1.927 | 0.800 | 0.401 | < 0.001 |
| APP | 1.923 | 0.852 | 0.446 | < 0.001 |
| TM4SF18 | 1.889 | 0.471 | 0.006 | < 0.001 |
| SELE | 1.870 | 0.204 | 0.003 | < 0.001 |
| TXNIP | 1.863 | 0.856 | 0.489 | < 0.001 |
| C10orf10 | 1.863 | 0.309 | 0.060 | < 0.001 |
| IGFBP5 | 1.841 | 0.139 | 0.050 | < 0.001 |
| RDX | 1.837 | 0.695 | 0.305 | < 0.001 |
| BST2 | 1.835 | 0.639 | 0.102 | < 0.001 |
| HLA-DRB1 | 1.832 | 0.871 | 0.161 | < 0.001 |
| THBD | 1.827 | 0.455 | 0.018 | < 0.001 |
| MEF2C | 1.826 | 0.643 | 0.080 | < 0.001 |
| VWA1 | 1.825 | 0.484 | 0.176 | < 0.001 |
| PLPP1 | 1.816 | 0.670 | 0.486 | < 0.001 |
| NOSTRIN | 1.815 | 0.516 | 0.036 | < 0.001 |
| EDNRB | 1.791 | 0.328 | 0.015 | < 0.001 |
| IFI6 | 1.787 | 0.648 | 0.200 | < 0.001 |
| CXorf36 | 1.787 | 0.525 | 0.003 | < 0.001 |
| TACC1 | 1.779 | 0.742 | 0.250 | < 0.001 |
| PDLIM1 | 1.777 | 0.578 | 0.095 | < 0.001 |
| NOTCH4 | 1.775 | 0.498 | 0.014 | < 0.001 |
| GIMAP4 | 1.773 | 0.579 | 0.043 | < 0.001 |
| PLAT | 1.765 | 0.429 | 0.033 | < 0.001 |
| MTUS1 | 1.760 | 0.556 | 0.127 | < 0.001 |
| ADGRF5 | 1.755 | 0.488 | 0.016 | < 0.001 |
| S100A16 | 1.754 | 0.573 | 0.024 | < 0.001 |
| ARHGAP29 | 1.749 | 0.606 | 0.186 | < 0.001 |
| ETS2 | 1.738 | 0.624 | 0.109 | < 0.001 |
| KDR | 1.729 | 0.403 | 0.004 | < 0.001 |
| B2M | 1.726 | 1.000 | 0.926 | < 0.001 |
| MMRN2 | 1.720 | 0.515 | 0.005 | < 0.001 |
| NNMT | 1.720 | 0.416 | 0.054 | < 0.001 |
| PALMD | 1.715 | 0.506 | 0.049 | < 0.001 |
| HLA-A | 1.706 | 0.963 | 0.414 | < 0.001 |
| IFI16 | 1.700 | 0.654 | 0.106 | < 0.001 |
| SH3BP5 | 1.698 | 0.528 | 0.065 | < 0.001 |
| XAF1 | 1.693 | 0.513 | 0.041 | < 0.001 |
| HLA-C | 1.689 | 0.965 | 0.638 | < 0.001 |
| IL6ST | 1.661 | 0.680 | 0.291 | < 0.001 |
| CCL23 | 1.658 | 0.199 | 0.004 | < 0.001 |
| APOLD1 | 1.655 | 0.451 | 0.047 | < 0.001 |
| CDH5 | 1.643 | 0.490 | 0.003 | < 0.001 |
| GJA1 | 1.641 | 0.439 | 0.014 | < 0.001 |
| PRSS23 | 1.640 | 0.648 | 0.288 | < 0.001 |
| CYYR1 | 1.632 | 0.494 | 0.002 | < 0.001 |
| ICAM2 | 1.631 | 0.455 | 0.024 | < 0.001 |
| EHD4 | 1.629 | 0.530 | 0.109 | < 0.001 |
| MYL12A | 1.628 | 0.911 | 0.652 | < 0.001 |
| LMCD1 | 1.608 | 0.456 | 0.023 | < 0.001 |
| TMSB10 | 1.605 | 0.997 | 0.926 | < 0.001 |
| NCOA7 | 1.600 | 0.506 | 0.075 | < 0.001 |
| TSC22D1 | 1.599 | 0.814 | 0.570 | < 0.001 |
| EDN1 | 1.587 | 0.267 | 0.097 | < 0.001 |
| JAM2 | 1.581 | 0.475 | 0.005 | < 0.001 |
| ITGA6 | 1.568 | 0.523 | 0.117 | < 0.001 |
| SRP14 | 1.566 | 0.936 | 0.867 | < 0.001 |
| KCTD12 | 1.564 | 0.374 | 0.062 | < 0.001 |
| PDGFD | 1.563 | 0.344 | 0.006 | < 0.001 |
| S100A13 | 1.545 | 0.591 | 0.143 | < 0.001 |
| HEG1 | 1.542 | 0.483 | 0.030 | < 0.001 |
| UACA | 1.538 | 0.447 | 0.100 | < 0.001 |
| TSHZ2 | 1.531 | 0.446 | 0.069 | < 0.001 |
| WWTR1 | 1.529 | 0.534 | 0.116 | < 0.001 |
| S1PR1 | 1.517 | 0.461 | 0.014 | < 0.001 |
| S100A6 | 1.516 | 0.867 | 0.320 | < 0.001 |
| FAM167B | 1.510 | 0.363 | 0.005 | < 0.001 |
| ISG15 | 1.506 | 0.568 | 0.191 | < 0.001 |
| TGFBR3 | 1.494 | 0.416 | 0.031 | < 0.001 |
| PDK4 | 1.494 | 0.464 | 0.097 | < 0.001 |
| ANXA2 | 1.488 | 0.763 | 0.336 | < 0.001 |
| NEDD9 | 1.474 | 0.571 | 0.164 | < 0.001 |
| THY1 | 1.469 | 0.305 | 0.021 | < 0.001 |
| GNAI2 | 1.468 | 0.621 | 0.250 | < 0.001 |
| FAM107A | 1.468 | 0.391 | 0.008 | < 0.001 |
| MSN | 1.467 | 0.578 | 0.108 | < 0.001 |
| PRCP | 1.451 | 0.448 | 0.160 | < 0.001 |
| S100A10 | 1.443 | 0.803 | 0.393 | < 0.001 |
| MGST2 | 1.442 | 0.734 | 0.427 | < 0.001 |
| LIMS2 | 1.438 | 0.440 | 0.011 | < 0.001 |
| ZFP36 | 1.435 | 0.822 | 0.649 | < 0.001 |
| PTPRG | 1.433 | 0.431 | 0.067 | < 0.001 |
| ELK3 | 1.424 | 0.468 | 0.031 | < 0.001 |
| SWAP70 | 1.424 | 0.526 | 0.153 | < 0.001 |
| SOX17 | 1.423 | 0.321 | 0.002 | < 0.001 |
| GAS6 | 1.421 | 0.474 | 0.050 | < 0.001 |
| ITGB1 | 1.416 | 0.725 | 0.480 | < 0.001 |
| YBX3 | 1.400 | 0.678 | 0.432 | < 0.001 |
| LRRC32 | 1.395 | 0.395 | 0.016 | < 0.001 |
| CALM1 | 1.391 | 0.854 | 0.700 | < 0.001 |
| CCDC85B | 1.383 | 0.682 | 0.404 | < 0.001 |
| FCGRT | 1.381 | 0.609 | 0.240 | < 0.001 |
| CTSC | 1.381 | 0.453 | 0.090 | < 0.001 |
| ABCG2 | 1.375 | 0.373 | 0.004 | < 0.001 |
| MYL12B | 1.375 | 0.864 | 0.705 | < 0.001 |
| COX7A1 | 1.365 | 0.506 | 0.041 | < 0.001 |
| HTRA1 | 1.362 | 0.429 | 0.089 | < 0.001 |
| NRP1 | 1.338 | 0.464 | 0.134 | < 0.001 |
| PCDH17 | 1.330 | 0.352 | 0.002 | < 0.001 |
| FAM198B | 1.330 | 0.466 | 0.171 | < 0.001 |
| ARL4A | 1.325 | 0.530 | 0.210 | < 0.001 |
| TAGLN2 | 1.324 | 0.630 | 0.253 | < 0.001 |
| ROBO4 | 1.321 | 0.372 | 0.003 | < 0.001 |
| ADCY4 | 1.304 | 0.368 | 0.007 | < 0.001 |
| MYH9 | 1.302 | 0.635 | 0.347 | < 0.001 |
| LEPROT | 1.301 | 0.638 | 0.359 | < 0.001 |
| ASAP1 | 1.300 | 0.471 | 0.113 | < 0.001 |
| DLC1 | 1.289 | 0.404 | 0.031 | < 0.001 |
| HES1 | 1.288 | 0.646 | 0.447 | < 0.001 |
| CA2 | 1.288 | 0.216 | 0.020 | < 0.001 |
| MEIS2 | 1.287 | 0.403 | 0.045 | < 0.001 |
| SNCG | 1.285 | 0.399 | 0.034 | < 0.001 |
| MYCT1 | 1.283 | 0.410 | 0.001 | < 0.001 |
| PLXND1 | 1.282 | 0.406 | 0.032 | < 0.001 |
| BMPR2 | 1.277 | 0.494 | 0.175 | < 0.001 |
| ENTPD1 | 1.277 | 0.406 | 0.034 | < 0.001 |
| CAV2 | 1.263 | 0.457 | 0.036 | < 0.001 |
| CDC37 | 1.261 | 0.653 | 0.391 | < 0.001 |
| NFIB | 1.250 | 0.730 | 0.501 | < 0.001 |
| CEBPD | 1.245 | 0.613 | 0.396 | < 0.001 |
| NUAK1 | 1.243 | 0.370 | 0.020 | < 0.001 |
| DOCK9 | 1.231 | 0.398 | 0.048 | < 0.001 |
| TIE1 | 1.230 | 0.380 | 0.005 | < 0.001 |
| TGM2 | 1.225 | 0.430 | 0.130 | < 0.001 |
| RND1 | 1.225 | 0.300 | 0.043 | < 0.001 |
| EMP2 | 1.215 | 0.571 | 0.293 | < 0.001 |
| TIMP2 | 1.207 | 0.465 | 0.084 | < 0.001 |
| AKR1C3 | 1.205 | 0.286 | 0.010 | < 0.001 |
| GRB10 | 1.198 | 0.371 | 0.048 | < 0.001 |
| LMO2 | 1.197 | 0.380 | 0.013 | < 0.001 |
| SOX18 | 1.197 | 0.333 | 0.003 | < 0.001 |
| IVNS1ABP | 1.193 | 0.375 | 0.190 | < 0.001 |
| CDA | 1.191 | 0.361 | 0.004 | < 0.001 |
| PIK3R3 | 1.188 | 0.416 | 0.118 | < 0.001 |
| TFPI | 1.182 | 0.286 | 0.014 | < 0.001 |
| IL33 | 1.180 | 0.271 | 0.002 | < 0.001 |
| CLIC4 | 1.179 | 0.443 | 0.122 | < 0.001 |
| NES | 1.176 | 0.332 | 0.015 | < 0.001 |
| AKAP12 | 1.173 | 0.303 | 0.155 | < 0.001 |
| ITGA5 | 1.169 | 0.393 | 0.043 | < 0.001 |
| LUZP1 | 1.164 | 0.476 | 0.185 | < 0.001 |
| DUSP23 | 1.164 | 0.429 | 0.235 | < 0.001 |
| PPFIBP1 | 1.164 | 0.414 | 0.119 | < 0.001 |
| IFI44L | 1.162 | 0.340 | 0.021 | < 0.001 |
| PLK2 | 1.154 | 0.413 | 0.136 | < 0.001 |
| GIMAP1 | 1.154 | 0.390 | 0.020 | < 0.001 |
| ERG | 1.153 | 0.365 | 0.005 | < 0.001 |
| FN1 | 1.151 | 0.298 | 0.113 | < 0.001 |
| DDIT4 | 1.143 | 0.554 | 0.368 | < 0.001 |
| ABL2 | 1.142 | 0.365 | 0.160 | < 0.001 |
| DPYSL2 | 1.141 | 0.427 | 0.055 | < 0.001 |
| TMEM255B | 1.138 | 0.363 | 0.014 | < 0.001 |
| ARPC1B | 1.136 | 0.629 | 0.258 | < 0.001 |
| KCNN3 | 1.135 | 0.311 | 0.002 | < 0.001 |
| SELP | 1.134 | 0.247 | 0.002 | < 0.001 |
| NRP2 | 1.133 | 0.314 | 0.033 | < 0.001 |
| IL3RA | 1.133 | 0.332 | 0.005 | < 0.001 |
| CCL2 | 1.130 | 0.147 | 0.047 | < 0.001 |
| CDKN1A | 1.124 | 0.575 | 0.394 | < 0.001 |
| NASP | 1.117 | 0.538 | 0.276 | < 0.001 |
| KIAA0355 | 1.114 | 0.464 | 0.192 | < 0.001 |
| CXCL2 | 1.112 | 0.268 | 0.169 | < 0.001 |
| POSTN | 1.111 | 0.183 | 0.004 | < 0.001 |
| MKL2 | 1.106 | 0.356 | 0.118 | < 0.001 |
| RBMS1 | 1.105 | 0.462 | 0.101 | < 0.001 |
| ETS1 | 1.104 | 0.428 | 0.064 | < 0.001 |
| GBP4 | 1.103 | 0.361 | 0.031 | < 0.001 |
| ZEB1 | 1.086 | 0.373 | 0.028 | < 0.001 |
| PRKCDBP | 1.079 | 0.379 | 0.037 | < 0.001 |
| DLL4 | 1.072 | 0.311 | 0.019 | < 0.001 |
| BCAM | 1.067 | 0.693 | 0.461 | < 0.001 |
| AP1S2 | 1.066 | 0.468 | 0.125 | < 0.001 |
| TIMP1 | 1.058 | 0.672 | 0.160 | < 0.001 |
| ARL15 | 1.057 | 0.284 | 0.044 | < 0.001 |
| CX3CL1 | 1.055 | 0.302 | 0.015 | < 0.001 |
| PTTG1IP | 1.054 | 0.537 | 0.338 | < 0.001 |
| UPP1 | 1.052 | 0.379 | 0.068 | < 0.001 |
| EFNB2 | 1.052 | 0.457 | 0.291 | < 0.001 |
| TNS2 | 1.051 | 0.376 | 0.078 | < 0.001 |
| BTNL9 | 1.050 | 0.242 | 0.005 | < 0.001 |
| PSMB9 | 1.047 | 0.501 | 0.120 | < 0.001 |
| PINK1 | 1.045 | 0.394 | 0.174 | < 0.001 |
| DUSP6 | 1.036 | 0.416 | 0.114 | < 0.001 |
| KLF9 | 1.035 | 0.507 | 0.287 | < 0.001 |
| PIK3C2A | 1.035 | 0.449 | 0.228 | < 0.001 |
| SPRY4 | 1.029 | 0.282 | 0.034 | < 0.001 |
| CDH13 | 1.026 | 0.286 | 0.010 | < 0.001 |
| GSTP1 | 1.023 | 0.643 | 0.178 | < 0.001 |
| ARGLU1 | 1.023 | 0.719 | 0.558 | < 0.001 |
| ADAMTS1 | 1.023 | 0.370 | 0.195 | < 0.001 |
| TPM3 | 1.020 | 0.646 | 0.432 | < 0.001 |
| GMFG | 1.018 | 0.457 | 0.121 | < 0.001 |
| BHLHE40 | 1.017 | 0.386 | 0.172 | < 0.001 |
| NRN1 | 1.014 | 0.319 | 0.030 | < 0.001 |
| TINAGL1 | 1.014 | 0.379 | 0.038 | < 0.001 |
| POMP | 1.013 | 0.700 | 0.606 | < 0.001 |
| FAM110D | 1.012 | 0.273 | 0.003 | < 0.001 |
| HLA-DMA | 1.011 | 0.480 | 0.100 | < 0.001 |
| PSMB8 | 1.009 | 0.451 | 0.128 | < 0.001 |
| YWHAH | 1.009 | 0.479 | 0.278 | < 0.001 |
| ADAMTS9 | 1.006 | 0.266 | 0.018 | < 0.001 |
| PLSCR1 | 1.003 | 0.369 | 0.074 | < 0.001 |
| RBP5 | 0.999 | 0.249 | 0.003 | < 0.001 |
| GPM6A | 0.999 | 0.214 | 0.001 | < 0.001 |
| HLA-DRA | 0.999 | 0.825 | 0.217 | < 0.001 |
| ZFP36L1 | 0.998 | 0.743 | 0.568 | < 0.001 |
| MCAM | 0.997 | 0.334 | 0.040 | < 0.001 |
| LEPR | 0.995 | 0.253 | 0.010 | < 0.001 |
| PLS3 | 0.992 | 0.328 | 0.025 | < 0.001 |
| TMTC1 | 0.988 | 0.279 | 0.009 | < 0.001 |
| FAM101B | 0.987 | 0.282 | 0.012 | < 0.001 |
| SEPW1 | 0.984 | 0.773 | 0.618 | < 0.001 |
| PRMT1 | 0.984 | 0.475 | 0.273 | < 0.001 |
| SHROOM4 | 0.978 | 0.305 | 0.004 | < 0.001 |
| PTPN14 | 0.977 | 0.359 | 0.113 | < 0.001 |
| OLFM1 | 0.976 | 0.227 | 0.052 | < 0.001 |
| FXYD5 | 0.974 | 0.587 | 0.164 | < 0.001 |
| IRF1 | 0.974 | 0.604 | 0.463 | < 0.001 |
| CRIM1 | 0.974 | 0.357 | 0.090 | < 0.001 |
| ID2 | 0.974 | 0.662 | 0.429 | < 0.001 |
| THSD7A | 0.972 | 0.356 | 0.183 | < 0.001 |
| GIMAP6 | 0.969 | 0.324 | 0.012 | < 0.001 |
| OSBPL1A | 0.969 | 0.337 | 0.130 | < 0.001 |
| DAB2 | 0.967 | 0.425 | 0.141 | < 0.001 |
| TNFRSF10D | 0.966 | 0.278 | 0.022 | < 0.001 |
| PTPN12 | 0.966 | 0.407 | 0.180 | < 0.001 |
| F8 | 0.965 | 0.280 | 0.028 | < 0.001 |
| SASH1 | 0.960 | 0.397 | 0.184 | < 0.001 |
| IFNGR1 | 0.958 | 0.392 | 0.205 | < 0.001 |
| ACTN4 | 0.955 | 0.547 | 0.369 | < 0.001 |
| TPM4 | 0.949 | 0.558 | 0.356 | < 0.001 |
| KLF4 | 0.948 | 0.581 | 0.456 | < 0.001 |
| FRY | 0.947 | 0.309 | 0.029 | < 0.001 |
| RNF115 | 0.937 | 0.436 | 0.226 | < 0.001 |
| MAGI1 | 0.937 | 0.334 | 0.143 | < 0.001 |
| TMSB4X | 0.936 | 1.000 | 0.980 | < 0.001 |
| FZD4 | 0.935 | 0.328 | 0.080 | < 0.001 |
| TSPAN4 | 0.933 | 0.337 | 0.055 | < 0.001 |
| GFOD1 | 0.923 | 0.304 | 0.031 | < 0.001 |
| RP11-382A20.3 | 0.921 | 0.370 | 0.149 | < 0.001 |
| MIR4435-2HG | 0.921 | 0.268 | 0.060 | < 0.001 |
| RAPGEF4 | 0.921 | 0.270 | 0.019 | < 0.001 |
| PMP22 | 0.921 | 0.330 | 0.045 | < 0.001 |
| FEZ2 | 0.918 | 0.412 | 0.191 | < 0.001 |
| RASAL2 | 0.917 | 0.331 | 0.105 | < 0.001 |
| TMEM173 | 0.912 | 0.359 | 0.054 | < 0.001 |
| SGK1 | 0.911 | 0.440 | 0.182 | < 0.001 |
| JUNB | 0.911 | 0.845 | 0.753 | < 0.001 |
| MPZL2 | 0.910 | 0.344 | 0.110 | < 0.001 |
| PHACTR2 | 0.908 | 0.420 | 0.183 | < 0.001 |
| MATN2 | 0.908 | 0.234 | 0.008 | < 0.001 |
| DNASE1L3 | 0.906 | 0.143 | 0.004 | < 0.001 |
| C1orf54 | 0.904 | 0.311 | 0.047 | < 0.001 |
| RPGR | 0.901 | 0.293 | 0.061 | < 0.001 |
| MIDN | 0.899 | 0.559 | 0.456 | < 0.001 |
| SERTAD1 | 0.895 | 0.579 | 0.415 | < 0.001 |
| LPAR6 | 0.895 | 0.352 | 0.098 | < 0.001 |
| HEY1 | 0.893 | 0.224 | 0.022 | < 0.001 |
| RAC1 | 0.892 | 0.860 | 0.742 | < 0.001 |
| TPD52L1 | 0.891 | 0.327 | 0.205 | < 0.001 |
| TEK | 0.890 | 0.267 | 0.001 | < 0.001 |
| RAB13 | 0.888 | 0.603 | 0.485 | < 0.001 |
| TMEM233 | 0.888 | 0.241 | 0.017 | < 0.001 |
| TMEM47 | 0.887 | 0.309 | 0.077 | < 0.001 |
| LIMCH1 | 0.887 | 0.484 | 0.329 | < 0.001 |
| TMCC3 | 0.887 | 0.304 | 0.097 | < 0.001 |
| PPIC | 0.884 | 0.337 | 0.081 | < 0.001 |
| RHOC | 0.883 | 0.622 | 0.510 | < 0.001 |
| HBEGF | 0.883 | 0.378 | 0.221 | < 0.001 |
| HDAC7 | 0.880 | 0.346 | 0.118 | < 0.001 |
| FCN3 | 0.880 | 0.159 | 0.001 | < 0.001 |
| ACVRL1 | 0.877 | 0.293 | 0.007 | < 0.001 |
| SERPINH1 | 0.873 | 0.475 | 0.269 | < 0.001 |
| FKBP9 | 0.872 | 0.314 | 0.087 | < 0.001 |
| FXYD6 | 0.871 | 0.288 | 0.028 | < 0.001 |
| CYTL1 | 0.870 | 0.171 | 0.008 | < 0.001 |
| ARHGEF15 | 0.870 | 0.260 | 0.002 | < 0.001 |
| CLEC2B | 0.869 | 0.405 | 0.086 | < 0.001 |
| PLXNA2 | 0.869 | 0.263 | 0.018 | < 0.001 |
| JAG1 | 0.868 | 0.420 | 0.281 | < 0.001 |
| MGST3 | 0.867 | 0.632 | 0.481 | < 0.001 |
| SNX3 | 0.866 | 0.670 | 0.543 | < 0.001 |
| RAPGEF3 | 0.866 | 0.250 | 0.016 | < 0.001 |
| NPDC1 | 0.865 | 0.767 | 0.571 | < 0.001 |
| LIMA1 | 0.861 | 0.440 | 0.269 | < 0.001 |
| MGLL | 0.861 | 0.315 | 0.063 | < 0.001 |
| RASIP1 | 0.860 | 0.269 | 0.004 | < 0.001 |
| ZBTB16 | 0.858 | 0.582 | 0.370 | < 0.001 |
| ADAM15 | 0.857 | 0.341 | 0.191 | < 0.001 |
| HECW2 | 0.856 | 0.229 | 0.002 | < 0.001 |
| CFI | 0.855 | 0.234 | 0.006 | < 0.001 |
| FERMT2 | 0.854 | 0.360 | 0.103 | < 0.001 |
| SERPING1 | 0.852 | 0.308 | 0.060 | < 0.001 |
| EPHA4 | 0.852 | 0.293 | 0.073 | < 0.001 |
| TJP1 | 0.849 | 0.537 | 0.375 | < 0.001 |
| PRDM1 | 0.847 | 0.330 | 0.062 | < 0.001 |
| LCN6 | 0.846 | 0.210 | 0.002 | < 0.001 |
| HLA-F | 0.846 | 0.423 | 0.104 | < 0.001 |
| F2R | 0.846 | 0.268 | 0.020 | < 0.001 |
| LAMA4 | 0.843 | 0.232 | 0.011 | < 0.001 |
| GADD45B | 0.842 | 0.656 | 0.540 | < 0.001 |
| ITGA1 | 0.840 | 0.277 | 0.050 | < 0.001 |
| LHFP | 0.840 | 0.379 | 0.050 | < 0.001 |
| CXCL12 | 0.839 | 0.207 | 0.081 | < 0.001 |
| CD99 | 0.837 | 0.683 | 0.554 | < 0.001 |
| TUBA1B | 0.836 | 0.663 | 0.525 | < 0.001 |
| RUNX1T1 | 0.833 | 0.250 | 0.012 | < 0.001 |
| GIMAP8 | 0.833 | 0.254 | 0.006 | < 0.001 |
| C8orf4 | 0.832 | 0.296 | 0.224 | < 0.001 |
| TIAM1 | 0.828 | 0.243 | 0.022 | < 0.001 |
| CD200 | 0.825 | 0.234 | 0.006 | < 0.001 |
| KLF7 | 0.823 | 0.320 | 0.100 | < 0.001 |
| SULF2 | 0.823 | 0.270 | 0.023 | < 0.001 |
| MALL | 0.820 | 0.272 | 0.051 | < 0.001 |
| DYSF | 0.820 | 0.233 | 0.004 | < 0.001 |
| JAK1 | 0.820 | 0.488 | 0.294 | < 0.001 |
| GUK1 | 0.818 | 0.794 | 0.684 | < 0.001 |
| NDRG1 | 0.817 | 0.588 | 0.466 | < 0.001 |
| SCARF1 | 0.816 | 0.252 | 0.010 | < 0.001 |
| TUBB6 | 0.816 | 0.270 | 0.030 | < 0.001 |
| TSPAN14 | 0.815 | 0.417 | 0.266 | < 0.001 |
| GNAS | 0.815 | 0.712 | 0.642 | < 0.001 |
| FSCN1 | 0.811 | 0.254 | 0.034 | < 0.001 |
| UTRN | 0.808 | 0.490 | 0.352 | < 0.001 |
| PDGFB | 0.808 | 0.220 | 0.034 | < 0.001 |
| CFLAR | 0.806 | 0.530 | 0.348 | < 0.001 |
| DAD1 | 0.806 | 0.677 | 0.571 | < 0.001 |
| VCAM1 | 0.806 | 0.134 | 0.007 | < 0.001 |
| DHRS3 | 0.804 | 0.375 | 0.267 | < 0.001 |
| MMP2 | 0.803 | 0.223 | 0.016 | < 0.001 |
| RBMS3 | 0.801 | 0.274 | 0.019 | < 0.001 |
| JMJD1C | 0.801 | 0.466 | 0.352 | < 0.001 |
| MECOM | 0.800 | 0.235 | 0.010 | < 0.001 |
| CD81 | 0.799 | 0.583 | 0.496 | < 0.001 |
| RAI14 | 0.798 | 0.265 | 0.040 | < 0.001 |
| DUSP1 | 0.797 | 0.864 | 0.754 | < 0.001 |
| LINC00152 | 0.797 | 0.315 | 0.092 | < 0.001 |
| LAPTM4A | 0.795 | 0.764 | 0.619 | < 0.001 |
| EXOC3L2 | 0.793 | 0.217 | 0.003 | < 0.001 |
| SNHG7 | 0.793 | 0.646 | 0.540 | < 0.001 |
| LAMB2 | 0.793 | 0.309 | 0.110 | < 0.001 |
| HMGB1 | 0.793 | 0.891 | 0.813 | < 0.001 |
| EID1 | 0.790 | 0.746 | 0.638 | < 0.001 |
| ELMO1 | 0.787 | 0.290 | 0.032 | < 0.001 |
| UBC | 0.787 | 0.949 | 0.886 | < 0.001 |
| PDE2A | 0.783 | 0.217 | 0.003 | < 0.001 |
| SYNE2 | 0.781 | 0.408 | 0.171 | < 0.001 |
| MAP1LC3B | 0.776 | 0.535 | 0.377 | < 0.001 |
| ARL2 | 0.775 | 0.461 | 0.323 | < 0.001 |
| CASKIN2 | 0.774 | 0.246 | 0.039 | < 0.001 |
| FBLN5 | 0.773 | 0.122 | 0.006 | < 0.001 |
| YWHAB | 0.773 | 0.710 | 0.629 | < 0.001 |
| HLA-DPA1 | 0.773 | 0.673 | 0.164 | < 0.001 |
| WSB1 | 0.772 | 0.649 | 0.523 | < 0.001 |
| RBP1 | 0.770 | 0.251 | 0.022 | < 0.001 |
| CYB5R3 | 0.767 | 0.442 | 0.308 | < 0.001 |
| RILPL2 | 0.765 | 0.370 | 0.133 | < 0.001 |
| SSFA2 | 0.763 | 0.298 | 0.134 | < 0.001 |
| MACF1 | 0.761 | 0.444 | 0.290 | < 0.001 |
| GRASP | 0.760 | 0.226 | 0.016 | < 0.001 |
| C2CD4B | 0.759 | 0.164 | 0.007 | < 0.001 |
| CFAP20 | 0.759 | 0.296 | 0.171 | < 0.001 |
| LRCH1 | 0.757 | 0.263 | 0.056 | < 0.001 |
| CTNNAL1 | 0.756 | 0.324 | 0.203 | < 0.001 |
| CMIP | 0.755 | 0.323 | 0.117 | < 0.001 |
| ADAMTS4 | 0.755 | 0.181 | 0.023 | < 0.001 |
| ARID5A | 0.754 | 0.257 | 0.072 | < 0.001 |
| CYR61 | 0.754 | 0.379 | 0.312 | < 0.001 |
| DUSP5 | 0.753 | 0.297 | 0.065 | < 0.001 |
| PRKCH | 0.752 | 0.326 | 0.121 | < 0.001 |
| SYNPO | 0.752 | 0.240 | 0.010 | < 0.001 |
| KANK3 | 0.752 | 0.226 | 0.007 | < 0.001 |
| PTMA | 0.751 | 0.997 | 0.977 | < 0.001 |
| KLHL5 | 0.750 | 0.244 | 0.036 | < 0.001 |
| DGKH | 0.749 | 0.256 | 0.036 | < 0.001 |
| ERICH1 | 0.749 | 0.368 | 0.161 | < 0.001 |
| RAB11A | 0.748 | 0.558 | 0.478 | < 0.001 |
| LY96 | 0.746 | 0.254 | 0.048 | < 0.001 |
| MX1 | 0.745 | 0.225 | 0.026 | < 0.001 |
| FAM43A | 0.745 | 0.306 | 0.131 | < 0.001 |
| CPNE8 | 0.744 | 0.239 | 0.012 | < 0.001 |
| DOCK6 | 0.743 | 0.249 | 0.034 | < 0.001 |
| CNKSR3 | 0.742 | 0.290 | 0.188 | < 0.001 |
| TRIM56 | 0.742 | 0.425 | 0.311 | < 0.001 |
| PREX1 | 0.742 | 0.260 | 0.049 | < 0.001 |
| RHOA | 0.742 | 0.720 | 0.642 | < 0.001 |
| PNP | 0.741 | 0.298 | 0.145 | < 0.001 |
| STAT3 | 0.741 | 0.625 | 0.506 | < 0.001 |
| TAX1BP3 | 0.741 | 0.333 | 0.138 | < 0.001 |
| SDCBP | 0.740 | 0.633 | 0.551 | < 0.001 |
| ARHGDIB | 0.739 | 0.552 | 0.203 | < 0.001 |
| KIAA1462 | 0.739 | 0.262 | 0.045 | < 0.001 |
| CTTNBP2NL | 0.738 | 0.280 | 0.085 | < 0.001 |
| PKP4 | 0.738 | 0.298 | 0.154 | < 0.001 |
| TRIOBP | 0.737 | 0.338 | 0.199 | < 0.001 |
| CCDC50 | 0.735 | 0.427 | 0.309 | < 0.001 |
| HPCAL1 | 0.735 | 0.325 | 0.163 | < 0.001 |
| LAMC1 | 0.733 | 0.287 | 0.137 | < 0.001 |
| MAP4 | 0.733 | 0.356 | 0.219 | < 0.001 |
| ICAM1 | 0.732 | 0.319 | 0.191 | < 0.001 |
| FGFR1 | 0.731 | 0.293 | 0.099 | < 0.001 |
| APOL3 | 0.730 | 0.245 | 0.031 | < 0.001 |
| TNFRSF1A | 0.728 | 0.337 | 0.219 | < 0.001 |
| C4orf32 | 0.728 | 0.310 | 0.148 | < 0.001 |
| FKBP5 | 0.728 | 0.525 | 0.354 | < 0.001 |
| PLA2G16 | 0.726 | 0.316 | 0.089 | < 0.001 |
| CTNNB1 | 0.726 | 0.524 | 0.451 | < 0.001 |
| PICALM | 0.725 | 0.362 | 0.208 | < 0.001 |
| ECE1 | 0.725 | 0.336 | 0.190 | < 0.001 |
| DNAJC15 | 0.723 | 0.325 | 0.080 | < 0.001 |
| TMEM109 | 0.722 | 0.361 | 0.215 | < 0.001 |
| SRGN | 0.721 | 0.757 | 0.254 | < 0.001 |
| SOX7 | 0.721 | 0.198 | 0.011 | < 0.001 |
| PLEKHA1 | 0.720 | 0.302 | 0.142 | < 0.001 |
| ZNF385D | 0.719 | 0.158 | 0.011 | < 0.001 |
| MCF2L | 0.718 | 0.253 | 0.075 | < 0.001 |
| SEPT7 | 0.718 | 0.562 | 0.376 | < 0.001 |
| LDHA | 0.717 | 0.681 | 0.523 | < 0.001 |
| ADGRG1 | 0.717 | 0.277 | 0.107 | < 0.001 |
| CD320 | 0.715 | 0.374 | 0.281 | < 0.001 |
| SLC44A2 | 0.714 | 0.325 | 0.154 | < 0.001 |
| DGKZ | 0.713 | 0.255 | 0.076 | < 0.001 |
| OSMR | 0.711 | 0.221 | 0.031 | < 0.001 |
| ITGA10 | 0.710 | 0.205 | 0.009 | < 0.001 |
| PREX2 | 0.709 | 0.214 | 0.005 | < 0.001 |
| CD40 | 0.707 | 0.258 | 0.028 | < 0.001 |
| KTN1 | 0.707 | 0.709 | 0.628 | < 0.001 |
| RGS3 | 0.706 | 0.219 | 0.035 | < 0.001 |
| ANKRD11 | 0.705 | 0.435 | 0.321 | < 0.001 |
| ADAMTS6 | 0.704 | 0.171 | 0.026 | < 0.001 |
| TSC22D3 | 0.704 | 0.746 | 0.518 | < 0.001 |
| ITPRIP | 0.703 | 0.256 | 0.029 | < 0.001 |
| SHANK3 | 0.702 | 0.209 | 0.006 | < 0.001 |
| PLEKHG1 | 0.702 | 0.225 | 0.005 | < 0.001 |
| RHOJ | 0.698 | 0.224 | 0.006 | < 0.001 |
| NCOA3 | 0.698 | 0.321 | 0.147 | < 0.001 |
| CALCOCO2 | 0.698 | 0.339 | 0.194 | < 0.001 |
| PEA15 | 0.695 | 0.362 | 0.216 | < 0.001 |
| FOXC1 | 0.694 | 0.244 | 0.056 | < 0.001 |
| PTPRM | 0.694 | 0.351 | 0.165 | < 0.001 |
| MLKL | 0.693 | 0.226 | 0.022 | < 0.001 |
| KLF10 | 0.692 | 0.323 | 0.217 | < 0.001 |
| LY6E | 0.691 | 0.491 | 0.334 | < 0.001 |
| TPST2 | 0.689 | 0.240 | 0.084 | < 0.001 |
| ABCB1 | 0.689 | 0.194 | 0.007 | < 0.001 |
| ADD1 | 0.689 | 0.353 | 0.241 | < 0.001 |
| MYL6 | 0.689 | 0.945 | 0.888 | < 0.001 |
| MEF2A | 0.688 | 0.286 | 0.119 | < 0.001 |
| SEC62 | 0.687 | 0.765 | 0.676 | < 0.001 |
| HOXB7 | 0.686 | 0.232 | 0.021 | < 0.001 |
| LDHB | 0.686 | 0.464 | 0.177 | < 0.001 |
| AHNAK | 0.686 | 0.578 | 0.464 | < 0.001 |
| SMTN | 0.685 | 0.254 | 0.108 | < 0.001 |
| SNAI1 | 0.684 | 0.212 | 0.031 | < 0.001 |
| PKIG | 0.684 | 0.296 | 0.121 | < 0.001 |
| TMOD3 | 0.683 | 0.361 | 0.220 | < 0.001 |
| SERPINE1 | 0.680 | 0.161 | 0.012 | < 0.001 |
| ACACB | 0.679 | 0.188 | 0.013 | < 0.001 |
| FLI1 | 0.678 | 0.243 | 0.024 | < 0.001 |
| IL4R | 0.677 | 0.269 | 0.054 | < 0.001 |
| PAM | 0.677 | 0.259 | 0.074 | < 0.001 |
| MT1M | 0.674 | 0.221 | 0.104 | < 0.001 |
| SHE | 0.671 | 0.219 | 0.002 | < 0.001 |
| ST6GAL1 | 0.671 | 0.280 | 0.094 | < 0.001 |
| FOS | 0.668 | 0.844 | 0.826 | < 0.001 |
| RASGRP3 | 0.668 | 0.203 | 0.033 | < 0.001 |
| PSIP1 | 0.667 | 0.340 | 0.169 | < 0.001 |
| APLNR | 0.665 | 0.160 | 0.001 | < 0.001 |
| CHD9 | 0.665 | 0.405 | 0.260 | < 0.001 |
| CD55 | 0.664 | 0.315 | 0.113 | < 0.001 |
| RCAN1 | 0.664 | 0.165 | 0.055 | < 0.001 |
| CARD16 | 0.663 | 0.307 | 0.067 | < 0.001 |
| IFIT3 | 0.663 | 0.186 | 0.020 | < 0.001 |
| SERINC3 | 0.663 | 0.481 | 0.398 | < 0.001 |
| HIP1 | 0.662 | 0.274 | 0.054 | < 0.001 |
| LAPTM4B | 0.661 | 0.270 | 0.094 | < 0.001 |
| QKI | 0.660 | 0.348 | 0.202 | < 0.001 |
| EBF1 | 0.659 | 0.236 | 0.028 | < 0.001 |
| SLFN5 | 0.659 | 0.289 | 0.101 | < 0.001 |
| SH2D3C | 0.658 | 0.202 | 0.007 | < 0.001 |
| GOLIM4 | 0.658 | 0.306 | 0.168 | < 0.001 |
| IPO11 | 0.657 | 0.228 | 0.037 | < 0.001 |
| ATOH8 | 0.657 | 0.193 | 0.021 | < 0.001 |
| CPNE2 | 0.655 | 0.232 | 0.024 | < 0.001 |
| IFI44 | 0.654 | 0.236 | 0.032 | < 0.001 |
| OAZ2 | 0.654 | 0.406 | 0.356 | < 0.001 |
| FGD5 | 0.653 | 0.210 | 0.002 | < 0.001 |
| SNRK | 0.652 | 0.287 | 0.096 | < 0.001 |
| NDUFA12 | 0.650 | 0.468 | 0.390 | < 0.001 |
| MBNL1 | 0.650 | 0.422 | 0.280 | < 0.001 |
| EFNA1 | 0.649 | 0.417 | 0.360 | < 0.001 |
| TCN2 | 0.649 | 0.201 | 0.024 | < 0.001 |
| MAP4K4 | 0.649 | 0.313 | 0.199 | < 0.001 |
| SNTB2 | 0.649 | 0.331 | 0.223 | < 0.001 |
| MYO1C | 0.648 | 0.363 | 0.284 | < 0.001 |
| HMBOX1 | 0.647 | 0.278 | 0.121 | < 0.001 |
| EPHX1 | 0.647 | 0.326 | 0.205 | < 0.001 |
| ADM5 | 0.646 | 0.150 | 0.008 | < 0.001 |
| EVA1B | 0.646 | 0.243 | 0.061 | < 0.001 |
| WASF2 | 0.645 | 0.530 | 0.474 | < 0.001 |
| PARP14 | 0.643 | 0.287 | 0.121 | < 0.001 |
| ABI3 | 0.643 | 0.229 | 0.031 | < 0.001 |
| FYN | 0.643 | 0.335 | 0.066 | < 0.001 |
| GPX3 | 0.643 | 0.232 | 0.047 | < 0.001 |
| CARHSP1 | 0.643 | 0.344 | 0.219 | < 0.001 |
| ODF2L | 0.642 | 0.346 | 0.191 | < 0.001 |
| CAPZA2 | 0.641 | 0.495 | 0.423 | < 0.001 |
| RGL1 | 0.639 | 0.196 | 0.020 | < 0.001 |
| TTC28 | 0.637 | 0.236 | 0.037 | < 0.001 |
| CYSTM1 | 0.637 | 0.410 | 0.290 | < 0.001 |
| SHC1 | 0.635 | 0.297 | 0.170 | < 0.001 |
| JAG2 | 0.635 | 0.205 | 0.022 | < 0.001 |
| RGS16 | 0.634 | 0.183 | 0.079 | < 0.001 |
| GGT5 | 0.633 | 0.195 | 0.013 | < 0.001 |
| CRK | 0.632 | 0.356 | 0.252 | < 0.001 |
| ARAP3 | 0.632 | 0.197 | 0.004 | < 0.001 |
| AFF1 | 0.631 | 0.311 | 0.167 | < 0.001 |
| POLE4 | 0.631 | 0.383 | 0.296 | < 0.001 |
| GSDMD | 0.631 | 0.268 | 0.068 | < 0.001 |
| INPP1 | 0.631 | 0.223 | 0.024 | < 0.001 |
| SORBS2 | 0.630 | 0.309 | 0.210 | < 0.001 |
| CFH | 0.630 | 0.177 | 0.010 | < 0.001 |
| LAP3 | 0.629 | 0.282 | 0.155 | < 0.001 |
| WARS | 0.629 | 0.249 | 0.133 | < 0.001 |
| ARHGAP18 | 0.628 | 0.282 | 0.162 | < 0.001 |
| PIEZO2 | 0.627 | 0.153 | 0.002 | < 0.001 |
| BTN3A2 | 0.626 | 0.287 | 0.080 | < 0.001 |
| INHBB | 0.626 | 0.174 | 0.069 | < 0.001 |
| SEMA3G | 0.625 | 0.123 | 0.004 | < 0.001 |
| BNIP2 | 0.622 | 0.328 | 0.157 | < 0.001 |
| CARD8 | 0.621 | 0.255 | 0.082 | < 0.001 |
| SPTAN1 | 0.618 | 0.355 | 0.242 | < 0.001 |
| ACE | 0.617 | 0.171 | 0.022 | < 0.001 |
| HOXB4 | 0.616 | 0.208 | 0.015 | < 0.001 |
| CEP68 | 0.616 | 0.237 | 0.062 | < 0.001 |
| GNAQ | 0.614 | 0.452 | 0.396 | < 0.001 |
| COL18A1 | 0.612 | 0.279 | 0.065 | < 0.001 |
| ITGA8 | 0.611 | 0.191 | 0.013 | < 0.001 |
| FLT4 | 0.611 | 0.182 | 0.002 | < 0.001 |
| PROCR | 0.610 | 0.209 | 0.021 | < 0.001 |
| RHOB | 0.610 | 0.709 | 0.616 | < 0.001 |
| GALNT18 | 0.609 | 0.184 | 0.013 | < 0.001 |
| SEPT10 | 0.608 | 0.247 | 0.105 | < 0.001 |
| RAPGEF5 | 0.607 | 0.200 | 0.023 | < 0.001 |
| CASP4 | 0.606 | 0.286 | 0.108 | < 0.001 |
| RNF213 | 0.605 | 0.448 | 0.327 | < 0.001 |
| LMNA | 0.604 | 0.641 | 0.497 | < 0.001 |
| PNISR | 0.604 | 0.613 | 0.538 | < 0.001 |
| CLEC1A | 0.604 | 0.167 | 0.001 | < 0.001 |
| AFAP1L1 | 0.602 | 0.185 | 0.003 | < 0.001 |
| EVA1C | 0.602 | 0.219 | 0.048 | < 0.001 |
| BAZ2B | 0.602 | 0.337 | 0.221 | < 0.001 |
| SELM | 0.601 | 0.556 | 0.368 | < 0.001 |
| IRF2BP2 | 0.600 | 0.465 | 0.404 | < 0.001 |
| CSRNP1 | 0.600 | 0.389 | 0.288 | < 0.001 |
| SERTAD4-AS1 | 0.600 | 0.172 | 0.011 | < 0.001 |
| MAPK3 | 0.599 | 0.256 | 0.087 | < 0.001 |
| FLOT1 | 0.598 | 0.422 | 0.354 | < 0.001 |
| ZFYVE21 | 0.598 | 0.327 | 0.248 | < 0.001 |
| MMRN1 | 0.597 | 0.111 | 0.001 | < 0.001 |
| ERAP2 | 0.597 | 0.253 | 0.081 | < 0.001 |
| SP100 | 0.595 | 0.359 | 0.170 | < 0.001 |
| ARRDC2 | 0.591 | 0.219 | 0.052 | < 0.001 |
| SH3GLB1 | 0.590 | 0.393 | 0.303 | < 0.001 |
| CFDP1 | 0.590 | 0.363 | 0.301 | < 0.001 |
| RSU1 | 0.589 | 0.284 | 0.134 | < 0.001 |
| TNFAIP1 | 0.587 | 0.233 | 0.079 | < 0.001 |
| PML | 0.587 | 0.235 | 0.076 | < 0.001 |
| CLSTN3 | 0.586 | 0.180 | 0.023 | < 0.001 |
| CD63 | 0.586 | 0.873 | 0.773 | < 0.001 |
| HIST1H4C | 0.584 | 0.483 | 0.283 | < 0.001 |
| RALB | 0.583 | 0.274 | 0.129 | < 0.001 |
| CD151 | 0.583 | 0.601 | 0.504 | < 0.001 |
| LRRC8C | 0.583 | 0.209 | 0.021 | < 0.001 |
| WDR60 | 0.582 | 0.302 | 0.227 | < 0.001 |
| CLIC2 | 0.582 | 0.176 | 0.013 | < 0.001 |
| ARHGEF12 | 0.582 | 0.326 | 0.226 | < 0.001 |
| TCF7L1 | 0.581 | 0.190 | 0.009 | < 0.001 |
| RAPGEF2 | 0.581 | 0.250 | 0.140 | < 0.001 |
| SULF1 | 0.581 | 0.116 | 0.009 | < 0.001 |
| PLCB1 | 0.581 | 0.214 | 0.077 | < 0.001 |
| FBLN2 | 0.581 | 0.106 | 0.004 | < 0.001 |
| CD79B | 0.580 | 0.217 | 0.017 | < 0.001 |
| RPS6KA2 | 0.577 | 0.258 | 0.141 | < 0.001 |
| BCR | 0.577 | 0.217 | 0.079 | < 0.001 |
| ITGB4 | 0.577 | 0.162 | 0.011 | < 0.001 |
| QSOX1 | 0.576 | 0.219 | 0.070 | < 0.001 |
| PLLP | 0.576 | 0.140 | 0.014 | < 0.001 |
| HLA-DPB1 | 0.575 | 0.663 | 0.239 | < 0.001 |
| DOCK4 | 0.574 | 0.228 | 0.102 | < 0.001 |
| BOC | 0.573 | 0.174 | 0.019 | < 0.001 |
| CPLX1 | 0.573 | 0.169 | 0.015 | < 0.001 |
| FAM171A1 | 0.573 | 0.201 | 0.087 | < 0.001 |
| IRF9 | 0.571 | 0.251 | 0.080 | < 0.001 |
| DDX3X | 0.571 | 0.551 | 0.514 | < 0.001 |
| ASAP2 | 0.568 | 0.216 | 0.074 | < 0.001 |
| CDC42EP3 | 0.568 | 0.299 | 0.184 | < 0.001 |
| NOVA2 | 0.567 | 0.179 | 0.001 | < 0.001 |
| NUCB1 | 0.567 | 0.454 | 0.380 | < 0.001 |
| SERPINB1 | 0.567 | 0.273 | 0.078 | < 0.001 |
| SRSF4 | 0.566 | 0.392 | 0.322 | < 0.001 |
| PITPNC1 | 0.566 | 0.218 | 0.053 | < 0.001 |
| TSPAN18 | 0.565 | 0.181 | 0.003 | < 0.001 |
| RBMS2 | 0.565 | 0.204 | 0.035 | < 0.001 |
| NOTCH1 | 0.564 | 0.207 | 0.038 | < 0.001 |
| SH3BGRL2 | 0.562 | 0.193 | 0.071 | < 0.001 |
| HRCT1 | 0.562 | 0.171 | 0.020 | < 0.001 |
| BTBD3 | 0.562 | 0.211 | 0.063 | < 0.001 |
| HOXD8 | 0.562 | 0.180 | 0.004 | < 0.001 |
| CNRIP1 | 0.561 | 0.225 | 0.026 | < 0.001 |
| LINC01420 | 0.560 | 0.438 | 0.362 | < 0.001 |
| WNK1 | 0.559 | 0.367 | 0.305 | < 0.001 |
| HLA-DQB1 | 0.558 | 0.365 | 0.080 | < 0.001 |
| CRHBP | 0.558 | 0.114 | 0.004 | < 0.001 |
| RSF1 | 0.558 | 0.415 | 0.353 | < 0.001 |
| RBM17 | 0.558 | 0.416 | 0.353 | < 0.001 |
| GABARAPL2 | 0.557 | 0.660 | 0.606 | < 0.001 |
| PIEZO1 | 0.555 | 0.262 | 0.164 | < 0.001 |
| ATP1A1 | 0.555 | 0.531 | 0.505 | < 0.001 |
| MORF4L1 | 0.554 | 0.674 | 0.633 | < 0.001 |
| APOL1 | 0.554 | 0.182 | 0.026 | < 0.001 |
| SPAG9 | 0.553 | 0.339 | 0.221 | < 0.001 |
| TAP1 | 0.553 | 0.243 | 0.106 | < 0.001 |
| CAPNS1 | 0.551 | 0.481 | 0.436 | < 0.001 |
| UBE2L6 | 0.549 | 0.259 | 0.084 | < 0.001 |
| BCAP31 | 0.549 | 0.549 | 0.514 | < 0.001 |
| ADIRF | 0.549 | 0.695 | 0.213 | < 0.001 |
| PVRL2 | 0.548 | 0.364 | 0.307 | < 0.001 |
| GADD45A | 0.547 | 0.306 | 0.158 | < 0.001 |
| PXN | 0.547 | 0.248 | 0.129 | < 0.001 |
| VGLL4 | 0.546 | 0.316 | 0.218 | < 0.001 |
| DYNC1LI2 | 0.546 | 0.382 | 0.325 | < 0.001 |
| AGRN | 0.545 | 0.219 | 0.100 | < 0.001 |
| YPEL2 | 0.545 | 0.283 | 0.185 | < 0.001 |
| KRTCAP2 | 0.544 | 0.556 | 0.499 | < 0.001 |
| NFIA | 0.543 | 0.449 | 0.399 | < 0.001 |
| GSTO1 | 0.541 | 0.422 | 0.330 | < 0.001 |
| EHD2 | 0.540 | 0.221 | 0.031 | < 0.001 |
| LRRC8A | 0.540 | 0.292 | 0.197 | < 0.001 |
| GABRD | 0.540 | 0.126 | 0.003 | < 0.001 |
| NFE2L1 | 0.539 | 0.300 | 0.229 | < 0.001 |
| PARVB | 0.538 | 0.228 | 0.082 | < 0.001 |
| ARHGAP31 | 0.538 | 0.180 | 0.007 | < 0.001 |
| LAYN | 0.537 | 0.168 | 0.010 | < 0.001 |
| GPR146 | 0.537 | 0.151 | 0.018 | < 0.001 |
| RAB5C | 0.536 | 0.438 | 0.378 | < 0.001 |
| DDX21 | 0.536 | 0.522 | 0.501 | < 0.001 |
| GFOD2 | 0.535 | 0.227 | 0.116 | < 0.001 |
| RIN2 | 0.535 | 0.196 | 0.061 | < 0.001 |
| TANC1 | 0.535 | 0.246 | 0.126 | < 0.001 |
| LPCAT4 | 0.534 | 0.153 | 0.015 | < 0.001 |
| LGALS3 | 0.532 | 0.432 | 0.187 | < 0.001 |
| ZFAND5 | 0.532 | 0.576 | 0.511 | < 0.001 |
| C9orf3 | 0.532 | 0.245 | 0.074 | < 0.001 |
| ITPKC | 0.532 | 0.228 | 0.126 | < 0.001 |
| LAMB1 | 0.532 | 0.168 | 0.019 | < 0.001 |
| NQO1 | 0.531 | 0.177 | 0.053 | < 0.001 |
| MPHOSPH8 | 0.530 | 0.498 | 0.458 | < 0.001 |
| CALHM2 | 0.529 | 0.198 | 0.022 | < 0.001 |
| EPHB4 | 0.529 | 0.186 | 0.069 | < 0.001 |
| AKAP13 | 0.529 | 0.433 | 0.298 | < 0.001 |
| RUNDC3B | 0.529 | 0.143 | 0.005 | < 0.001 |
| GPRC5B | 0.529 | 0.168 | 0.007 | < 0.001 |
| VAT1 | 0.529 | 0.243 | 0.102 | < 0.001 |
| PDCD4 | 0.527 | 0.436 | 0.411 | < 0.001 |
| GBP1 | 0.527 | 0.175 | 0.031 | < 0.001 |
| NKTR | 0.526 | 0.466 | 0.437 | < 0.001 |
| STAB1 | 0.525 | 0.159 | 0.015 | < 0.001 |
| LRRC1 | 0.525 | 0.160 | 0.094 | < 0.001 |
| PLK3 | 0.523 | 0.327 | 0.174 | < 0.001 |
| IKBIP | 0.523 | 0.197 | 0.041 | < 0.001 |
| CCDC69 | 0.522 | 0.172 | 0.025 | < 0.001 |
| EML1 | 0.522 | 0.175 | 0.029 | < 0.001 |
| STC2 | 0.522 | 0.151 | 0.012 | < 0.001 |
| CLEC3B | 0.521 | 0.149 | 0.002 | < 0.001 |
| DLL1 | 0.519 | 0.160 | 0.021 | < 0.001 |
| FUS | 0.518 | 0.651 | 0.628 | < 0.001 |
| PHACTR4 | 0.517 | 0.326 | 0.262 | < 0.001 |
| CCPG1 | 0.517 | 0.341 | 0.273 | < 0.001 |
| MAP3K6 | 0.517 | 0.176 | 0.041 | < 0.001 |
| LRRFIP1 | 0.516 | 0.637 | 0.581 | < 0.001 |
| GNG5 | 0.515 | 0.668 | 0.622 | < 0.001 |
| CAP1 | 0.514 | 0.489 | 0.431 | < 0.001 |
| ACTN1 | 0.513 | 0.239 | 0.185 | < 0.001 |
| VOPP1 | 0.512 | 0.269 | 0.138 | < 0.001 |
| TNFAIP8L1 | 0.512 | 0.172 | 0.022 | < 0.001 |
| HOXD9 | 0.511 | 0.149 | 0.003 | < 0.001 |
| GPR4 | 0.510 | 0.165 | 0.005 | < 0.001 |
| PON2 | 0.510 | 0.254 | 0.171 | < 0.001 |
| PIAS1 | 0.509 | 0.292 | 0.228 | < 0.001 |
| SEMA6B | 0.509 | 0.166 | 0.009 | < 0.001 |
| CTNNA1 | 0.509 | 0.378 | 0.349 | < 0.001 |
| TAOK2 | 0.509 | 0.195 | 0.075 | < 0.001 |
| MSX1 | 0.509 | 0.170 | 0.057 | < 0.001 |
| PPP1R10 | 0.509 | 0.374 | 0.337 | < 0.001 |
| CMTM8 | 0.509 | 0.231 | 0.117 | < 0.001 |
| UGCG | 0.509 | 0.295 | 0.261 | < 0.001 |
| ATXN3 | 0.509 | 0.276 | 0.183 | < 0.001 |
| HLF | 0.507 | 0.115 | 0.007 | < 0.001 |
| MBNL2 | 0.507 | 0.217 | 0.064 | < 0.001 |
| COL21A1 | 0.506 | 0.149 | 0.004 | < 0.001 |
| PLEKHO2 | 0.506 | 0.182 | 0.039 | < 0.001 |
| FMO2 | 0.505 | 0.104 | 0.003 | < 0.001 |
| DYNC1I2 | 0.505 | 0.422 | 0.389 | < 0.001 |
| NSRP1 | 0.504 | 0.353 | 0.321 | < 0.001 |
| CDC42EP4 | 0.503 | 0.221 | 0.090 | < 0.001 |
| SEMA6A | 0.502 | 0.160 | 0.029 | < 0.001 |
| PCSK5 | 0.502 | 0.113 | 0.007 | < 0.001 |
| CALD1 | 0.502 | 0.690 | 0.355 | < 0.001 |
| MGAT4A | 0.501 | 0.289 | 0.177 | < 0.001 |
| CCDC3 | 0.501 | 0.141 | 0.013 | < 0.001 |
| SLC35G2 | 0.501 | 0.169 | 0.012 | < 0.001 |
| TMEM50A | 0.500 | 0.573 | 0.541 | < 0.001 |
| A total of 54 recurrence-associated endothelial cell related genes are highlighted in color with blue and grey indicating the signature genes and remaining genes, respectively. | | | | |

| **Table S7 Univariate Cox regression analysis of 54 RAEC-related genes in TCGA-PRAD** | | | | |
| --- | --- | --- | --- | --- |
| Gene | Hazard ratio | Lower 95% CI | Upper 95% CI | P value |
| GABRD | 1.593 | 1.281 | 1.982 | 0.000 |
| ESM1 | 1.297 | 1.136 | 1.481 | 0.000 |
| FAM107A | 0.694 | 0.575 | 0.837 | 0.000 |
| DOCK6 | 2.264 | 1.465 | 3.501 | 0.000 |
| APLNR | 1.393 | 1.142 | 1.699 | 0.001 |
| TMEM255B | 1.905 | 1.280 | 2.835 | 0.001 |
| FSCN1 | 1.579 | 1.186 | 2.102 | 0.002 |
| EXOC3L2 | 1.414 | 1.114 | 1.796 | 0.004 |
| CA2 | 1.292 | 1.076 | 1.553 | 0.006 |
| SDPR | 0.766 | 0.630 | 0.930 | 0.007 |
| HLF | 0.728 | 0.576 | 0.920 | 0.008 |
| POSTN | 1.234 | 1.041 | 1.464 | 0.016 |
| TGFBR3 | 0.758 | 0.601 | 0.956 | 0.019 |
| TNFAIP8L1 | 1.480 | 1.020 | 2.147 | 0.039 |
| FLT4 | 1.389 | 1.017 | 1.896 | 0.039 |
| EDNRB | 0.827 | 0.683 | 1.001 | 0.051 |
| BTNL9 | 1.345 | 0.996 | 1.817 | 0.053 |
| LEPR | 0.793 | 0.619 | 1.016 | 0.067 |
| GPM6A | 0.767 | 0.568 | 1.036 | 0.084 |
| HECW2 | 1.372 | 0.956 | 1.968 | 0.086 |
| SPRY4 | 1.139 | 0.946 | 1.373 | 0.170 |
| IL33 | 0.877 | 0.717 | 1.071 | 0.198 |
| RND1 | 0.886 | 0.733 | 1.072 | 0.213 |
| COL15A1 | 1.136 | 0.923 | 1.398 | 0.228 |
| PLVAP | 1.156 | 0.907 | 1.474 | 0.242 |
| TM4SF18 | 1.122 | 0.897 | 1.402 | 0.313 |
| RBP5 | 1.162 | 0.867 | 1.559 | 0.314 |
| PIEZO2 | 0.855 | 0.614 | 1.191 | 0.354 |
| GNG11 | 0.878 | 0.659 | 1.170 | 0.374 |
| DYSF | 0.887 | 0.677 | 1.161 | 0.382 |
| GJA1 | 0.928 | 0.768 | 1.122 | 0.442 |
| PDGFD | 0.918 | 0.722 | 1.166 | 0.481 |
| CCL14 | 0.904 | 0.682 | 1.200 | 0.485 |
| LMCD1 | 1.103 | 0.834 | 1.458 | 0.493 |
| SELP | 0.923 | 0.733 | 1.162 | 0.494 |
| FAM101B | 1.114 | 0.806 | 1.538 | 0.514 |
| LAYN | 1.079 | 0.827 | 1.408 | 0.576 |
| CDH13 | 1.090 | 0.796 | 1.494 | 0.591 |
| CTGF | 0.957 | 0.809 | 1.133 | 0.613 |
| FLT1 | 1.054 | 0.855 | 1.300 | 0.622 |
| TMEM204 | 1.041 | 0.839 | 1.291 | 0.717 |
| KLHL5 | 0.956 | 0.747 | 1.222 | 0.718 |
| PLAT | 0.978 | 0.839 | 1.139 | 0.772 |
| KDR | 0.976 | 0.790 | 1.205 | 0.820 |
| CCL23 | 0.975 | 0.783 | 1.214 | 0.821 |
| TMTC1 | 0.971 | 0.747 | 1.261 | 0.823 |
| CALCRL | 1.021 | 0.832 | 1.253 | 0.843 |
| FBLN2 | 0.982 | 0.819 | 1.177 | 0.845 |
| ACKR1 | 0.984 | 0.826 | 1.172 | 0.854 |
| S100A16 | 0.982 | 0.793 | 1.214 | 0.863 |
| CLDN5 | 0.983 | 0.783 | 1.233 | 0.879 |
| AFAP1L1 | 1.019 | 0.738 | 1.409 | 0.907 |
| FMO2 | 0.995 | 0.804 | 1.232 | 0.965 |
| FBLN5 | 1.005 | 0.798 | 1.266 | 0.967 |
| RAEC, recurrence-associated endothelial cell. RAEC signature genes were highlighted in light blue. | | | | |

| **Table S8 Results of nested cross validation in 11 machine-learning algorithms** | | |
| --- | --- | --- |
| Model | C-index | IBS |
| XGBoost | 0.638 | 0.208 |
|  | 0.740 | 0.099 |
|  | 0.594 | 0.203 |
|  | 0.848 | 0.109 |
|  | 0.762 | 0.155 |
|  | 0.598 | 0.175 |
|  | 0.492 | 0.163 |
|  | 0.712 | 0.143 |
|  | 0.771 | 0.142 |
|  | 0.760 | 0.117 |
| survival-SVM | 0.593 | 0.202 |
|  | 0.500 | 0.131 |
|  | 0.500 | 0.210 |
|  | 0.500 | 0.191 |
|  | 0.486 | 0.171 |
|  | 0.530 | 0.365 |
|  | 0.563 | 0.151 |
|  | 0.580 | 0.170 |
|  | 0.505 | 0.162 |
|  | 0.494 | 0.158 |
| SuperPC | 0.567 | 0.184 |
|  | 0.560 | 0.133 |
|  | 0.402 | 0.211 |
|  | 0.818 | 0.107 |
|  | 0.827 | 0.188 |
|  | 0.543 | 0.200 |
|  | 0.502 | 0.162 |
|  | 0.468 | 0.168 |
|  | 0.721 | 0.139 |
|  | 0.657 | 0.142 |
| StepCox (forward) | 0.619 | 0.215 |
|  | 0.700 | 0.107 |
|  | 0.545 | 0.195 |
|  | 0.801 | 0.103 |
|  | 0.664 | 0.143 |
|  | 0.581 | 0.184 |
|  | 0.479 | 0.163 |
|  | 0.644 | 0.168 |
|  | 0.725 | 0.173 |
|  | 0.718 | 0.137 |
| StepCox (both) | 0.657 | 0.221 |
|  | 0.725 | 0.105 |
|  | 0.545 | 0.199 |
|  | 0.808 | 0.108 |
|  | 0.715 | 0.135 |
|  | 0.568 | 0.181 |
|  | 0.514 | 0.164 |
|  | 0.656 | 0.164 |
|  | 0.743 | 0.162 |
|  | 0.724 | 0.128 |
| StepCox (backward) | 0.657 | 0.221 |
|  | 0.725 | 0.105 |
|  | 0.545 | 0.199 |
|  | 0.808 | 0.108 |
|  | 0.715 | 0.135 |
|  | 0.568 | 0.181 |
|  | 0.514 | 0.164 |
|  | 0.656 | 0.164 |
|  | 0.743 | 0.162 |
|  | 0.724 | 0.128 |
| RSF | 0.633 | 0.226 |
|  | 0.835 | 0.102 |
|  | 0.623 | 0.216 |
|  | 0.862 | 0.120 |
|  | 0.664 | 0.166 |
|  | 0.483 | 0.173 |
|  | 0.454 | 0.163 |
|  | 0.680 | 0.148 |
|  | 0.796 | 0.144 |
|  | 0.702 | 0.113 |
| Ridge | 0.662 | 0.198 |
|  | 0.745 | 0.106 |
|  | 0.594 | 0.218 |
|  | 0.862 | 0.103 |
|  | 0.664 | 0.159 |
|  | 0.585 | 0.172 |
|  | 0.527 | 0.164 |
|  | 0.680 | 0.151 |
|  | 0.807 | 0.150 |
|  | 0.691 | 0.130 |
| plsRcox | 0.619 | 0.218 |
|  | 0.700 | 0.104 |
|  | 0.545 | 0.208 |
|  | 0.801 | 0.097 |
|  | 0.654 | 0.146 |
|  | 0.581 | 0.182 |
|  | 0.483 | 0.164 |
|  | 0.644 | 0.149 |
|  | 0.725 | 0.168 |
|  | 0.718 | 0.116 |
| Lasso | 0.629 | 0.198 |
|  | 0.700 | 0.100 |
|  | 0.553 | 0.215 |
|  | 0.832 | 0.097 |
|  | 0.678 | 0.145 |
|  | 0.611 | 0.174 |
|  | 0.517 | 0.164 |
|  | 0.668 | 0.141 |
|  | 0.750 | 0.159 |
|  | 0.718 | 0.125 |
| GBM | 0.581 | 0.210 |
|  | 0.785 | 0.095 |
|  | 0.549 | 0.205 |
|  | 0.845 | 0.120 |
|  | 0.701 | 0.151 |
|  | 0.560 | 0.169 |
|  | 0.489 | 0.163 |
|  | 0.648 | 0.156 |
|  | 0.736 | 0.148 |
|  | 0.702 | 0.112 |
| Enet | 0.657 | 0.202 |
|  | 0.745 | 0.104 |
|  | 0.590 | 0.216 |
|  | 0.838 | 0.098 |
|  | 0.664 | 0.149 |
|  | 0.603 | 0.174 |
|  | 0.533 | 0.164 |
|  | 0.668 | 0.146 |
|  | 0.804 | 0.154 |
|  | 0.729 | 0.125 |
| CoxBoost | 0.638 | 0.204 |
|  | 0.715 | 0.097 |
|  | 0.578 | 0.216 |
|  | 0.835 | 0.098 |
|  | 0.678 | 0.146 |
|  | 0.594 | 0.172 |
|  | 0.524 | 0.164 |
|  | 0.660 | 0.145 |
|  | 0.761 | 0.157 |
|  | 0.724 | 0.124 |
| IBS, integrated Brier score |  |  |

| **Table S9 Comparison of RAECsig with clinical features in 6 PCa cohorts** | | | |
| --- | --- | --- | --- |
| Cohort | Variables | C-index | P value |
| TCGA-PRAD | RAECsig | 0.767 | NA |
|  | Age | 0.553 | 0.000 |
|  | pT | 0.622 | 0.000 |
|  | pN | 0.538 | 0.000 |
|  | Gleason score | 0.674 | 0.003 |
| DKFZ-PRAD | RAECsig | 0.836 | NA |
|  | Age | 0.509 | 0.000 |
|  | PSA | 0.819 | 0.731 |
|  | Gleason score | 0.650 | 0.002 |
|  | pT | 0.735 | 0.061 |
| GSE70768 | RAECsig | 0.769 | NA |
|  | Age | 0.543 | 0.000 |
|  | PSA | 0.583 | 0.000 |
|  | Gleason score | 0.599 | 0.008 |
|  | pT | 0.554 | 0.008 |
|  | pN | 0.522 | 0.001 |
| GSE70769 | RAECsig | 0.679 | NA |
|  | PSA | 0.561 | 0.044 |
|  | Gleason score | 0.613 | 0.160 |
|  | pT | 0.679 | 0.898 |
| GSE94767 | RAECsig | 0.641 | NA |
|  | PSA | 0.517 | 0.060 |
|  | Gleason score | 0.586 | 0.334 |
|  | pT | 0.595 | 0.478 |
| GSE21034 | RAECsig | 0.669 | NA |
|  | Gleason score | 0.712 | 0.349 |
|  | pT | 0.568 | 0.125 |

| **Table S10 Comparison of RAECsig with published genetic tests using univariate Cox regression and C-index analyses in multiple PCa cohorts** | | | | | | | |
| --- | --- | --- | --- | --- | --- | --- | --- |
| Cohort | Signature | Univariate Cox regression | | | | C-index comparion | |
|  |  | Hazard ratio | Lower 95% CI | Upper 95% CI | P value | C-index | P value |
| TCGA-PRAD | RAECsig | 6.872 | 4.721 | 10.004 | 0.000 | 0.767 | NA |
|  | Oncotype | 1.032 | 1.020 | 1.045 | 0.000 | 0.661 | 0.001 |
|  | Decipher | 1.150 | 1.044 | 1.267 | 0.004 | 0.588 | 0.000 |
|  | Prolaris | 1.095 | 1.057 | 1.135 | 0.000 | 0.653 | 0.000 |
| DKFZ-PRAD | RAECsig | 3.505 | 2.051 | 5.989 | 0.000 | 0.836 | NA |
|  | Oncotype | 1.048 | 1.029 | 1.067 | 0.000 | 0.832 | 0.926 |
|  | Decipher | 1.563 | 1.297 | 1.884 | 0.000 | 0.813 | 0.548 |
|  | Prolaris | 1.129 | 1.054 | 1.209 | 0.001 | 0.643 | 0.034 |
| GSE70768 | RAECsig | 9.034 | 3.250 | 25.111 | 0.000 | 0.769 | NA |
|  | Oncotype | 1.053 | 1.030 | 1.077 | 0.000 | 0.738 | 0.665 |
|  | Decipher | 1.440 | 1.131 | 1.832 | 0.003 | 0.696 | 0.236 |
|  | Prolaris | 1.189 | 1.094 | 1.292 | 0.000 | 0.673 | 0.172 |
| GSE70769 | RAECsig | 4.041 | 1.997 | 8.181 | 0.000 | 0.679 | NA |
|  | Oncotype | 1.034 | 1.019 | 1.050 | 0.000 | 0.707 | 0.447 |
|  | Decipher | 1.458 | 1.250 | 1.701 | 0.000 | 0.693 | 0.723 |
|  | Prolaris | 1.102 | 1.048 | 1.159 | 0.000 | 0.645 | 0.444 |
| GSE94767 | RAECsig | 3.014 | 1.545 | 5.881 | 0.001 | 0.641 | NA |
|  | Oncotype | 1.027 | 1.013 | 1.042 | 0.000 | 0.654 | 0.799 |
|  | Decipher | 1.335 | 1.129 | 1.579 | 0.001 | 0.650 | 0.888 |
|  | Prolaris | 1.125 | 1.053 | 1.203 | 0.001 | 0.651 | 0.847 |
| GSE21034 | RAECsig | 4.653 | 1.813 | 11.944 | 0.001 | 0.669 | NA |
|  | Oncotype | 1.038 | 1.020 | 1.055 | 0.000 | 0.738 | 0.176 |
|  | Decipher | 1.232 | 1.047 | 1.448 | 0.012 | 0.614 | 0.146 |
|  | Prolaris | 1.107 | 1.019 | 1.203 | 0.016 | 0.656 | 0.751 |
| Meta-GEO | RAECsig | 3.921 | 2.658 | 5.785 | 0.000 | 0.668 | NA |
|  | Oncotype | 1.040 | 1.031 | 1.049 | 0.000 | 0.702 | 0.191 |
|  | Decipher | 1.347 | 1.226 | 1.480 | 0.000 | 0.653 | 0.582 |
|  | Prolaris | 1.125 | 1.087 | 1.164 | 0.000 | 0.649 | 0.472 |
